# Supplementary material for: Closing the methane gap in US oil and natural gas production emissions inventories
Source: Nat Commun. 2021 Aug 5;12:4715. doi: 10.1038/s41467-021-25017-4 (PMC8342509; doi:10.1038/s41467-021-25017-4)
Supplement: Supplementary file 2 — Supplementary Information [file 41467_2021_25017_MOESM2_ESM.pdf]

Supplementary Information for:

Closing the methane gap in US oil and natural gas production emissions inventories

Jeffrey S. Rutherford, Evan D. Sherwin, Arvind P. Ravikumar, Garvin A. Heath, Jacob Englander, Daniel Cooley, David Lyon, Mark Omara, Quinn Langfitt, Adam R. Brandt

This document includes:

1. Supplementary Methods 1: Study scope and definitions 2
2. Supplementary Methods 2: Site-level studies 7
3. Supplementary Methods 3: Comparison of emission and activity factors 9
4. Supplementary Methods 4: The bottom-up CH_4 estimation tool 19
5. Supplementary Methods 5: Producing a production-segment CH_4 estimate 55
6. Supplementary Methods 6: Production segment CH_4 in the Greenhouse Gas Inventory 81
7. Supplementary Methods 7: Summary of surveyed studies 134
8. Supplementary Figures 147
9. Supplementary References 148

1. Supplementary Methods 1: Study scope and definitions

This study makes three contributions to the literature, each discussed in greater detail in Supplementary Information sections 4-6.

1. Development of a bottom-up CH_4 emissions estimation tool: In Supplementary Methods 4 we describe the bottom-up tool used in this study. First, we outline the underlying dataset which is composed of quantified emissions measurements, component counts, and fraction leaking estimates. Second, we describe the implementation of the tool as a subroutine in the life-cycle assessment model, OPGEE.

2. Generating a bottom-up estimate of US production-segment CH_4 emissions: In Supplementary Methods 5 we describe how our estimation tool, implemented in OPGEE, is used to produce an estimate of US CH_4 emissions from the production segment of the O&NG industry. This required development of a well-level dataset for implementation in our tool. For comparison with previous site-level results, we developed a site-level clustering algorithm which is also discussed in Section 5.

3. Derivation of Greenhouse Gas Inventory emission factors: The final contribution of this paper is an analysis of the EPA's emission factors applied in the Greenhouse Gas Inventory, described in Supplementary Methods 6. Through a literature review, we begin with the underlying datasets of quantified component-level emissions and reconstruct the emission factors found in the most recent inventory. Emission factors are compared with the emission factors applied in our tool (see comparison of component-level emission factors in **Supplementary Table 2**, comparison of equipment-level emission factors in **Supplementary Tables 3** and **4**, and a summary of component-level emission factors across the various studies in **Supplementary Table 5**).

1.1. Terminology

Classification schemes for CH_4 emissions differ across regulatory contexts and jurisdictions. In its inventory guidelines, the IPCC refers to "fugitives" as comprising "venting, flaring, and leaks" [1]. However, in the U.S. the term "fugitives" is often used interchangeably with "leaks" in referencing unintentional emissions from components such as valves, connectors, or open-ended lines (for example, where the former term is used in "fugitive emission VOC standards" in Subpart OOOOa Section 60.5397a [2] and the latter terms is used as a general category of emissions in Subpart W Section 98.233(q) [3] and the GHGI). In both Subpart W and the GHGI

venting emissions refer to the intentional release of gas from equipment blowdowns, pneumatic devices, and storage tanks.

To avoid confusion, we do not use the term "fugitives". To the extent possible, this study adopts the terminology conventions of the GHGI and the GHGRP with equipment leaks and vents. However, these terms should both be interpreted loosely. For example, our study integrates numerous data sources where quantified measurements are labelled by the source (e.g., valve or connector) but not by the purpose (i.e., intentional versus unintentional). Further, although the GHGI does not present additional categorization within oil and condensate tank vents (e.g., tanks with VRU versus tanks with flares, etc.), we differentiate between unintentional tank emissions and intentional tank emissions.

1.2. System Boundary

Our analysis boundary contains oil and natural gas (O&NG) production sites delivering marketable NG (including both associated and non-associated gas). We will refer to this as the production segment, which includes all equipment associated with a well pad and ends prior to centralized gathering and processing facilities (**Supplementary Fig. 1**). We limit our analysis to the production segment as this is the largest contributor to the divergence in emissions estimates between the GHGI and previous site level analyses [4] (**Supplementary Fig. 2**).

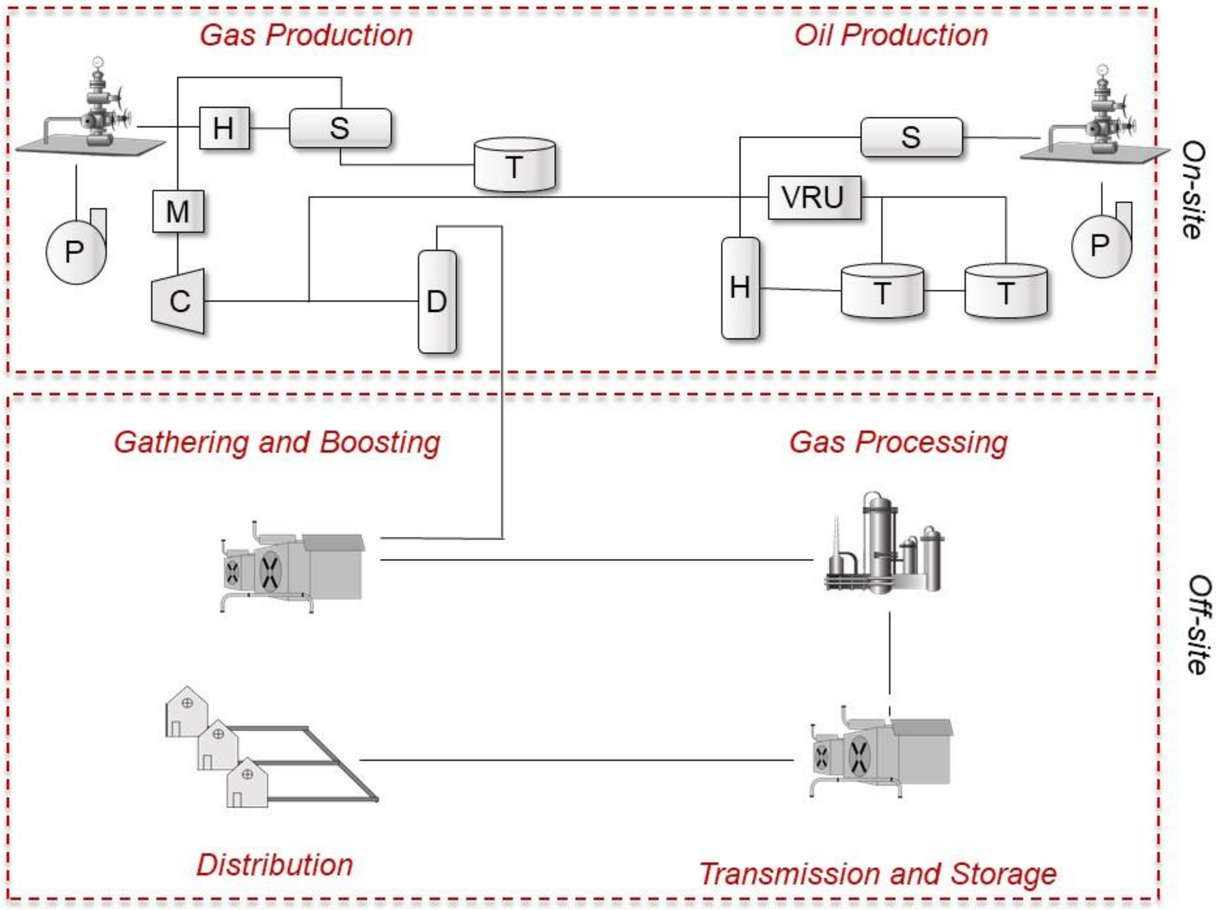

Supplementary Fig. 1: Illustration of the natural gas supply chain. This study will focus on the production segment, indicated in the figure with a red hashed box (H = heater, S = separator, T= tank, M = meter, P = pump, C = compressor, D = dehydrator, VRU = vapor recovery unit).

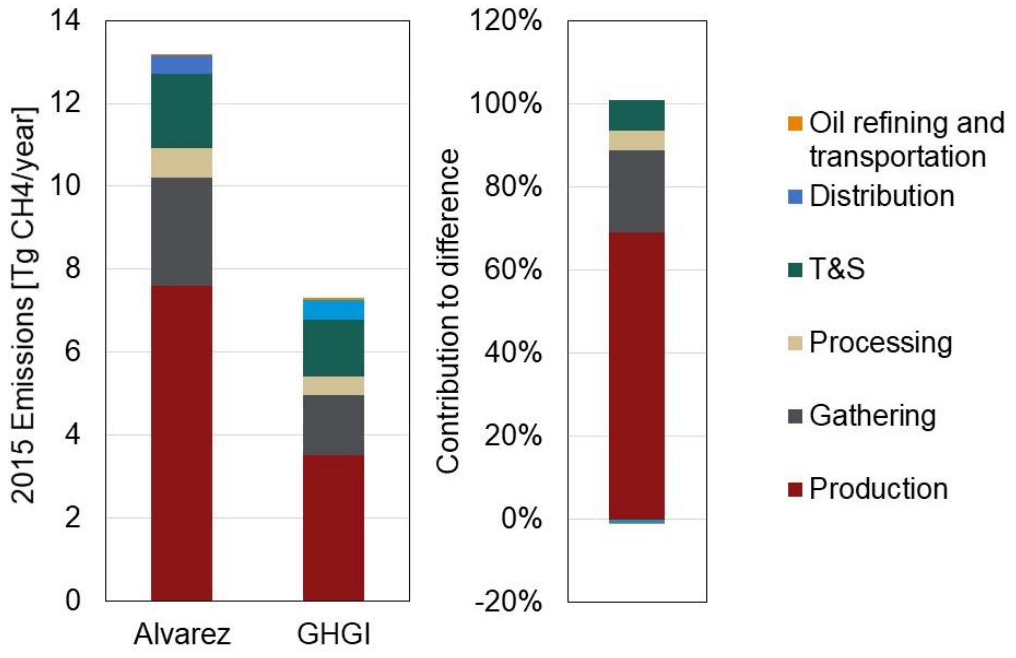

Supplementary Fig. 2: Comparison of site-level synthesis of oil and natural gas CH_4 emissions with the Greenhouse Gas Inventory. Alvarez et al. [4] synthesized site-level measurements of oil and natural gas CH_4 and compared with the EPA GHGI ([5] ,reporting year 2015). Right panel is percent contribution of supply chain segments to total difference.

We assess all emissions associated with production segment equipment. Categorization of equipment considers both capabilities of our model as well as categorization in the EPA Greenhouse Gas Inventory (GHGI). These categories are defined as follows:

- *Equipment leaks*: This category includes all component-level emissions associated with wellheads, separators, dehydrators, meters, reciprocating compressors, heaters, headers, chemical injection pumps. When aggregating GHGI data, dehydrator vents are also included in this category. Therefore, the term "leaks" should be interpreted loosely. Often studies will disaggregate emissions on the basis of leaks (unintentional emissions) and vents (intentional emissions). We do not do so, given that measurements in emissions quantification studies are often not labeled as fugitives or vents.
- *Pneumatic controllers*: Pneumatic controllers are found on various types of equipment. These components use gas pressure to actuate valves automating operation of flow, pressure, or liquid level. As the control valve is opened and closed, gas may be released. Although pneumatic controllers are technically components (versus an equipment unit)

the GHGI classifies pneumatic controller emissions separately instead of to the equipment on which they are installed. We follow this convention in this study.

- *Chemical injection pumps*: To be consistent with the EPA GHGI, we differentiate chemical injection pumps (referred to in Subpart W as natural gas driven pneumatic pumps [3]) from other pneumatic devices. According to Subpart W, pneumatic pumps "use pressurized natural gas to move a piston or diaphragm" [3]. These pumps are typically used to inject chemicals such as corrosion inhibitors,

- *Tanks*: Several types of emissions occur from crude and condensate storage tanks. Like other equipment, leaks occur through connectors, valves, and other components. Other emission mechanisms include working and breathing losses (solution gas evolved due to changes in temperature and pressure), flashing losses (solution gas released when liquids are dumped from the separator), or losses due to equipment malfunction (e.g., stuck dump valve on the separator).

- *Methane slip*: Small reciprocating compressors located on well pads have two types of emissions not found on other equipment: (i) leaks from rod-packing or centrifugal seals, and (ii) incomplete combustion from the gas engines used as prime movers. Leaks from rod-packing, seals, and other components are classified in the "equipment leaks" category. Methane slip refers to emissions associated with incomplete combustion.

- *Liquids unloadings*: Liquids unloadings are a type of infrequent emissions source and not all wells perform liquids unloadings. A liquids unloading is a procedure implemented periodically on gas producing wells to removed unwanted liquids (e.g., water, condensate, oil) from the well tubing using either a pump or by temporarily diverting flow to an atmospheric pressure tank to reduce the backpressure normally seen by the fluids and thereby inducing flow.

- *Completions and workovers*: Completions and workovers are another type of infrequent emissions source. A well completion is the process of transforming a drilled well into a producing well. Often a "completion" refers to the casing, cement, and wellhead assembly process, which are required before beginning production. During the well completion process, drilling mud, sand, and liquids must be removed from the well-bore. Gases, including methane, will be dissolved in the flowback liquids. These gases are either captured with flowback emission control devices or are emitted. After a well is completed, a workover may be required if equipment needs servicing or the well isn't producing efficiently. Like a completion, workovers can result in flowback-related emissions.

2. Supplementary Methods 2: Site-level studies

We validate our component-level bottom-up model by comparing total CH_4 emissions and emissions distributions with those generated in site-level studies (Alvarez et al. [4] and Omara et al. [6], respectively). Details of how results were prepared for this comparison are given in Supplementary Methods 5. Here, we will briefly summarize the studies compared.

Site-level studies rely on vehicles or other mobile laboratories equipped with CH_4 sensors downwind of well-pads. As the name implies, resolution is limited to aggregate emissions at the scale of a well-pad or processing facility. Methods include:

- *EPA Other Test Method (OTM 33A)*: An EPA OTM 33A assessment begins with an initial drive-by screening where the laboratory equipped vehicle searches for elevated CH_4 measurements. This screening is followed by a continuous measurement of CH_4 where the truck is parked downwind of the CH_4 emissions sources. Emissions are then estimated using a Gaussian approach. Examples of OTM 33A studies include Brantley et al. [7] and Roberson et al. [8].
- *Downwind tracer flux (DT)*: In a downwind tracer flux assessment, an atmospheric tracer (e.g., acetylene and nitrous oxide) is released near an emissions source and the mobile laboratory is driven downwind. Detailed post-hoc modelling is not required because it is assumed that dispersion of the tracer and CH_4 is similar. Examples include Goetz et al. [9] and Omara et al. [10].
- *Downwind measurements with Gaussian dispersion modelling (MMG)*: Some studies (e.g., Yacovitch et al. [11]) describe their approach as downwind measurements with Gaussian dispersion modelling. This approach is like the EPA Other Test Method 33A, with the notable exception that measurements can be taken while the vehicle is moving.

Omara et al. [6] synthesized results of eight production-segment studies: Brantley et al. [7], Eastern Research Group [12], Goetz et al. [9], Lan et al. [13], Omara et al. [10], Rella et al. [14], Robertson et al. [8], and Yacovitch et al. [11]. Some new measurements were also added. In total, the dataset includes site-level CH_4 emission measurements across 1009 sites (1 site = 1 measurement) and eight basins. Sampled basins include both gas producing regions (Marcellus, Fayetteville) and oil producing regions (Denver Julesburg, Eagle Ford).

The aim of the Omara study was to assess: (i) total CH_4 emissions from oil and natural-gas production sites, (ii) basin-to-basin differences in CH_4 emissions, and (iii) the relationship between CH_4 emissions and natural gas production. Omara et al. constructed a national-level

production-segment CH_4 emissions estimate by using a non-parametric bootstrap resampling approach. Before sampling, emissions measurements were grouped into ten bins based on deciles of natural gas production (Noting that Omara et al. do not estimate CH_4 emissions for wells reporting zero natural gas production, whereas Alvarez et al. do). The 498,000 US oil and natural gas production site assessed by Omara et al. (corresponding to approximately 813,615 wells, based on 2015 Enverus data) were binned into the same ten bins.

Note that the distribution of sampled sites in Omara et al. are generally higher production compared to the overall population of US sites (Fig S21 in Omara). Although this is accounted for in the model, low producing sites may not be characterized as well as high producing sites.

Supplementary Table 1: Summary of site-level data sets. Here we summarize studiesused in Omara et al and Alvarez et al (based upon statistics reported in Table S8 of Omara et al. [6]). Methods include EPA Other Test Method (OTM), downwind tracer flux (DT), and mobile monitoring with Gaussian modelling (MMG).

Study	Region	Omara	Alvarez	Number of sites	Measurement Technique	Site-level gas production (Mcfd), Min-Max
Omara et al. [10]	Marcellus	Y	Y	18	DT	0.68—44
Omara et al. [10]	Marcellus	Y	Y	13	DT	450—78,000
Brantley et al. [7]	Barnett	Y	Y	43	OTM	3.7—5,160
Brantley et al. [7]	Denver Julesberg	Y	Y	74	OTM	4.9—1,830
Brantley et al. [7]	Eagle Ford	Y	Y	4	OTM	78—2,000
Brantley et al. [7]	Pinedale	Y	Y	106	OTM	4.6—9,000
Rella et al. [14]	Barnett	Y	Y	185	MMG	2.3—6,000
ERG [12]	Fort Worth	Y		287	Onsite	0.4—39,300
Omara et al. [6]	Denver Julesberg	Y		18	DT, OTM, MMG	1—5,470
Omara et al. [6]	Marcellus	Y		45	DT, MMG	40—25,200
Omara et al. [6]	Uinta	Y		29	DT, OTM, MMG	4—3,580
Goetz et al. [9]	Marcellus	Y		3	DT	4670—8,360
Lan et al. [13]	Barnett	Y		32	MMG	4.5—4,150
Yacovitch et al. [11]	Barnet	Y		7	MMG	23—1,160
Robertson et al. [8]	Fayetteville	Y	Y	50	OTM	21—4,350
Robertson et al. [8]	Uinta	Y	Y	29	OTM	2.2—1,160
Robertson et al. [8]	Denver Julesberg	Y	Y	15	OTM	2.1—326
Robertson et al. [8]	Green River Basin	Y	Y	51	OTM	7.1—3,380

Similarly, Alvarez et al. [4] also extrapolate a sample of site-level studies to produce an estimate of US CH_4 emissions for the production segment. Alvarez rely upon a smaller sample of studies: Brantley et al. [7], Omara et al. [10], Rella et al. [14], and Robertson et al. [8]. The extrapolation approach is also different. Rather than bootstrapping, Alvarez et al. derive probability density functions of emissions assuming a power law relationship between emissions and gas production. The Alvarez et al. model assumes that the underlying emissions distributions are lognormal.

3. Supplementary Methods 3: Comparison of emission and activity factors

An objective of this study, which will be discussed in greater detail in later sections (see Supplementary Methods 6), is the decomposition of emissions into the constituent component-level and equipment-level factors (see **Figure 1**, main text). The purpose of these decompositions is for a comparison with emissions estimates of the GHGI. Throughout this work, we discuss calculation of emissions at the component-level and at the equipment-level. We also make comparisons with various studies conducted at the well-site-scale. These terms are defined as follows:

- Component- The basic constituent parts of equipment which serve either mechanical (e.g., connector) or control (e.g., valve) purposes. A piece of equipment can have 10s to 100s of components.
- Equipment – An assemblage of components fit for a specific purpose of O&NG production, processing, or delivery. For example, a separator is a piece of equipment that separates fluids based on density to allow separate treatment of gases, liquid hydrocarbons, and water.
- Well-site – A well-site (also often referring to as a well-pad, production-site, or production-facility) refers to the set of equipment necessary for O&NG production. This will include one or more wells and ancillary production equipment for handling, separation, and delivery of product to market or a centralized gathering facility. In some cases, a well-site will contain compression equipment in order to meet pressure requirements for delivery of gas off-site.

In both our approach and the approach of the GHGI, total emissions are calculated through two successive extrapolations, first from the component-level to the equipment-level, and second

from the equipment-level to the national-level (**Figure 1**, main text). Extrapolations are performed by multiplying emission factors by activity factors, defined as follows:

- Emission factor (EF) – Average mass of pollutant per unit activity. Activities are often defined as operation of a component for a unit of time, resulting in emission factors with units such as $kgCH_4$ $flange^{-1}d^{-1}$.
- Activity factor (AF) – Usually defined as the number of sources (e.g., counts of equipment or components). However, there are certain cases where emissions are more accurately scaled by a different metric (e.g., horsepower-hours for gas engines).

Therefore, differences between our study and the GHGI can be deduced by comparing emission factors and activity factors at both the equipment-level and component-level. This section serves to accompany the discussion included in the main text. Here, we include tabular comparisons of values with reference to other relevant sections in this supplementary information.

3.1. Component-level factors

Component-level emission factors are averages of component-level emissions survey datasets. Our first comparison is of our component-level emission factors with the data underlying the GHGI [15–17]. As we elaborate in the main text and in Supplementary Methods 5, the GHGI is itself based upon the results of multiple component-level datasets. In **Supplementary Table 2** we compare emission factors reported by these various studies, including API 4589 [15], Star Environmental [16], and the EPA Protocol document [17], with the emission factors generated in this study. For further description of these studies, please refer to Supplementary Methods 6.

We also compare emissions factors reported in 40 CFR part 98, subpart W (referred to as "Subpart W" [3]) for the calculation of equipment leakage in the Greenhouse Gas Reporting Program. In Subpart W, there are three different methods of calculating equipment leakage (referenced in **Supplementary Table 2**). These methods include both "population" emission factors and "leaker" emission factors. Population emission factors (as we describe above) are an average across both emitting components and non-emitting components. Therefore, operators calculate emissions by multiplying component counts at a facility by the population emission factors (referred to as "pop." in **Supplementary Table 2**). In addition to population emission factors, Subpart W also includes two approaches which involve "leaker" emission factors. Leaker

emission factors are an average across only emitting components. In these approaches, operators count total emitting components by either (i) an optical gas imaging approach, or (ii) a Method 21 approach with a leak threshold of 500 ppmv (referred to as “Leaker method (i)” and method “Leaker method (ii)”, respectively, in **Supplementary Table 2**).

Supplementary Table 2: Summary of component-level emission factors. Here, we compare emission factors (units of kgCH_4 component^{-1} day^{-1}) generated in this study with 1990s EPA emission factors, including API 4589 [15], Star Environmental [16], and the EPA Protocol document [17], and emissions factors applied in Subpart W [3]. Note that emission factors for this study are undifferentiated and apply to all product categories (gas, light oil, heavy oil). Population emission factors (Pop.) are calculated as the average across both leaking and non-leaking components, while "leaker" emission factors are an average of only leaking components. In both this study and the EPA Protocol document, "leaker" emission factors are calculated according to a threshold concentration screening value (see Supplementary Methods 4).

		API 1993 (doc. 4589) [15]	Star Environmental [16]		EPA Protocol Document [17]			Subpart W [31]			This study (emission factors undifferentiated across all product categories)	
			Pop.	Leaker	Pop.	Ave. >10,000 ppmv	Pegged > 10,000 ppmv	Pop.	Leaker method (i)[2]	Leaker method (ii)[3]	Pop.	Ave. > 10,000 ppmv
Gas	Valve	0.044	0.007	0.544	0.074	1.616	1.055	0.010	1.782	1.273	0.047	6.205
	Connector	0.002	0.001	0.073	0.003	0.429	0.462	0.001	0.473	0.291	0.006	3.326
	OEL	0.004	0.020	0.116	0.033	0.907	0.495	0.022	1.018	0.691	0.021	2.356
Light oil	Valve	0.005	-	-	0.037	1.434	-	0.018	1.164	0.800	-	-
	Connector	0.001	-	-	0.003	0.429	-	0.003	0.364	0.218	-	-
	OEL	0.010	-	-	0.021	0.725	-	0.018	0.582	0.400	-	-
Heavy oil	Valve	8.6E-05	-	-	1.9E-04	-	-	1.8E-04	1.164	0.800	-	-
	Connector	4.3E-05	-	-	1.7E-04	-	-	1.1E-04	0.364	0.218	-	-
	OEL	4.3E-04	-	-	3.2E-03	4.9E-01	-	2.2E-03	0.582	0.400	-	-

[1]See Tables W-1A for population emissions factors and W-1E for leaker emissions factors in [3]
[2]Leak detection is conducted using an optical gas imaging instrument as specified in Subpart W Section 98.234(a)(1)
[3]Leak detection is conducted using Method 21 as specified in Subpart W Section 98.234(a)(2).

3.2. Equipment-level factors

Equipment-level emission factors are calculated by summing component-level emission factors according to estimated component counts per piece of equipment using methods described in Supplementary Methods 4 for this study and Supplementary Methods 6 for the GHGI. Equipment-level activity factors are applied to extrapolate equipment-level emission factors to estimate total emissions. **Supplementary Table 3** and **Supplementary Table 4** present a comparison of equipment-level emission factors and activity factors between our study and the GHGI for natural gas systems and petroleum systems, respectively. Aggregated emission factors were weighted according to equipment counts. Equipment-level emission factors for this study are separately assigned to marginal wells (<10 mscf/well/day) and non-marginal wells as described in Supplementary Methods 4.

Emission factor probability distributions are presented in **Supplementary Fig. 3**.

Supplementary Table 3: Summary of total emissions and equipment-level emission factors for natural gas systems. Here we compare activity factors, emission factors, and total emissions between this study and the Greenhouse Gas Inventory[1] for natural gas systems. This table presents post hoc emission factors (i.e., averages of emissions randomly assigned by OPGEE's iterative bootstrapping algorithm, see Supplementary Methods 4) for marginal wells (<10 mscf well^{-1} day^{-1}) and non-marginal wells in addition to average emission factors.

	Natural gas systems							
	This study					Greenhouse Gas Inventory (GHGI)		
	Activity factor [# equipment]	Emission factor – Marginal [kg/day]	Emission factor- Non-marginal [kg/day]	Emission factor – Average [kg/day]	Total emissions [Gg]	Activity factor [# equipment]	Emissions factor [kg/day]	Total emissions [Gg]
Well heads	433,430	1.6	4.3	3.4	530.0	419,692	0.3	49.1
Header	-	-	-	-	-	-	-	
Heater	57,248	0.9	2.7	2.0	42.6	54,977	0.7	13.5
Separator	307,816	1.7	4.8	3.7	418.5	298,108	1.1	118.4
Meter	364,058	1.3	3.4	2.7	353.8	351,867	0.6	73.4
Tanks - Leaks	175,843	0.3	1.0	0.7	48.1	-	-	-
Tanks - Unintentional vents	175,843	2.1	11.0	7.9	505.4	-	-	-
Compressor - Recip	35,298	2.5	6.9	5.3	68.7	33,800	5.5	73.2
Dehydrator	12,946	1.8	3.9	3.1	14.8	12,463	1.3	29.0
Chemical Injection Pump	87,681	3.4	7.0	5.7	182.5	74,506	4.2	113.3
Pneumatic Controller	812,370	1.9	3.2	2.8	818.7	833,222	3.5	1072.7
Liquids unloadings	75,850	4.5	11.2	8.8	244.3	73,378	6.0	160.1
Completions[2,3]	3,760	-	-	24.8	34.0	3,760	11.1	29.0
Workovers[2,3]	9,335	-	-	0.7	2.3	9,335	3.5	11.0
Tank - Intentional vents[2]	175,843	0.0	0.2	0.2	15.5	-	-	24.0
Flare methane	13,885	4.3	3.0	3.5	17.6	-	-	17.3
Methane slip	35,298	-	-	7.6	98.3	33,800	21.5	265.0

[1]Other emissions adding to 173 Gg are included in the GHGI but not listed here. This includes produced water tanks, kimray pumps, dehydrator vents, onshore venting, blowdowns and upsets, and pipelines.
[2]Doesn't include contributions from flaring (which are included in the "flare methane" category)
[3]Activity units for completions are counts of events. Thus, the emission factor units are kg $event^{-1} day^{-1}$.

Supplementary Table 4: Summary of total emissions and equipment-level emission factors for petroleum systems. Here we compare activity factors, emission factors, and total emissions between this study and the Greenhouse Gas Inventory[1] for petroleum systems. This table presents post hoc emission factors (i.e., averages of emissions randomly assigned by OPGEE's iterative bootstrapping algorithm, see Supplementary Methods 4) for marginal wells (<10 mscf well^{-1} day^{-1}) and non-marginal wells.

	Petroleum systems							
	This study					Greenhouse Gas Inventory (GHGI)		
	Activity factor [# equipment]	Emissions factor - Marginal [kg/day]	Emissions factor- Non-marginal [kg/day]	Emissions factor – Average [kg/day]	Total emissions [Gg]	Activity factor [# equipment]	Emissions factor [kg/day]	Total emissions [Gg]
Well heads	571,759	0.6	2.8	1.3	277.7	600,519	0.3	65.3
Header	127,739	1.5	8.5	3.9	179.8	138,236	0.2	8.0
Heater	106,289	0.4	2.2	1.0	39.4	114,278	0.4	44.3
Separator	210,930	0.6	3.3	1.5	117.5	215,830	0.2	19.0
Meter	-	-	-	-	-	-	-	-
Tanks - Leaks	466,215	0.3	1.3	0.6	108.3	-	-	-
Tanks - Unintentional vents	466,215	1.1	10.0	4.1	695.5	-	-	-
Compressor - Recip	-	-	-	-	-	-	-	-
Dehydrator	-	-	-	-	-	-	-	-
Chemical Injection Pump	49,239	5.3	5.3	3.8	68.5	57,102	4.2	86.8
Pneumatic Controller	631,851	1.4	3.2	2.0	463.3	623,264	3.5	789.5
Liquids unloadings	-	-	-	-	-	-	-	-
Completions[2,3]	5,124	-	-	36.6	68.5	5,124	12.9	63.5
Workovers[2,3]	-	-	-	0.0	0.0	31,772	1.0	11.7
Tank - Intentional vents[2]	466,215	0.1	18.3	6.2	1060.7	-	-	56.6
Flare methane	34,605	1.6	41.5	14.9	188.6	-	-	83.9
Methane slip	-	-	-	-	-	-	-	93.4

[1]Other emissions adding to 82 Gg are included in the GHGI but not listed here. This include produced water tanks, kimary pumps, dehydrator vents, onshore flaring and venting, blowdowns and upsets, and pipelines.
[2]Doesn't include contributions from flaring (which are included in the "flare methane" category)
[3]Activity units for completions are counts of events. Thus, the emission factor units are kg $event^{-1} day^{-1}$.

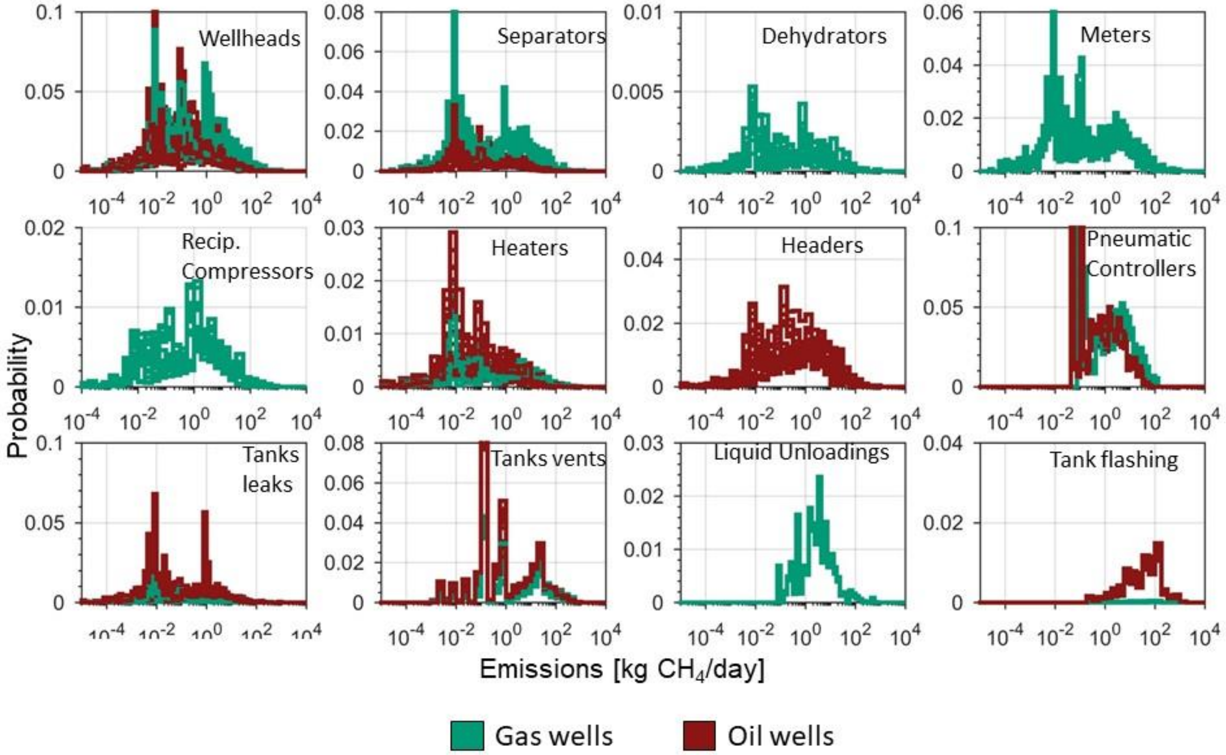

Supplementary Fig. 3: Probability distributions of equipment-level emission factors. Here, we compare gas sites (GOR > 100 mscf/bbl) and oil sites (GOR < 100 mscf/bbl).

3.3. Differences mostly explained by emission factors

Equipment-level activity factors applied in this study are generally very close to those applied in the GHGI. We generated equipment-level activity factors using the same approach as the GHGI (see description of approach using GHGRP data in Supplementary Methods 4), so ideally our values should be identical. Updates to the GHGRP Envirofacts system between when the GHGI generated activity factors and when activity factors were generated for this study leads to small differences (generally < 5%, although a larger adjustment of ~15% in Envirofacts appears to have been made to chemical injection pumps).

On the other hand, wide differences exist with equipment-level emission factors (~1x to 10x for natural gas systems and ~1x to 25x for petroleum systems). This suggests that understanding disagreements between results of this study and the GHGI can be explained through an investigation of equipment-level emission factors and not activity factors.

4. Supplementary Methods 4: The bottom-up CH_4 estimation tool

The analysis platform for this study is the component-level methane subroutine embedded within OPGEE 3.0. This subroutine processes inputs from external databases – specifically equipment-level emissions distributions and well and production values and produces gross emissions estimates.

In this section, we will begin by describing the existing OPGEE 3.0 functionality. Next, we will describe the underlying database of component-level data and how equipment-level emission factors were developed from this data. We will conclude this section by describing equipment-level activity factors.

Below is a flowsheet, **Supplementary Fig. 4**, which illustrates the OPGEE 3.0 CH_4 emissions subroutine.

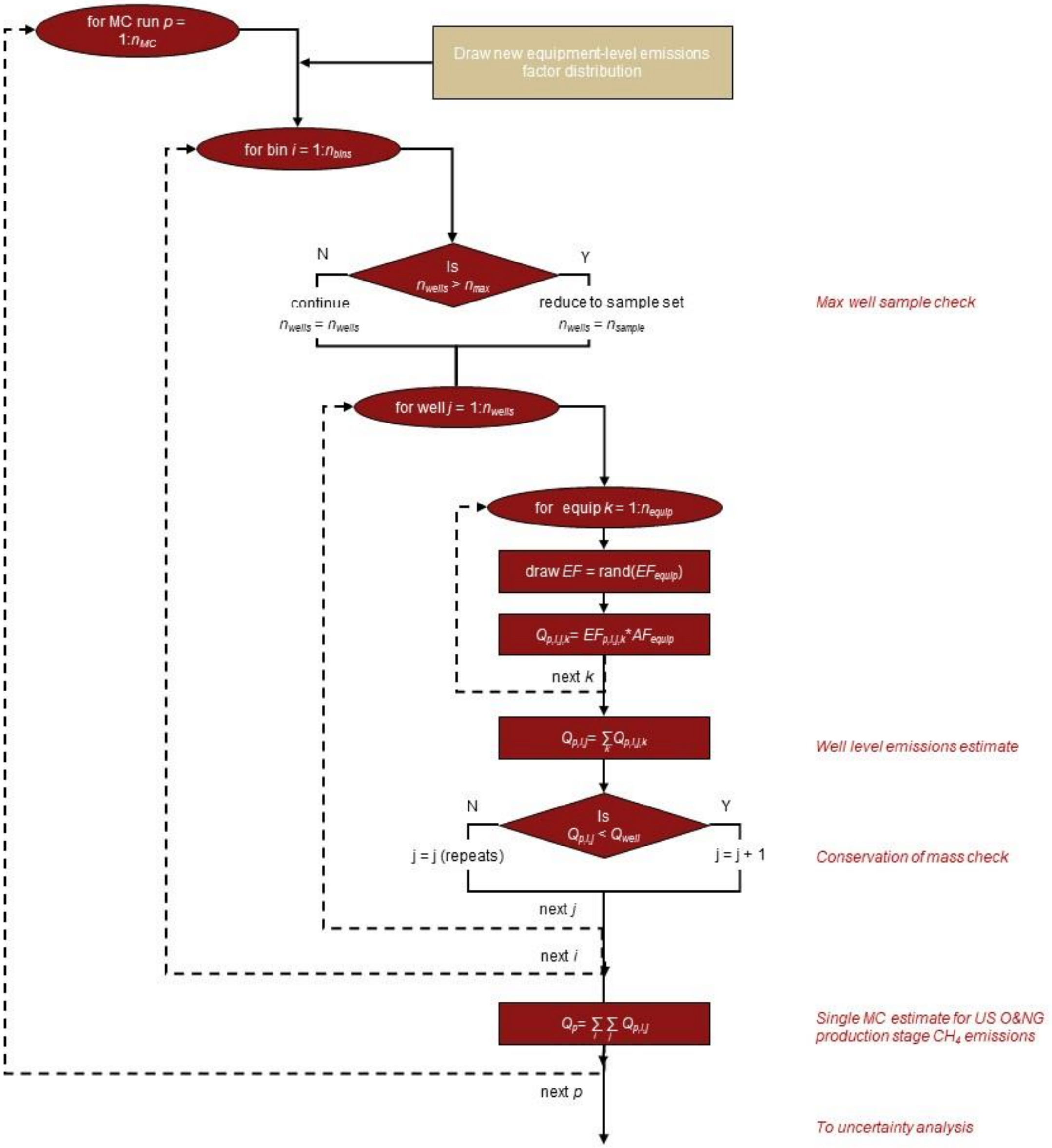

Supplementary Fig. 4: Pseudocode block diagram of OPGEE's CH_4 calculator.

4.1. Structure of the OPGEE component-level methane emissions model

Supplementary Fig. 5 illustrates how the component-level CH_4 emissions subroutine, represented by the simple equation, fits into the broader OPGEE platform. Inputs to the CH_4 emissions subroutine are linked from the Inputs sheet. OPGEE generates outputs (carbon intensity or methane leakage rate) on a field basis, where a field represents an O&NG system with unique

properties. In our case, fields are the productivity tranches/bins, described in Supplementary Methods 5. For each field *i*, emissions are calculated well-by-well. For each well *j*, equipment-level emissions are calculated by multiplying a randomly drawn emissions factor, $EF_{i,j,k}$, by its respective activity factor, af_k. Emissions results from the Calculation table are stored in a separate table by the Fugitives macro, which iterates to the next well. These separate elements of the OPGEE CH_4 emissions subroutine will be described in the following sections.

$$Q_{population} = \sum_{i=1}^{n_{fields}} \left\{ \sum_{j=1}^{n_{wells,i}} \left[\sum_{k=1}^{n_{equip}} EF_{i,j,k} * af_k \right] \right\}$$

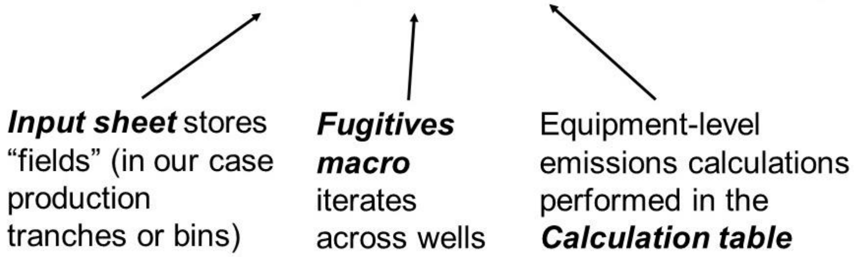

Supplementary Fig. 5: Graphical illustration of CH_4 emissions algorithm

4.1.1. Worksheet

Supplementary Fig. 6 provides an overview of the OPGEE 3.0 component-level CH_4 emissions worksheet. User interaction will typically be limited to the Inputs sheet, Summary table, and Calculations table. The emissions factor distributions and activity factors are accessed by built in functions in the Calculations table. In this section we will describe the Inputs and Calculations table in more detail.

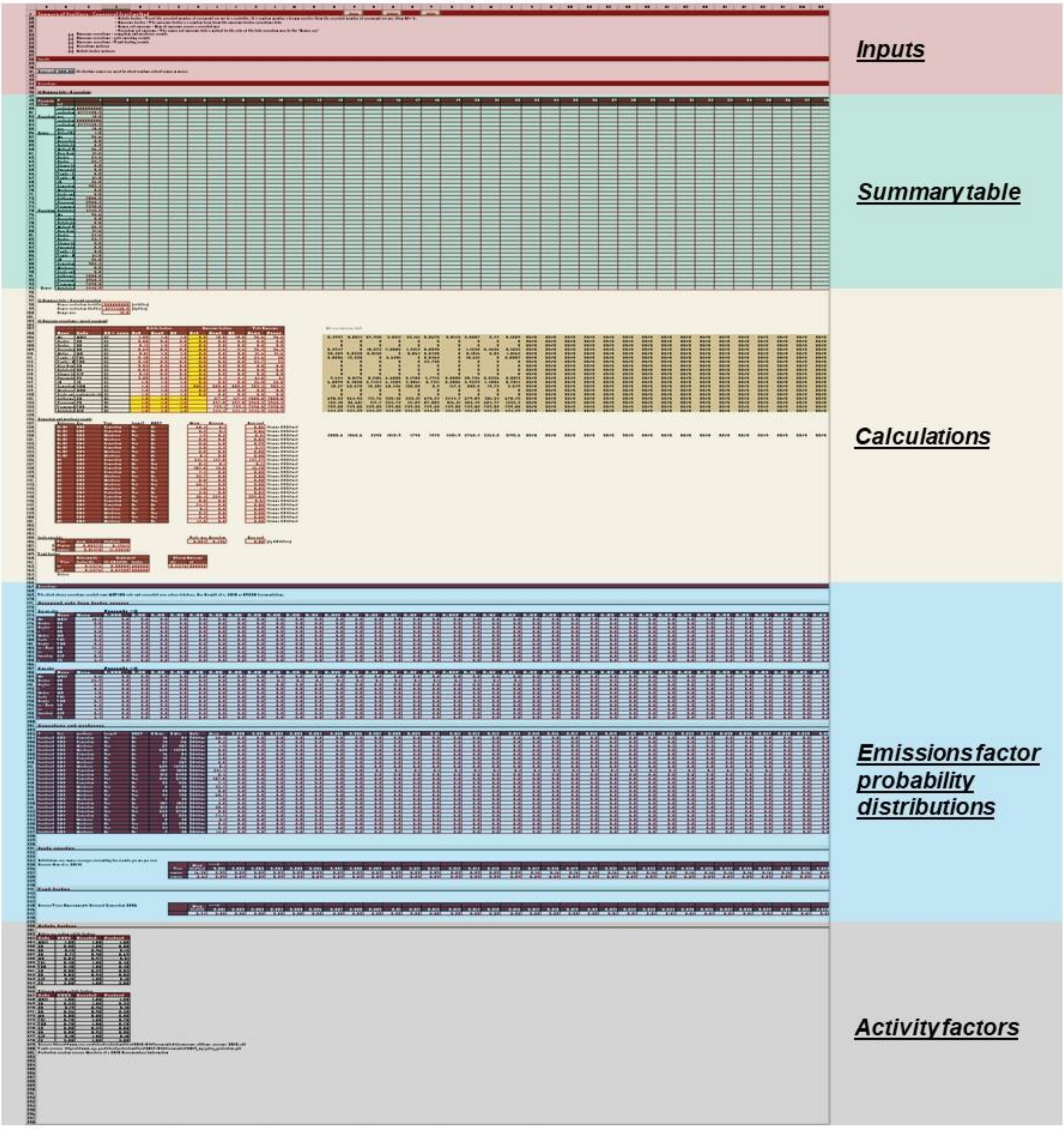

Supplementary Fig. 6: Zoomed-out and labelled view of the Oil Production and Greenhouse gas Emissions Estimator 3.0 component-level CH_4 emissions worksheet

Inputs

In the Inputs section, we include a Sample size parameter, designed to reduce OPGEE processing time. The Sample size parameter sets an upper limit on the number of wells that the OPGEE Fugitives macro will process. If the total number of wells is greater than Sample size, an

extrapolation is performed (more details below). By default, we set the max well sample to be 500 wells. This gives accurate summary statistics and distributions for a tranche/field, but limits the iterations that would be required in modeling a tranche/field with 1000s of wells.

Calculation table

The Calculation table is set up with rows containing all supply chain CH_4 emissions sources and columns containing the emissions calculation parameters (**Supplementary Fig. 7**). Calculation parameters include a methods code (referencing the calculation method applied), emissions factors, activity factors, and total emissions. The methods code is required because the component tool distinguishes between on-site and off-site equipment. This is because in its current iteration the component tool only applies the component-level method to the production segment. Off-site segments (gathering and boosting, transmission, and distribution) apply the site-level method. For these sections, we apply fractional loss rates found in several facility-level studies ([4,18,19]). Calculation of the life cycle emissions is required for the purposes of the mass balance calculation in the macro (see next section).

The total emissions column is divided into sample emissions and population emissions. Sample emissions are calculated by simply summing across all the well realizations performed. However, since the maximum wells calculations per field are limited to 500 (unless adjusted by the user), the total emissions may need to be adjusted. The population emissions are equal to the sample emissions multiplied by the population well count divided by 500 (or user-defined sample size).

			Activity Factors			Emissions Factors			Total Emissions	
Equipment	Code	CL = comp. lvl, SL = site lvl	Det AF	Rand AF	AF	Det EF	Rand EF	EF	Sample	Pop.
Well	WELL	CL								
Header	HD	CL								
Heater	HE	CL								
Separator	SE	CL								
Meter	ME	CL								
Tanks - Leaks	TAL	CL								
Tanks - Vents	TAV	CL								
Recip Compressor	CR	CL								
Dehydrator	DE	CL								
Chemical Injection Pump	CIP	CL								
Pneumatic Controller	PC	CL								
LU	LU	CL								
Completions	COM	CL								
Workovers	WOR	CL								
Storage tank venting	TK	CL								
Gathering and Boosting	GB	SL								
Processing	PR	SL								
Transmission	TRA	SL								
Distribution	DIS	SL								

$$af \cdot EF = Q$$

Supplementary Fig. 7: Screenshot of the *Calculation table*. Here, shaded columns correspond to activity factors (af), emissions factors (EF), and total emissions (Q).

4.1.2. Fugitives macro

The Fugitives macro serves several functions in the OPGEE component-level CH_4 emissions tool. First, it allows OPGEE to iterate across wells, and second it executes two conditional statements. These conditional statements are the well sampling (already described above) and a conservation of mass check.

The first conditional statement executed in the Fugitives macro is to check if well sampling is required. If $n_{wells} > n_{max}$ for a field (where n_{max} is the maximum number of wells OPGEE will process), then a smaller sample set where $n_{wells} = n_{max}$ is created. The sample sets are tracked in the Summary table and before CH_4 emissions are passed to other sub-modules, emissions are extrapolated from n_{max} to n_{wells}.

The second conditional statement is a conservation of mass (COM) check. At every well, a check is performed to ensure COM is not violated. In the COM check, the summed leaks are compared to total gas production at the well-pad. By applying the COM check we ensure total CH_4 emissions do not exceed 100% of gas (CH_4) productivity (also accounting for the gathering and boosting, processing, transmission, and distribution sectors). The only emissions source that is

not included in the COM is completions and workovers since these emissions are infrequent and do not occur while the well is producing. If emissions exceed well-level CH_4 production, the macro loops back and iterates again for that well.

The COM check wouldn't be required if quantified emissions measurements from the literature were coupled with information on the gas throughput of the component. However, in contrast to the site-level studies synthesized by Omara et al [20], few (if any) component-level measurement studies provide information on the gas production volumes of measured wells and well-pad equipment. That is, these studies typically sample and quantify CH_4 emissions volumes from different pieces of equipment. Due to the missing volumetric production data, we only have CH_4 emissions volumes in absolute terms (e.g., scf per hour or kg per day), not in percentage loss terms. The application of COM to the iterative draws is what restricts high emitters at low productivity well-pads and is part of what leads to the scale dependence of fractional CH_4 emissions rates.

4.1.3. Format of OPGEE outputs

Outputs from OPGEE are of the following form:

	Tranche # [1-74]	OPGEE Column # [200-273]	Fraction sampled [Actual well count Sample size^{-1}]	Well productivity [kg well^{-1} day^{-1}]	Well productivity [scf well^{-1} day^{-1}]	Emissions array[1]
1						
2						
3						
…						
…						
n_{wells}						

[1]There are 16 columns and the order is as follows: wellheads, headers, heaters, separators, meters, tank leaks, unintentional tank vents, reciprocating compressors, chemical injection pumps, pneumatic controllers, liquid unloadings, completions, workovers, intentional tank flashing events, and flare methane.

Note that every row here is on a well-basis (i.e., one row per well). Also note that there are many zero values in the results spreadsheets and this is due to a number of different reasons. First, as

we describe later, equipment are randomly assigned to wells on the basis of activity-drivers (number of equipment X per well, for example ~80% of natural gas system wells will be assigned a separator, or ~40% for petroleum systems). Second, based on our equipment-level emission factor distributions there are certain cases where all components on a piece of equipment register no leaks. Therefore, equipment-level emission factor distributions will contain some zero values.

4.2. Database of component-level studies

The bottom-up approach begins with a database of component-level survey data. We begin this section by describing the criteria for screening component-level data and our correspondence structure for organizing data across various studies with differing definitions. We conclude by summarizing our data for component counts, component-level emission factors, and fraction of components leaking.

4.2.1. Summary of criteria for inclusion in our analysis

We reviewed the literature for field studies with measurement and quantification of CH_4 emissions data at the component scale. All studies with emission measurement performed at the wellsite/facility or producing field/region scale were removed from consideration. To the extent possible, we only include studies with component-level direct measurements (e.g., using a High Flow Sampler) and avoid studies with simulated emissions data (e.g., based on engineering equations). After confirming the correct scale, the document was included if it contained any of the following information: (i) activity counts for numbers of components per piece of equipment or per site, (ii) data on fraction of components emitting in a survey, or (iii) data on quantified emission volumes per emitting component or source.

Next, because we are only modeling equipment emissions in the production-segment, we exclude studies which focus solely on the midstream or downstream segments. Further, though a number of high quality measurement campaigns have been conducted in Canada (e.g., [21,22]), in this study we only include U.S.-based datasets in our analysis of U.S. emissions. This is to avoid possible biases in the dataset due to differing regulatory and operating standards between countries. Future work will compare Canadian and US results. All studies passing this final filter are

summarized in **Table 2** (Main text). Due to severe data limitations, note that we make an exception to our filters and apply Canadian data from Clearstone [22] in component counts and fraction of components emitting (for regulators only). No Canadian datasets are used for emissions data. We would expect component counts per equipment to be to a large extent standardized between US and Canada.

4.2.2. Correspondence of each study into standard classification scheme for equipment and components

As noted in the main text, component-level activity and emissions rate data are aggregated from various studies. In order to aggregate the data from the various studies, a standardized set of components is required.

First, a 16-fold categorization structure was initially applied in our database:

1. Threaded connections
2. Valve
3. Open-ended line
4. Pressure-relief valve
5. Compressor seal
6. Regulator
7. Flanges
8. Vents
9. Pumps
10. Tank vents
11. Tank thief hatch
12. Tank pressure-relief valve
13. Pneumatic controller/actuator
14. Chemical injection pump
15. Other
16. Not recorded

Supplementary Table 5 provides counts and average component-level emission factors across studies for this 16-fold classification scheme.

Supplementary Table 5: Quantified measurement count and emissions factor (leaker) by study (16-fold scheme).

		Threaded Conns.	Valve	Open-ended line	Pressure-relief valve	Comp. seal	Regulator	Flanges	Vents	Pump	Pneumatic controller/ actuator	Tank vent	Tank hatch/ hole	Tank PRV	Chemical Injection Pump	Other	Not recorded
		TC	VL	OEL	PRV	CS	REG	F	VT	PM	PC	TV	TH	TP	CIP	OTH	NR
API 1993																	
Counts	500 - 10000 ppmv	9	16	16	5	1	-	-	0	-	-	-	-	-	-	2	-
	> 10000 ppmv	29	97	38	5	1	-	-	4	-	-	-	-	-	-	28	-
	All	38	113	54	10	2	-	-	4	-	-	-	-	-	-	30	-
Emissions factors [kg d^{-1}]	500 - 10000 ppmv	0.02	0.02	0.01	0.00	0.02	-	-	-	-	-	-	-	-	-	0.01	-
	> 10000 ppmv	0.30	0.44	0.16	1.70	0.77	-	-	0.22	-	-	-	-	-	-	0.35	-
	All	0.23	0.38	0.12	0.85	0.40	-	-	0.22	-	-	-	-	-	-	0.32	-
Allen 2013																	
Counts	500 - 10000 ppmv	-	-	-	-	-	-	-	-	-	-	-	-	-	-	-	-
	> 10000 ppmv	-	-	-	-	-	-	-	-	-	-	-	-	-	-	-	-
	All	44	41	5	3	-	51	6	53	-	305	124	-	-	62	74	-
Emissions factors [kg d^{-1}]	500 - 10000 ppmv	-	-	-	-	-	-	-	-	-	-	-	-	-	-	-	-
	> 10000 ppmv	-	-	-	-	-	-	-	-	-	-	-	-	-	-	-	-
	All	1.22	2.59	0.76	2.84	-	1.35	1.22	8.65	-	4.84	3.52	-	-	5.31	1.58	-
Allen 2014a																	
Counts	500 - 10000 ppmv	-	-	-	-	-	-	-	-	-	-	-	-	-	-	-	-
	> 10000 ppmv	-	-	-	-	-	-	-	-	-	-	-	-	-	-	-	-
	All	-	-	-	-	-	-	-	-	-	377	-	-	-	-	-	-
Emissions factors [kg d^{-1}]	500 - 10000 ppmv	-	-	-	-	-	-	-	-	-	-	-	-	-	-	-	-
	> 10000 ppmv	-	-	-	-	-	-	-	-	-	-	-	-	-	-	-	-
	All	-	-	-	-	-	-	-	-	-	2.26	-	-	-	-	-	-
Bell 2017																	
Counts	500 - 10000 ppmv	-	-	-	-	-	-	-	-	-	-	-	-	-	-	-	-
	> 10000 ppmv	-	-	-	-	-	-	-	-	-	-	-	-	-	-	-	-

	All	18	9	-	14	-	18	3	3	-	41	35	-	-	3	82	20
Emissions factors [kg d^{-1}]	500 - 10000 ppmv	-	-	-	-	-	-	-	-	-	-	-	-	-	-	-	-
	> 10000 ppmv	-	-	-	-	-	-	-	-	-	-	-	-	-	-	-	-
	All	2.71	1.00	-	2.57	-	6.08	2.49	5.61	-	2.45	23.84	-	-	0.23	4.03	1.95
ERG 2011																	
Counts	500 - 10000 ppmv	139	45	0	-	-	20	3	4	-	1	1	46	1	0	55	11
	> 10000 ppmv	339	94	13	-	-	75	0	57	-	6	33	219	40	1	708	37
	All	478	139	13	-	-	95	3	61	-	7	34	265	41	1	763	48
Emissions factors [kg d^{-1}]	500 - 10000 ppmv	0.74	3.33	-	-	-	2.18	0.13	0.01	-	0.05	0.13	2.92	0.09	-	3.33	0.01
	> 10000 ppmv	4.15	14.85	9.73	-	-	6.50	-	9.90	-	16.57	44.19	51.80	60.30	127.87	15.01	0.18
	All	3.16	11.12	9.73	-	-	5.59	0.13	9.25	-	14.21	42.89	43.31	58.83	127.87	11.20	0.14
Thoma 2017																	
Counts	500 - 10000 ppmv	0	0	0	0	0	0	0	0	0	0	0	0	0	0	0	0
	> 10000 ppmv	0	0	0	0	0	0	0	0	0	80	0	0	0	0	0	0
	All	0	0	0	0	0	0	0	0	0	80	0	0	0	0	0	0
Emissions factors [kg d^{-1}]	500 - 10000 ppmv	-	-	-	-	-	-	-	-	-	-	-	-	-	-	-	-
	> 10000 ppmv	-	-	-	-	-	-	-	-	-	0.15	-	-	-	-	-	-
	All	-	-	-	-	-	-	-	-	-	0.15	-	-	-	-	-	-
Pacsi 2019																	
Counts	500 - 10000 ppmv	69	14	5	1	-	9	1	0	-	-	-	-	-	-	6	-
	> 10000 ppmv	44	18	2	2	-	9	2	1	-	-	-	-	-	-	9	-
	All	113	32	7	3	-	18	3	1	-	-	-	-	-	-	15	-
Emissions factors [kg d^{-1}]	500 - 10000 ppmv	0.59	1.58	3.56	0.94	-	0.24	0.07	-	-	-	-	-	-	-	0.28	-
	> 10000 ppmv	1.78	2.97	0.09	0.21	-	1.20	1.06	14.39	-	-	-	-	-	-	0.96	-
	All	1.05	2.36	2.57	0.46	-	0.72	0.73	14.39	-	-	-	-	-	-	0.69	-

Modifications were made to this 16-fold categorization structure prior to consolidating measurements for the bootstrapping exercise: (i) Connectors and flanges were combined into a single category, and (ii) the categories “pump”, “vent”, and “not recorded” were removed due to lack of activity data. Regarding vents specifically, we should note that many emissions classified as vents (e.g., pneumatic instruments, storage tanks open to the atmosphere, liquids unloading) have been already included explicitly in other categories. Although some have not (e.g., glycol dehydrator off-gassing, equipment blowdowns) by not including the generic category “vents” we avoid potential double counting. Similarly, it is unclear if the generic label “pump” is in reference to chemical injection pumps.

With these adjustments, our proposed component structure is therefore a 12-fold categorization scheme. Thus, all components from all studies will be grouped into 11 categories and “other” as follows:

1. Threaded connections and flanges
2. Valve
3. Open-ended line
4. Pressure-relief valve
5. Compressor seal
6. Regulator
7. Pneumatic controller/actuator
8. Chemical injection pump
9. Tank vents
10. Tank thief hatch or exterior hole
11. Tank pressure-relief valve
12. Other

We generated the 12-fold categorization scheme by examining the frequency of data reporting and common levels of data granularity across the studies. The range of details provided varies by study, so using too fine or too coarse of a classification scheme would be sub-optimal. If too fine of a classification scheme is used, data become challenging to use from studies where only

coarse categorization is reported. If too coarse of a classification scheme is used, large amounts of possibly useful identifying information are lost.

Note that although “compressor seal” is included in this list, it is not included in emission factor summary tables. This is because although compressor seals are included in numerous studies in our (unfiltered, including non-USA and midstream/downstream measurements) database and in activity factor matrices (component counts per equipment), in our final, filtered emissions dataset no emissions measurements existed for compressor seals.

There is always a tradeoff in the generality versus the simplicity of a naming scheme. A simple naming scheme is easier to record data into, and perhaps more generalizable between studies. However, a simple naming scheme removes nuance (e.g., what kind of compressor seal is used?). We believe that the above 12-fold level of aggregation strikes the correct balance and allows most datasets in the literature to be used.

A standardized structure was also required for equipment categories. We generate a proposed 11-fold categorization scheme is as follows:

1. Well
2. Header
3. Heater
4. Separator
5. Meter
6. Tanks – leaks
7. Tanks – vents
8. Compressor – reciprocating
9. Dehydrator
10. Chemical injection pump
11. Pneumatic controller/actuator

Because tanks are an important emissions source, two separate equipment bins were created for tanks. The “Tanks – leaks” category tracks all non-vent/hatch emissions on a tank (e.g., connectors) while the “Tank – vent” category contains “Tank vents”, “Tank thief hatch or exterior hole”, and “Tank pressure-relief valve” component categories.

4.2.3. Component counts

Data on component counts are relatively sparse and are sourced in this study from the American Petroleum Institute [16], Pacsi et al [23], and Clearstone [22]. Note that we make an exception to our filters here and apply Canadian data from the Clearstone study due to the severe data limitations inherent in only including 2 U.S. studies.

Note that there is a high degree of uncertainty in our assumptions regarding counts of thief hatches, pressure-relief valves, and open vents at tanks. Here we assume one thief hatch and PRV per tank, and zero vents as these are already accounted for with purposeful venting. See also Supplementary Methods 5, where we adjust the fraction of components emitting variable downwards for the pressure-relief valves to account for the possibility of overlap or double counting between pressure relief valves and purposeful venting.

Similarly to the handling of quantified leakage data, we transform the study component counts into our standardized classification scheme by using correspondence matrices (**Supplementary Table 6** and **Supplementary Table 7**). Component counts are classified for oil and gas systems using naming conventions in studies (e.g., API and Clearstone classify oil and gas systems separately, while Pacsi et al [23] provide aggregate component counts).

Supplementary Table 6: Component counts for natural gas system equipment. This table describes the total range of values found in the literature (lower bound – upper bound).

	Threaded connections/ flange	Valve	Open-ended line	Pressure-relief valve	Compressor seal	Regulator	Pneumatic controller	Chemical Inj Pump	Tank - hatch/hole	Tank-PRV	Tank-vent	Other
Well	69-298	12-23	1-3	1-4	0	1-1	0	0	0	0	0	1-1
Heater	20-117	2-19	1-3	1-1	0-0	2-4	0	0	0	0	0	1-1
Separator	62-180	20-29	1-5	1-2	0	3-3	0	0	0	0	0	2-2
Meter	39-100	3-19	1-2	1-2	0	2-2	0	0	0	0	0	2-2
Tank	3-54	1-7	1-2	1-1	0-0	1-1	0	0	1	1	0	0
Compressor – Reciprocating	226-586	18-35	1-5	2-4	2-4	1-6	0	0	0	0	0	1-1
Dehydrator	27-217	6-29	1-5	1-3	1-1	6-6	0	0	0	0	0	2-2
Chemical Inj. Pump	0	0	0	0	0	0	0	1	0	0	0	0
Pneum. Cont.	0	0	0	0	0	0	1	0	0	0	0	0

Supplementary Table 7: Component counts for petroleum system equipment. This table describes the total range of values found in the literature (lower bound – upper bound).

	Threaded connections/ flange	Valve	Open-ended line	Pressure-relief valve	Compressor seal	Regulator	Pneumatic controller	Chemical Inj Pump	Tank - hatch/hole	Tank-PRV	Tank-vent	Other
Well	61-298	7-23	1-3	1-4	0	0	0	0	0	0	0	0
Header	23-520	2-143	1-4	1-1	0	0	0	0	0	0	0	1-1
Separator	62-139	11-28	2-3	1-1	0	0	0	0	0	0	0	1-1
Tank	6-76	2-15	1-3	1-1	1-1	1-1	0	0	1	1	0	0
Chemical Inj. Pump	0	0	0	0	0	0	0	1	0	0	0	0
Pneum. Cont.	0	0	0	0	0	0	1	0	0	0	0	0

4.2.4. *Component level emissions factors and fraction of components emitting*

Due to differences in the detection threshold of screening technologies used in field campaigns, different studies will sample different parts of the true population emissions distribution. In addition to differences in screening, not all studies perform a full and comprehensive component count and survey across all well sites. Therefore, care must be taken in combining studies into a singular database to ensure that (i) certain parts of the distribution are not over or under-represented in our dataset, and that (ii) fraction of components emitting (the ratio of leaking components to all components counted) is consistent with our dataset's emissions distribution. To ensure that we are not over or under-sampling a subset of the true distribution, we separated our dataset at 10,000 ppmv. Fraction of components emitting is calculated independently for the two sub-populations.

For example, if the fraction of components emitting is defined as fraction of emitting component X divided by total count of component X surveyed we must be careful how we define a leaker. The definition of leaker is different depending on which screening technology was used. The EPA Method 21 procedure involves screening components with a toxic vapor analyzer, a hydrocarbon detection probe with a range extending from approximately 0.5-50,000 ppmv. Optical gas imaging (OGI) is an EPA approved alternative to Method 21 which involves viewing components from a distance with an infrared camera. OGI is less labor intensive compared to Method 21 but is also less precise (detection floor approximately 10,000 ppmv). For example, in the API 4589 [15] dataset, which applied Method 21, leaks as low as 10 ppmv are included, whereas in Allen et al [24] surveying is done using only an OGI camera, which is very unlikely to see a leak resulting in 10 ppm local enhancement.

Method 21 screening values (in concentration, ppmv) offer a means of ensuring consistency across sampled emissions measurements and fraction of components emitting. Further, for all studies that provide sufficient data to quantify fraction of components emitting, concentration data are available. Given that 10,000 ppmv is the concentration requirement for OGI devices in Subpart OOOOa [2], we choose this as a cutoff for measurement data and fraction of components emitting. We note, however, that this is somewhat imprecise given that the threshold for detecting an emitter with an infrared camera is not fixed at an inherent threshold of 10,000 ppmv but also depends on operator experience and site-specific variables such visual background and

wind speeds. Although OGI is operationally less precise than Method 21, we expect the leakage distribution to be more balanced above 10,000 ppmv.

Our dataset is split as follows:

(i) Dataset ≥ 10,000 ppmv: This set includes data with Method 21 measurements ≥ 10,000 ppmv (API 4589 [15], ERG [12], Pacsi et al. [23]) but also primarily OGI-based measurement campaigns (Allen et al. [24], Bell et al. [25]). We note that this classification isn't perfect given that Bell et al. [25] also used a toxic vapor analyzer and therefore their data will include some measured emitters > 500 ppmv and < 10,000 ppmv. However, we could not differentiate these emitters given that no concentration values were provided along with each emitter determination.

(ii) Dataset 500-10,000 ppmv: For this set, we were only able to include portions of datasets based on studies that provided Method 21 concentration measurements (API 4589 [15], ERG [12], Pacsi et al. [23]). We note that 5 measurements in ERG labelled "M21&OGI" which registered < 500 ppmv are included in this set. However, this may have been a mistake given that ERG notes only measurements screening >500 ppmv were sampled with the High Flow sampler.

Supplementary Table 8 and **Supplementary Table 9** contain summary statistics for our dataset by component and by study, respectively. Note that the measurement counts in **Supplementary Table 8** (n = 3546) don't add up directly to the measurement counts in **Supplementary Table 9** (n = 3744) given that 198 measurements did not have component labels.

Supplementary Table 8: Consolidated component-level emission factors (12-fold scheme). Emission factors are also disaggregated according to ≥10,000 ppmv and < 10,000 ppmv screening measurements.

Component	N [samples]	Minimum [kg CH4/d]	Maximum [kg CH4/d]	Mean [kg CH4/d]	Median [kg CH4/d]	Contr of SE [top 5%]
>10,000 ppmv						
Comp. Seal	1	7.71E-01	0.77	0.77	0.77	100%
OEL	58	0.00E+00	60.71	2.36	0.04	72%
Other	901	0.00E+00	439.82	11.64	3.32	72%
Pneu. controller	809	0.00E+00	66.15	3.14	0.30	47%
PRV	24	0.00E+00	12.20	2.23	0.55	45%
Regulator	153	0.00E+00	79.64	4.42	1.35	42%
Connectors	485	0.00E+00	210.86	3.33	0.26	62%
Valve	259	0.00E+00	708.65	6.20	0.26	68%
Chemical inj. pumps	66	5.70E-02	127.87	6.94	1.35	53%
Tank hatch/hole	219	0.00E+00	510.51	51.80	21.34	36%
Tank PRV	40	1.30E-01	351.53	60.30	20.59	24%
Tank vent	68	4.89E-02	288.65	33.71	11.88	36%
All	3082	0.00E+00	708.65	10.91	1.23	57%
500 - 10,000 ppmv						
Comp. Seal	1	2.23E-02	0.02	0.02	0.02	100%
OEL	21	0.00E+00	17.75	0.85	0.00	99%
Other	63	0.00E+00	14.48	0.47	0.01	99%
Pneu. controller	1	5.06E-02	0.05	0.05	0.05	100%
PRV	6	0.00E+00	0.94	0.16	0.00	99%
Regulator	29	2.49E-03	16.50	1.57	0.01	68%
Connectors	221	0.00E+00	60.48	0.65	0.01	88%
Valve	75	0.00E+00	126.72	2.29	0.01	96%
Chemical inj. pumps	1	0.00E+00	0.00	0.00	0.00	0%
Tank hatch/hole	46	0.00E+00	36.49	2.92	0.71	55%
Tank PRV	1	9.32E-02	0.09	0.09	0.09	100%
Tank vent	1	1.30E-01	0.13	0.13	0.13	100%
All	464	0.00E+00	126.72	1.17	0.01	84%

Supplementary Table 9: Consolidated component-level emission factors according to study. Emission factors are also disaggregated according to ≥10,000 ppmv and < 10,000 ppmv screening measurements.

Study	N [samples]	Minimum [kg CH4/d]	Maximum [kg CH4/d]	Mean [kg CH4/d]	Median [kg CH4/d]	Contr of SE [top 5%]
			>10,000 ppmv			
API1993	202	0.000	8.77	0.38	0.00	74%
Allen2013	645	0.000	132.97	4.08	1.24	42%
Allen2014a	377	0.000	66.15	2.26	0.00	58%
Bell2017	246	0.049	212.14	6.23	1.11	63%
ERG2011	1623	0.000	708.65	18.15	4.02	50%
Thoma 2017	80	0.039	1.99	0.15	0.04	47%
Pasci 2019	87	0.007	32.57	1.93	0.25	54%
			500-10,000 ppmv			
API 1993	49	0.000	0.10	0.01	0.00	42%
Allen2013	1	0.000	0.00	0.00	0.00	0%
Allen2014a	1	0.000	0.00	0.00	0.00	0%
Bell2017	1	0.000	0.00	0.00	0.00	0%
ERG2011	326	0.000	126.72	1.41	0.01	85%
Thoma 2017	1	0.000	0.00	0.00	0.00	0%
Pasci 2019	105	0.002	17.75	0.81	0.08	75%

Next, values for fraction of components emitting were estimated as follows (**Supplementary Table 10**).

- For all studies which provide both comprehensive component counts and concentration ppmv) screening data (API 4589 [15], ERG [12], Pacsi et al. [23]), counts of screening measurements were used to estimate fraction of components emitting.
- ERG note that only one in ten components were screened using Method 21. Therefore, in calculating fraction of components emitting, the emitter count < 10,000 ppmv was multiplied by ten.
- None of these studies accounted for regulators, therefore a fraction of components emitting value for regulators was taken from a Canadian study by Clearstone [22].

Pneumatically operated devices (controllers and chemical injection pumps, defined in Supplementary Methods 1) are treated differently from other components. For other components, as we described in the previous section, an OGI or Method 21 campaign was conducted and equipment leaks were quantified if a leak was detected. In contrast, given natural gas driven

pneumatic devices vent gas as a part of normal operation, campaigns targeting these devices (e.g., Allen et al. [24]) measured all controllers and pumps located on sites. Therefore, count of components quantified divided by total component counts equals one (i.e., we assign a fraction of components emitting value of one) for pneumatic controllers and chemical injection pumps. Due to the fact that pneumatic controllers actuate intermittently, Allen et al. [26] note that their dataset "may be influenced by the estimated emission rate for devices that had no emissions over the 15 min sampling period". This concern regarding uncertainty introduced by intermittency has been noted recently by Luck et al. [27], who measured a smaller sample of pneumatic devices (72 devices) but for much longer periods (average of 76 hours, versus 15 minutes in Allen et al. [26]). Luck et al. caution the high degree of uncertainty in using short duration measurements to characterize emissions from pneumatic controllers and, especially for intermittent venting, "there is a low probability that the actuations occurring during that sample will be representative of the actuation rate over an extended period" [27]. Further the Allen et al. [26] emission data includes zero emission measurements for emergency shutdown devices (ESD) which rarely actuate. It is unclear if ESDs are reported to the GHGRP (which constitutes our activity data). For these reasons, our emissions estimate for pneumatic devices could be an underestimate.

Supplementary Table 10: Review of studies surveyed in developing fraction of components emitting values

	Threaded connections/ flange	Valve	Open-ended line	Pressure-relief valve	Compressor seal	Regulator	Pneumatic controller	Chemical Inj Pump	Tank - hatch/hole	Tank-PRV	Tank-vent	Other
						API 1993						
Component counts	81,659	17,649	2,187	420	62	-	-	33	-	-	-	670
Emitter Count												
500 - 10,000 ppmv	566	440	55	13	2	-	-	1	-	-	-	102
> 10,000 ppmv	237	247	29	1	4	-	-	-	-	-	-	41
Fraction emitting												
500 - 10,000 ppmv	0.69%	2.49%	2.51%	3.10%	3.23%	-	-	3.03%	-	-	-	15.22%
> 10,000 ppmv	0.29%	1.40%	1.33%	0.24%	6.45%	-	-	0.00%	-	-	-	6.12%
						ERG 2011						
Component counts	603,026	80,465	-	-	-	-	-	-	1,163	1,163	1,163	-
Emitter Count												
500 - 10,000 ppmv	1,390	450	-	-	-	-	-	-	480	20	10	-
> 10,000 ppmv	339	94	-	-	-	-	-	-	292	73	33	-
Fraction emitting												
500 - 10,000 ppmv	0.23%	0.56%	-	-	-	-	-	-	39.55%	0.86%	0.86%	-
> 10,000 ppmv	0.06%	0.12%	-	-	-	-	-	-	18.83%	3.44%	2.84%	-
						Pacsi 2019						
Component counts	46,442	7,396	414	366	-	-	-	-	-	-	-	-
Emitter Count												
500 - 10,000 ppmv	70	14	5	1	-	-	-	-	-	-	-	-
> 10,000 ppmv	46	18	2	2	-	-	-	-	-	-	-	-
Fraction emitting												
500 - 10,000 ppmv	0.15%	0.19%	1.21%	0.27%	-	-	-	-	-	-	-	-
> 10,000 ppmv	0.10%	0.24%	0.48%	0.55%	-	-	-	-	-	-	-	-

Supplementary Table 11: Lower and upper limits of uniform distribution of fraction of components emitting values.

	Threaded connections/ flange	Valve	Open-ended line	Pressure-relief valve	Compressor seal	Regulator	Pneumatic controller	Chemical Inj Pump	Tank - hatch/hole	Tank-PRV	Tank-vent	Other
> 10,000 ppmv												
Lower bound	0.06%	0.12%	0.48%	0.24%	6.45%	0.88%	100%	100%	18.83%	0.00%	0.00%	6.12%
Upper bound	0.29%	1.40%	1.33%	0.55%	6.45%	0.88%	100%	100%	18.83%	3.44%	2.84%	6.12%
500-10,000 ppmv												
Lower bound	0.15%	0.19%	1.21%	0.27%	3.23%	0.88%	100%	100%	39.55%	0.00%	0.00%	15.22%
Upper bound	0.69%	2.49%	2.51%	3.10%	3.23%	0.88%	100%	100%	39.55%	0.86%	0.86%	15.22%

4.2.5. *Additional information on correspondence matrices*

For each study we need to align the categories of components used by the authors of a study to our common component definitions that can be used across all studies. In order to do this we create a correspondence matrix **P** that converts the original data categorization scheme into the new data categorization. This correspondence matrix is composed using the following procedure:

Assume that we have a component count matrix **C** for a set of *m* equipment types and *n* component types, organized as follows for a given study:

	Component 1	Component 2	…	Component n
Equipment 1	c_{11}	c_{12}	…	c_{1n}
Equipment 2	c_{21}	c_{22}	…	c_{2n}
…	…	…	…	…
Equipment m	c_{m1}	c_{m2}	…	c_{mn}

For a given equipment type *i* ($i \in 1 \ldots m$) and component type *j* ($j \in 1 \ldots n$), the value of c_{ij} is the number of components of that type present in that equipment type.

However, each set of equipment and component types can vary between studies. That is, the values of *m* and *n* can differ (i.e., how many types of equipment or how many types of components) between studies. Also, the names of components and equipment can differ, as can their order of presentation between studies.

Given any given component count matrix **C** in the form presented for a given study, we can create a standardization matrix **P** that will permute and change the shape of C such that it has the same set of components in a standard list of components $\mathbf{c}^* = [c_1^*, c_2^*, c_3^* \ldots c_n^*]$. We define the matrix P as follows:

- An entry of **P** at location $p_{ij} = 1$ if component type i in the study dataset should be included as part of component type j in the standardized component list

- An entry of **P** at location $p_{ij} = 0$ if component type i in the study dataset should not be included as part of component type j in the standardized component list

We show a hypothetical example below. In this example, the hypothetical study dataset has two types of compressors -- reciprocating compressors and centrifugal compressors. The study also contains four possible types of components at each compressor: (1) centrifugal compressor seals, (2) reciprocating compressor seals, (3) threaded connections, and (4) flanges. However, in our standardized component set, we want to group compressor seals all together in a group called "CS" and also group threaded connections and flanges into a category called "connectors". If we have the following raw data **C** from the study:

	Centr. CS	Recip. CS	Threaded connectors	Flanges
Centrifugal compressor	6	0	6	7
Reciprocating compressor	0	7	37	14

We can create a standardization matrix **P** as follows:

	CS	Connectors
Centr. CS	1	0
Recip. CS	1	0
Threaded connectors	0	1
Flanges	0	1

Note that the standardization matrix has dimension related to the dataset of interest: our starting set of components in the rows and our desired set of components is in the columns. We can then create a new dataset **F** with the component counts presented for each type:

$$\mathbf{F} = \mathbf{C} \cdot \mathbf{P} \qquad (1)$$

Or, in our example, **F** is equal to:

	CS	Connectors

Centrifugal compressor	6	13
Reciprocating compressor	7	51

The benefit of this approach is that the correspondence between the original data set and the final dataset is uniquely defined by **P**. We do not reproduce the correspondence matrices **P** here, but they are available in the Github database.

4.3. Development of equipment-level emission factors

4.3.1. The stochastic failure approach

The stochastic failure approach is used to estimate emissions from all equipment leakage emissions and unintentional process vents from tanks. In the stochastic failure approach, a sample of component-level measurements are iteratively re-sampled (bootstrapped) to generate a distribution of equipment-level emission factors. Here, we will briefly explain the development of equipment level emission factor distributions.

The general stochastic failure equation is as follows. For a single piece of equipment:

$$EF = \sum_{k=1}^{n_c} \sum_{l=1}^{LQ_k} CF_k \tag{2}$$

Based upon the number of leaking components, LQ_k, a corresponding number of leaks of the correct component type are randomly drawn from the database of component level emission factors (with replacement). Here, a single draw is identified as CF_k. The equipment level emission factor is then calculated by summing across all components.

Recall that our dataset has been split at a threshold of 10,000 ppmv (describing leaks that were missed by optical gas imaging but caught with Method 21 below the threshold, and leaks that were caught with optical gas imaging above the threshold). Therefore, we can rewrite the previous equation as follows:

$$EF = \sum_{k=1}^{n_c} \left\{ \sum_{l=1}^{LQ_k'} (CF_k)_{lo} + \sum_{l=LQ_k'}^{LQ_k} (CF_k)_{hi} \right\} \tag{3}$$

We have defined this split to (to the best of our ability) describe two distinct and mutually exclusive parts of the leaker distribution. Here the subscript 'lo' refers to the portion of the dataset tagged at <10,000 ppmv, and the subscript 'hi' refers to the portion of the dataset that were tagged above that threshold.

Next, we must define LQ', which describes the number of leakers that are drawn in the 'lo' set versus the 'hi' set. First, we will define the fraction leaking value, FL, as follows:

$$FL = \frac{LQ}{CQ} \tag{4}$$

Where CQ is the total components on a piece of equipment.

Earlier, when we derived fraction leaking values for the 'lo' and 'hi' concentration ranges based upon our source studies, we did this by splitting up the datasets according to the Method 21 and optical gas imaging techniques (See **Supplementary Table 10**).

Therefore,

$$(FL_{total})_k = \frac{(LQ_{total})_k}{CQ_k} = \frac{(LQ_{lo})_k}{CQ_k} + \frac{(LQ_{hi})_k}{CQ_k} = FL_{lo} + FL_{hi} \tag{5}$$

So, by the same logic:

$$LQ_{lo} = CQ \times FL_{lo} \tag{6}$$

$$LQ_{hi} = CQ \times FL_{hi} \tag{7}$$

Resetting the indices in our primary equation:

$$EF = \sum_{k=1}^{n_c} \left\{ \sum_{l=1}^{CQ\times FL_{lo}} (CF_k)_{lo} + \sum_{l\prime=1}^{CQ\times FL_{hi}} (CF_k)_{hi} \right\} \tag{8}$$

Therefore, the complete distribution can be defined as the superposition of the two halves, defined by separate sets of measured leaks and separate fraction leaking values:

$$EF = \sum_{k=1}^{n_c} \sum_{l=1}^{CQ\times FL_{lo}} (CF_k)_{lo} + \sum_{k=1}^{n_c} \sum_{l\prime=1}^{CQ\times FL_{hi}} (CF_k)_{hi} \tag{9}$$

$$EF = EF_{lo} + EF_{hi} \tag{10}$$

Note that for this manuscript, this process is adjusted such that the number of components leaking per piece of equipment (LQ) is tallied according to a random draw. This process is repeated for n_{trials} = 10,000 to develop emission factor distributions. In iterative loops, the algorithm sweeps across all equipment (n_e), all components (n_c), and all components per equipment ($CQ_{i,j,k}$).

$$LQ_{i,j,k} = \sum_{l=1}^{CQ_{i,j,k}} X_{i,j,k,l} \quad \forall\, i \epsilon \{1, \ldots, n_{trials}\}, j \epsilon \{1, \ldots, n_e\}, k \epsilon \{1, \ldots, n_c\} \tag{11}$$

Here **LQ** is a 3-dimensional matrix, with dimensions 10,000 x n_e x n_c. During the iteration across each component class, $X_{i,j,k,l}$ is a random binary variable set equal to 1 if a uniformly distributed random number (p) on [0,1] is drawn that is greater than FL for that component type. Put differently, for each leak draw *l*, if the random number drawn is less than the probability of leakage then $LQ_{i,j,k}$ is incremented by 1.

$$X_{i,j,k,l} = \begin{cases} 1 & p \leq FL \\ 0 & p > FL \end{cases} \tag{12}$$

The resulting matrix element $LQ_{i,j,k}$ is the number of leaks for each component type *k* in each equipment type *j*, for each realization *i* (of which there are 10,000).

Next, the algorithm assigns leakage volumes to these leaks. Based upon the number of leaks, $LQ_{i,j,k}$, a corresponding number of leaks of the correct component type are randomly drawn from the database of component level emission factors (with replacement). This process is repeated 10,000 times producing a second 3-dimensional matrix, **EF**, with dimensions 10,000 x n_e x n_c. Each element is calculated as follows:

$$EF_{i,j,k} = \sum_{l=1}^{LQ_{i,j,k}} CF \quad \forall\, i \epsilon \{1, \ldots, n_{trials}\}, j \epsilon \{1, \ldots, n_e\}, k \epsilon \{1, \ldots, n_c\} \tag{13}$$

The matrix **EF** is then reduced to 2 dimensions by summing across components, or summing across index *k*. This results in a new matrix $\mathbf{EF}_{i,j}$ with values of emissions for 10,000 trials

(index i) and n_e equipment types (index j). The Matlab tool "prctile" is used to produce probability distributions of emissions per piece of equipment using this matrix.

After superposing the separate equipment-level distributions for < 10,000 ppmv measurements and ≥ 10,000 ppmv measurements, the resulting distributions are embedded in OPGEE's CH_4 emissions calculator as a 0 - 100 percentile table for each equipment type j in $1 \ldots n_e$. Because we have separate component counts for gas and oil systems, we also have separate equipment level emission factor distributions.

It should be noted that the emission factor distributions in **Supplementary Table 12** (*a priori* distributions) are different from the emission factor distributions presented earlier in **Supplementary Table 3** and **Supplementary Table 4** (*post-hoc* distributions). The distributions presented below are eventually applied to OPGEE's CH_4 emissions algorithm, where OPGEE iteratively assigns *a priori* emission factors. However, due to the conservation of mass checks, some emission factor draws are not assigned. *Post hoc* emission factors are calculated according to the actual emissions randomly assigned by OPGEE's iterative bootstrapping algorithm. Based on the OPGEE conservation of mass check, *post-hoc* emission factors are lower than the average *a priori* emission factors. This also results in marginal wells emission factors much lower than non-marginal wells emission factors.

Supplementary Table 12: Averages of a priori equipment level emissions factor distributions [kg day^{-1}]. Total emission factor distributions are the superposition of the 500-10,000 ppmv and >10,000 ppmv distributions.

	Natural gas systems			Petroleum systems		
	Total	500-10,000 ppmv	> 10,000 ppmv	Total	500-10,000 ppmv	> 10,000 ppmv
Well heads	4.6	1.4	3.3	3.3	1.1	2.2
Header	4.2	1.3	2.9	9.7	3.3	6.4
Heater	2.9	0.8	2.2	2.7	0.9	1.9
Separator	5.5	1.5	4.0	3.9	1.2	2.7
Meter	3.8	0.8	3.0	2.6	0.6	2.0
Tanks - Leaks	1.2	0.3	0.8	1.8	0.5	1.2
Tanks - Vents	12.1	1.2	10.9	12.3	1.2	11.2
Compressor - Recip	7.5	2.4	5.2	2.6	0.8	1.8
Dehydrator	5.0	1.3	3.7	2.2	0.6	1.5
Chemical Injection Pump	7.0	0.0	7.0	7.0	0.0	7.0
Pneumatic Controller	3.2	0.1	3.2	3.2	0.1	3.2

4.3.2. Modelling tank emissions

In our discussion of tank emissions we will differentiate between tank emissions sources and tank emissions mechanisms. Emissions sources from oil and condensate storage tanks are classified as follows and represented graphically in **Supplementary Fig. 8**. For useful pictures and descriptions of components, see also [28].

- *Open vents (e.g., gooseneck open vent, mushroom open vent)*
- *Thief hatch*: The thief hatch is a sort of access door which allows maintenance access to the tank for sampling and gauging. The thief hatch also works in tandem with pressure-relief valves to maintain safe pressures in the tank.
- *Pressure-relief valve*: Whereas an open vent remains in a fixed position, a pressure-relief valve remains closed unless the pressure or vacuum in the tank increases to a set level at which the pressure-relief opens.
- *Other miscellaneous components*: Like other equipment, storage tanks are an assemblage of smaller components. Leaks are tracked and quantified on tanks like any other piece of equipment.

Note that a similar classification scheme is applied in aggregating emissions data for our database. Also, we assume that each tank contains one thief hatch and one pressure-relief valve

which contribute to emissions at the tank (see **Supplementary Table 6** and **Supplementary Table 7,** emissions from the open vent are accounted for in the purposeful venting, or flash emissions category). There is a high level of uncertainty in this activity count assumption, given a lack of publicly available component counts (Zimmerle et al. [29] also assume 1 thief hatch and 1 pressure-relief valve per tank, although this is not based on direct counts).

Mechanisms are the processes by which these emissions occur:

- *Flashing emissions*: Emissions resulting from the release of entrained gas in depressurized hydrocarbons from separators.
- *Stuck dump valve resulting in anomalous emissions magnitude*: There are situations where free gas within separators may be released from tanks (see elaboration in the main text). This could be the result of stuck dump valves (due to erosion of seals or solids preventing the valve from closing) or free gas still entrained within the oil or condensate.

Emissions from storage tanks are treated differently depending on whether they are equipped with emissions controls (e.g., flares, combustors, or vapor recovery units). For tanks equipped with controls, flashing emissions are destroyed or captured. US EPA NSPS subpart OOOO requires new or modified tanks (as of April 2011) with the potential to emit greater than 6 tons per year of VOC to install control devices such as flares or vapor recovery units designed to reduce volatile organic compound emissions by at least 95% [2]. However, according to EPA GHGRP 2015 data [30] (and after extrapolating to the total tank population, **Supplementary Table 27**) only 49% of tanks were controlled.

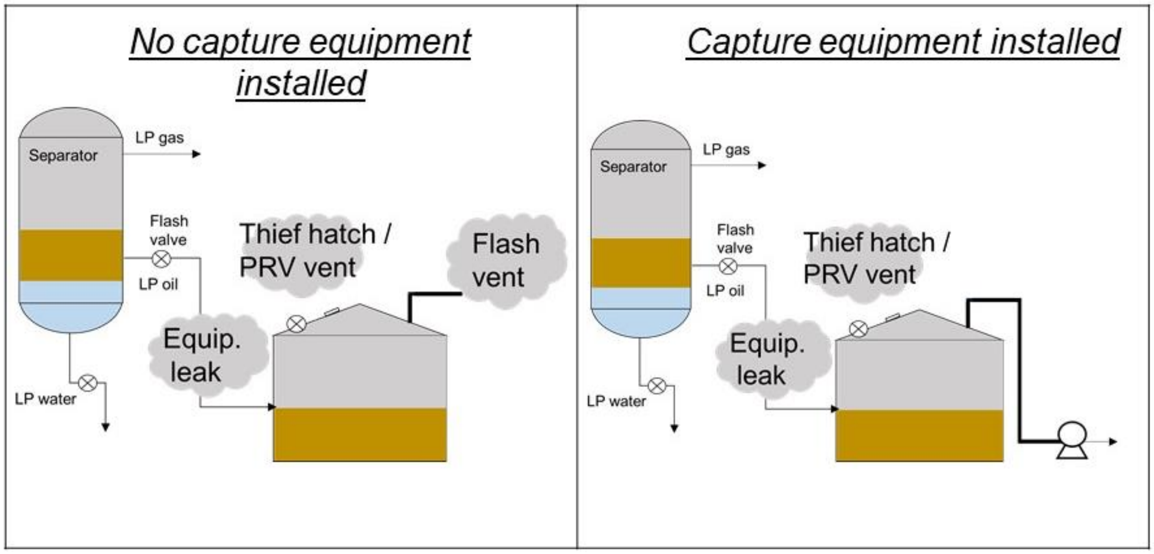

Supplementary Fig. 8: Illustrative comparison of tank emissions classes for tanks with and without control devices installed.

Methods for estimating the various emissions sources are outlined as follows:

Flash emissions: we follow generally the methods outlined by Zavala Araiza et al. [31]. Briefly, flash emissions are categorized as either continuous or intermittent based upon the maximum rate of dumping from separators (we use the estimate of 140 bbl day^{-1} from Zavala Araiza et al). Tanks receiving crude or condensate at a rate greater than the threshold are assumed to dump continuously, the rest are assumed to dump intermittently. For intermittent dumping, the fraction of tanks receiving dumped liquid at any given moment are assumed to be equal to the ratio of crude and condensate production and the 140 bbl day^{-1} threshold. Like Zavala Araiza et al. , the flash emission rate (FF, $kgCH_4$ bbl^{-1}) is based upon the HARC study [32], a direct measurement campaign of vent gas emissions from 33 tanks in the Barnett shale intended to reflect tank working, breathing, and flashing losses. The flash emission rate, FF, is converted to an emission factor ($kgCH_4$ $tank^{-1}day^{-1}$) using the following piecewise formula, depending on if the separator is dumping continuously or intermittently:

$$EF = \begin{cases} \begin{cases} FF \times {}^{140}/_{1.64} \times n_{wells}, & p < {}^{Q_{site}}/_{140} \\ 0, & p \geq {}^{Q_{site}}/_{140} \end{cases}, & Q_{site} < 140 \\ FF \times Q_{well} \times n_{wells}, & Q_{site} \geq 140 \end{cases} \quad (14)$$

On average, we assume, a site will contain one or more wells with a single separator. Therefore, in evaluating if a separator is dumping continuously or intermittently, we convert the well level throughput, Q_{well}, to a site level throughput, $Q_{site} = Q_{well} \times 1.64$. Here, 1.64 is the average number of wells per site according to Enverus data. p is a random number evaluated for every well.

- *Equipment leaks and vents due to equipment malfunction*: we assume equipment failures and leaks can occur at all tanks (both controlled and uncontrolled). Equipment leaks are calculated using the same "stochastic failure" approach as other equipment. For vents due to equipment malfunction, we also use the "stochastic failure" approach, but with the exception that we draw exclusively from ERG [12].

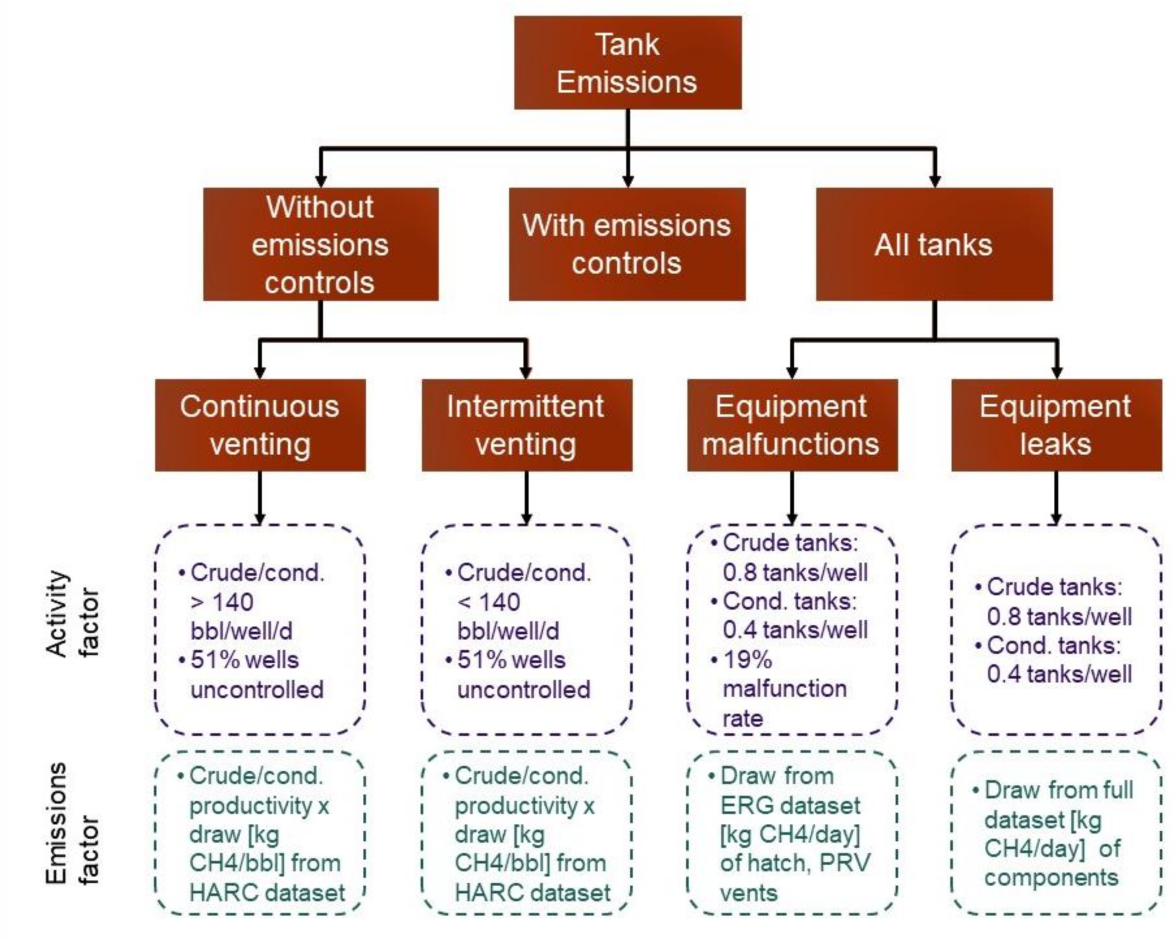

Supplementary Fig. 9: Emission mechanisms of crude and condensate storage tanks

Though we depict emissions mechanisms in **Supplementary Fig. 9** as if they are mutually exclusive, it must be acknowledged that there is likely overlap. In particular, the ERG dataset (source for equipment malfunction emissions, [12]) likely contains some emissions from flashing,

and the HARC study (source for flashing emissions, [32]) likely contains emissions from equipment malfunction. However, evidence from these studies suggests that the overlap could be minimal (see further description of these studies in Supplementary Methods 7).

- In the HARC study [32], tanks were inspected prior to vent measurements. Thief hatches were closed and any tanks with rusted holes were not measured.
- We removed three tank samples (17, 25, and 26) from the HARC dataset. Reanalysis by ERG (distinct from the previously mentioned Fort Worth campaign, [33]) suggested, based on what was measured versus the physically possible flash emissions, that these measurements were likely due to equipment malfunction. By removing these measurements, it is more likely that the HARC dataset only contains flash emissions, and not emissions due to equipment malfunction.
- Measurements by ERG [12] (used in our assessment of unintentional tank emissions) are from thief hatches, rusted holes, and PRVs, locations not sampled in the HARC study.

However, to provide a conservative estimate, we model uncertainty in double counting between the two tank emissions sources (See Supplementary Methods 5)

4.3.3. Liquids unloadings

In our model, methane emissions from liquids unloadings are characterized as follows:

$$E_{total} = E_{plunger} + E_{non-plunger} \tag{15}$$

Where *EF*' [scf event^{-1}] is the emission factor, or emissions per unloading event. *EF*' is "per event", and therefore needs to be corrected for F, the event frequency [events well^{-1}]. Total emissions are calculated by summing across all wells with unloading events.

Liquids unloading data are obtained from Allen et al. [34] (see description of study in Supplementary Methods 7). Direct measurements, obtained for wells with (both automatic and manual) and without plunger lifts, are used to define distributions of *EF*'. Survey data is used to define distributions of *F*, the event frequency.

4.3.4. Completions and workovers

Emission factors for completions and workovers are generated separately for categories of wells based on hydraulic fracturing, flaring, and reduced emission completions and workovers. For data we use reported Subpart W activity and emissions, downloaded from tables

“EF_W_COMP_WORKOVERS_FRAC” and “EF_W_COMP_WORKOVERS_NO_FRAC” on the “Envirofacts GHG Customized Search” website [35].

For each category, we sum reported CH_4 emissions and divide by the number of reported completion or workover events. Note that we use GHGRP reporting year 2016 data to develop differentiated oil and gas emission factors. 2016 is the first year oil wells were required to submit data on completions and workovers to the GHGRP [36]. Even though we use the same data source, our emission factors are still different from the GHGI. This is because the EPA develops combined completion and workover emission factors (in contrast to our approach, where the two source categories are separated, [37]). It is not clear from documentation why this approach was chosen.

Examining activity data in the 2020 GHGI, GHGRP subpart W counts are applied for some emission sources (e.g., natural gas system hydraulic fracturing completions) but not others (e.g., natural gas system non-hydraulic fracturing completions). This could be because subpart W reporting is still incomplete for certain categories (presumably, not all well completions are reported by operators). Therefore, for our estimation of total emissions we use emissions factors calculated using subpart W data and activity factors from GHGI (**Supplementary Table 13**).

Based on these calculations we find total completion and workover CH_4 emissions to be 46.9 Gg for the natural gas sector and 83.6 Gg for the petroleum sector.

Supplementary Table 13: 2015 emissions from completions and workovers. Emissions are based on emissions factors derived from GHGRP subpart W data and activity factors derived from the GHGI. Here, HF = hydraulic fracturing and REC = reduced emission completions.

	Emissions factor [tonne/event]	Activity factor [events/year]	Total [tonne/year]
	Natural gas systems		
HF Completions - Non-REC with Venting	8.103	105	850.8
HF Completions - Non-REC with Flaring	0.559	326	182.6
HF Completions - REC with Venting	6.404	3053	19550.6
HF Completions - REC with Flaring	5.500	1795	9871.7
Non-HF Completions - vented	22.656	602	13631.2
Non-HF Completions - flared	0.192	188	36.2
HF Workovers - Non-REC with Venting	4.339	199	864.6
HF Workovers - Non-REC with Flaring	1.266	66	84.1
HF Workovers - REC with Venting	0.516	1833	945.3
HF Workovers - REC with Flaring	0.812	339	275.0
Non-HF Workovers - vented	0.074	7303	537.4
Non-HF Workovers - flared	0.073	349	25.7
	Petroleum systems		
HF Completions: Non-REC with Venting	43.664	1494	65233.5
HF Completions: Non-REC with Flaring	1.266	1517	1920.8
HF Completions: REC with Venting	0.899	3630	3262.2
HF Completions: REC with Flaring	1.634	5494	8979.8
HF Workovers: Non-REC with Flaring	13.721	267	3663.6
HF Workovers: REC with Flaring	0.598	976	583.3

4.4. Equipment-level activity factors

In the GHGI, direct equipment counts are not available for every year. As an approximation, the GHGI uses "activity drivers" (AD) such as gas production, number of producing wells, or system throughput. AD are multiplied by a scaling factor (e.g., separators per well, af_k^*) derived from a subsample of the population. For each piece of equipment, we employ well counts as the activity driver, AD. Since the 2018 GHGI, the EPA has calculated activity factors for most equipment using scaling factors based on GHGRP data. Scaling factors based upon reporting year 2015

equipment counts are multiplied by year-specific wellhead counts to calculate year-specific equipment counts [38].

$$af_k = AD \cdot af_k^* \tag{16}$$

For this study, equipment-level activity factors are calculated using the same approach as the GHGI (documented in [30,38]). Therefore, any differences between activity factors of our study and the GHGI are due to relatively minor differences in well counts. This is probably because Enverus well counts (the source for both our study and the GHGI, see Supplementary Methods 5) are revised on a regular basis). Briefly, equipment count data from EPA's Envirofacts was used (reported by operators under Subpart W). Equipment count data for reporting year 2015 was downloaded from several tables: "EF_W_ATM_STG_TANKS_CALC1OR2", "EF_W_ATM_STG_TANKS_CALC3", "EF_W_NGPNEUMATIC_DEV_UNITS", and "EF_W_EQUIP_LEAKS_ONSHORE". Activity scaling factors were calculated by dividing reported equipment counts by reported wells, separately for natural gas systems and petroleum systems (**Supplementary Table 14**).

These scaling factors, af_k^*, are applied for each equipment *k*. Because our algorithm iterates across equipment count, the need for AD in the formula explicitly disappears. For each bin, *i*, emissions are calculated well-by-well. For a single well, *j*, equipment-level emissions are calculated by multiplying a randomly drawn emissions factor, $EF_{i,j,k}$ (kg equipment^{-1}day^{-1}), by its respective activity scaling factor, af_k^* (number of equipment per well). In the OPGEE CH_4 emissions algorithm, af_k^* is represented as follows.

$$Q_{population} = \sum_{i=1}^{n_{bins}} \left\{ \sum_{j=1}^{n_{wells,i}} \left[\sum_{k=1}^{n_{equip}} EF_{i,j,k} * X \right] \right\} \tag{17}$$

Where:

$$X = \begin{cases} 1 & p \leq af_k^* \\ 0 & p > af_k^* \end{cases} \tag{18}$$

Supplementary Table 14: Scaling factors applied in modelling equipment activity.

Equipment	Scaling factor (count per well)1
Natural gas system	
Heater	0.13
Separator	0.71
Dehydrator	0.03
Meters	0.84
Small Recip compressor	0.08
Chemical Injection Pumps	0.20
Pneumatic controllers	1.87
Tanks	0.41
Petroleum system	
Heater-treater	0.19
Separator	0.37
Header	0.22
Chemical Injection Pumps	0.09
Pneumatic controllers	1.11
Tanks	0.82

[1]Scaling factors are based on counts of equipment divided by counts of wellheads in several tables reported on the EPA Envirofacts website, reported under Subpart W for reporting year 2015. For tanks, data is from "EF_W_ATM_STG_TANKS_CALC1OR2" and "EF_W_ATM_STG_TANKS_CALC3". For pneumatic controllers, data is from "EF_W_NGPNEUMATIC_DEV_UNITS". Chemical injection pumps are reported an EPA memo [38]. All remaining scaling factors are based on equipment counts reported in "EF_W_EQUIP_LEAKS_ONSHORE".

5. Supplementary Methods 5: Producing a production-segment CH_4 estimate

This section describes how our CH_4 emissions tool, implemented in the Oil Production Greenhouse Gas Emissions Estimator (OPGEE), is used to generate an estimate of US oil and natural gas (O&NG) production segment CH_4 emissions. We begin by describing our well and O&NG production dataset, and how this dataset was translated to representative OPGEE fields for analysis. Next, we describe minor adjustments to how several emissions sources (methane slip, completions and workovers, and methane from flare stacks) were modelled and how additional analysis was performed to make our results comparable to site-level studies. We conclude with a summary of how uncertainty is treated in our model.

5.1. Development of representative "fields" for analysis

On the OPGEE Inputs sheet, out of the >50 possible inputs, only a small number are required for analysis of CH_4 emissions. These include O&NG production, well count, gas to oil ratio (GOR), CH_4 content, and a categorical liquids-unloading variable.

5.1.1. Gas composition

The CH_4 content reported in the EPA GHGI ([5], Table 3.6-3) is itself based upon the Gas Technology Institute Unconventional Natural Gas and Gas Composition Databases [39]. The 2015 average CH_4 content, weighted by regional production, reported in the GHGI is 82.2% (volume basis).

However, as GHGRP data is becoming more widely used for GHGI source categories, this reported gas composition gets applied indirectly (operators are required to report CH_4 content under Section 98.236(aa)(1)(ii)). The CH_4 mole fraction used in OPGEE is based on average mole fractions from reporting year 2015 Table "EF_W_FACILITY_OVERVIEW". Using the same approach as the EPA in partitioning facilities (summarized in Supplementary Methods 6) we compute average mole fractions for natural gas and petroleum systems, 83.2% and 68.3%, respectively. These values are applied in OPGEE.

5.1.2. Well count and O&NG production dataset

Well count, oil production, and GOR are sourced from Enverus [40]. While the home institution of authors JSR and ARB (Stanford University) has direct access to Enverus, for the purposes of comparison with the Alvarez et al. [4] study, we requested the Enverus (previously known as DrillingInfo) dataset used in that study to base our analysis on. These data were obtained as "Drillinginfo_US_2015_Production_Data_Wells2Sites.xlsx" from David Lyon on 19/8/14.

The data were then filtered. Federal offshore wells were removed by filtering column "STATE" for "FO GULF" and "FO PACIFIC" and deleting the associated rows. This corresponded to 5,201 wells, 1,350 sites, and ~1 Tcf/yr gas production. Inactive wells were also removed (1,202 wells, 0 Tcf/y gas production).

In this dataset wells had been previously clustered by David Lyon into well sites based upon proximity within a 50 m radius (see further description in the Alvarez et al. Supplementary information [4]). For the purposes of our analysis, well-site production data was de-clustered into well-level data. For each multi-well row in the array, new rows were created according to the number of wells in the site, and production was assigned according to the average well productivity at the site.

Comparing well counts from the Alvarez Enverus dataset, our total count for onshore wells is ~15,000 wells lower compared to the EPA GHGI total (year 2015 data, as reported in 2020 inventory [5]). However, the EPA also uses the Enverus dataset for well counts [41]. In **Supplementary Table 15**, we compare Enverus totals for onshore only with totals including offshore to see if this accounts for the difference, yet even including offshore wells there is still a ~10,000 difference between our totals and the EPA (note that this comparison is only for illustrative purposes, and offshore wells are not included in OPGEE modelling). Various filtering steps in Enverus could account for the differences. Also, Enverus is continually updating its database, and totals may differ depending on when data was downloaded. A difference of 15,000 is only 1.5% of total wells and will therefore not significantly affect emission results.

Supplementary Table 15: Well counts for natural gas and petroleum systems in our study compared with totals from the Greenhouse Gas Inventory. Our study (based on data from Enverus) only models onshore wells, but we also present "onshore + offshore" totals for comparative purposes.

	Wells	Total prod. (MMbbls year^{-1})	Total prod. (Bscfs year^{-1})
	Enverus database - Onshore only		
Gas	433,430	22	18,935
Oil	571,761	2,763	12,794
Total	1,005,191	2,786	31,729
	Enverus database - Onshore + offshore		
Gas	433,881	24	19,334
Oil	576,511	3,367	13,851
Total	1,010,392	3,391	33,184
	EPA GHGI		
Gas[1]	419,692		
Oil[2]	600,519		
Total[3,4]	1,020,211	2,878	31,807

[1]As reported in the row "Total Active Gas Wells" of Table 3.6-7
[2]As reported in the row "Total Oil Wells" of Table 3.5-5
[3]Total oil production reported as the activity factor for "Miscellaneous Production Flaring" on Table 3.5-5
[4]Total gas production reported as the activity factor for "Misc. Onshore Production Flaring" on Table 3.6-7

US wells were stratified into smaller representative subsamples, or fields on the basis of GOR and well-gas productivity. First, the dataset was split into four groups applying the same GOR cutoff as the EPA [41]: (i) Gas only wells, (ii) gas wells with oil production (GOR > 100 Mscf bbl^{-1}), (iii) oil wells with associated gas (GOR < 100 Mscf bbl^{-1}), (iv) and lastly oil only wells (no gas production reported). Note that the EPA does not distinguish oil wells and gas wells from associated wells (i.e., oil wells with gas production and gas wells with oil production), but this separation was conducted for our analysis. Dry gas wells are analyzed in a separate category from gas wells with oil production because unique treatment is required in OPGEE (OPGEE requires a nominal amount of liquids production to simulate dry gas wells). Oil wells are analyzed separately from oil wells with associated gas for the purposes of an additional calculation step (we assume a small amount of associated gas is produced, even if no marketed gas is reported).

From these datasets, we then split into ten productivity tranches for gas only, gas with oil, and oil with gas datasets, and four productivity tranches for the oil-only dataset (See **Supplementary Table**

16-19). The productivity tranches are defined using lower and upper cutoffs in mean daily gas production rate measured in Mscf day^{-1}.

Supplementary Table 16: Gas wells (gas only) productivity tranches.

Bins (Mscf well^{-1}day^{-1})	Count	Mean gas rate (Mscf well^{-1}day^{-1})	Total gas (Mscf day^{-1})	Total oil production (bbl day^{-1})
0.00-1.00'	38,225	0.4	15,065	0
1.00-5.00'	65,138	2.8	180,330	0
5.00-10.00'	43,154	7.3	313,240	0
10.00-20.00'	44,523	14.4	642,190	0
20.00-50.00'	57,148	32.4	1,851,000	0
50.00-100.00'	27,868	69.5	1,938,200	0
100.00-500.00'	31,429	228.9	7,193,200	0
500.00-1000.00'	6,727	690.3	4,643,900	0
1000.00-10000.00'	5,875	2,571.8	15,109,000	0
10000.00-inf	87	20,103.0	1,748,900	0

Supplementary Table 17: Gas wells (with oil production) productivity tranches.

Bins (Mscf well^{-1}day^{-1})	Count	Mean gas rate (Mscf well^{-1}day^{-1})	Total gas (Mscf day^{-1})	Total oil production (bbl day^{-1})
0.00-1.00'	400	0.6	246	1
1.00-5.00'	2,880	3.0	8,648	39
5.00-10.00'	3,907	7.5	29,482	111
10.00-20.00'	9,384	15.2	142,900	496
20.00-50.00'	30,938	34.5	1,068,800	3,673
50.00-100.00'	29,916	71.1	2,126,500	6,929
100.00-500.00'	30,031	197.9	5,943,900	20,835
500.00-1000.00'	3,094	696.6	2,155,300	8,337
1000.00-10000.00'	2,684	2,376.7	6,379,100	19,625
10000.00-inf	22	15,955.0	351,000	1,133

Supplementary Table 18: Oil wells (with gas production) productivity tranches.

Bins (Mscf well^{-1}day^{-1})	Count	Mean gas rate (Mscf well^{-1}day^{-1})	Total gas (Mscf day^{-1})	Total oil production (bbl day^{-1})
0.00-1.00'	65,606	0.4	23,107	192,790
1.00-5.00'	89,163	2.6	231,830	425,170
5.00-10.00'	38,948	7.2	280,540	208,860
10.00-20.00'	44,410	14.4	638,950	294,520
20.00-50.00'	54,343	31.9	1,735,300	581,920
50.00-100.00'	32,775	71.1	2,331,500	856,460
100.00-500.00'	42,848	216.8	9,290,800	2,722,700
500.00-1000.00'	7,280	687.2	5,003,100	953,610
1000.00-10000.00'	4,464	2,213.1	9,879,200	676,700
10000.00-inf	348	15,331.0	5,335,200	99,644

Supplementary Table 19: Oil wells (oil only) productivity tranches.

Bins (Mscf well^{-1}day^{-1})	Count	Mean gas rate (Mscf well^{-1}day^{-1})	Total gas (Mscf day^{-1})	Total oil production (bbl day^{-1})
0.00-0.50'	114,460	0.2	18,147	38,110
0.50-1.00'	27,149	0.7	19,472	40,256
1.00-10.00'	45,205	3.0	134,040	270,320
10.00-inf	4,759	22.2	105,840	204,490

5.1.3. Liquids unloading

These datasets are split one final time into OPGEE columns based on liquids unloading. For gas wells, 10.4% have liquids unloading events with plunger lift and 7.1% without plunger lift [5]. Oil wells (GOR <100 Mscf bbl^{-1}) do not require liquids unloading events.

5.1.4. Treatment of "oil only" wells

For wells reporting zero gas production to Enverus (henceforth referred to as oil-only wells), we assume some amount of unreported gas is produced and could be lost as venting or leakage emissions before use. A similar analysis is conducted by Alvarez et al. [4]. This is because even

very heavy oil contains some dissolved hydrocarbon gases. To approximate gas co-produced from oil wells, we need to calculate the gas-oil ratio. The solution gas-oil ratio, R_s, is a function of the oil and gas composition, pressure, and temperature. R_s reaches a maximum at pressures beyond the bubblepoint pressure, P_b. As the pressure drops below P_b, R_s decreases as gas comes out of solution. We calculate R_s at P_b, and assume that when the crude oil is exposed to atmospheric pressure at the surface in atmospheric storage tanks at ambient temperatures, all gas evolves.

We use the following empirical relationship from Al Shammasi [42], as reported in Fanchi Volume 1 [43]:

$$R_s = P_b^{a_2^{-1}} e^{\frac{a_3}{a_2}\gamma_o\gamma_g} \gamma_o^{-\frac{a_1}{a_2}} \gamma_g^{-1} (T_r + 459.67)^{-1} \tag{19}$$

The following assumptions are made based on the average conditions from the reservoir sample in Fanchi (pg 265): T_r = 185ºF, P_b = 2041 psia, γ_g = 0.774 (see Fanchi for coefficients a_1, a_2, a_3). Holding these parameters fixed, we now have a relationship for solution gas-oil ratio (R_s) variation with crude API gravity (γ_o).

Although there are columns for API gravity in the Enverus dataset, these columns are relatively sparse (and for specific basins, there is no data at all). Therefore, for data on API gravity we use the Wood MacKenzie (WM) dataset. Rather than at a well-level, API gravity exists in the WM dataset at the field-level. This dataset was processed to produce a field-level API gravity dataset of 2130 entries. The data set is further filtered for rows classified as oil fields, with non-zero oil production and API gravity > 0 (382 rows remain).

Next, the Fanchi equation is used to calculate R_{so} from API gravity. We find an average API gravity of 34.8 API and an average R_{so} of 503 scf bbl^{-1}. This is larger, but a similar order of magnitude, compared to a similar analysis performed by Alvarez et al [4].

R_{so} values are randomly assigned to oil-only rows in the Enverus dataset as we do not have API values in most cases.

5.2. Deviations from standard OPGEE functionality in this paper

Due to the specific application of this study, some modifications were made to the standard OPGEE functionality. These modifications are described in this section.

5.2.1. Autorun macro

Although OPGEE 3.0 contains built in Monte Carlo uncertainty functionality, the application of this paper required a modified approach. The existing OPGEE 3.0 Monte Carlo functionality is designed to test uncertainty in missing data and the use of smart default parameters, but it is not designed to robustly test the full uncertainty in the CH_4 emissions tools. For this study, we develop a Excel macro titled Autorun, described in this section. Although it is not currently, it is possible that Autorun could be formally incorporated into future OPGEE 3.0 versions.

Due to the randomized nature of the component-level bootstrapping algorithm, equipment-level emission factor distributions passed to OPGEE are variable. To explore how this uncertainty propagates to our final CH_4 emissions estimates, we generate 100 unique equipment-level emission factor distributions and export these distributions to .CSV files. In OPGEE, new equipment-level emission factor files are imported after each Monte Carlo iteration.

The OPGEE 3.0 model applied in this analysis was adjusted such that with an Inputs sheet setting “Monte Carlo realizations input = 1”, OPGEE still runs in random mode. Although we still want OPGEE to make random draws, we only want one internal OPGEE realization (our 100 Monte Carlo realizations are run outside of OPGEE in the Autorun wrapper).

5.2.2. Methane slip

Compressor fuel use and emissions are calculated in OPGEE as a function of gas throughput and the required compression ratio between wellhead pressure and separator working pressure. Because (i) this approach is not consistent with the inventory approach of this paper (e.g., calculation of per well emissions) and (ii) we are concerned that the current approach in OPGEE underestimates emissions from methane slip, for this paper we run a separate calculation external to OPGEE.

Emissions factors for methane slip from natural gas reciprocating engines are calculated according to an adjusted emissions factor (EF', kg hp^{-1}hr^{-1}) and the power rating of the engine (P , hp).

$$EF = EF' \times P \times 24 \quad (20)$$

Following Zavala Araiza et al. [31], EF' is assigned a normal distribution with a mean of 0.00125 kg hp^{-1}hr^{-1}. This number is based on NSPS specifications, with a 25% adjustment upwards to account for real-world performance [31,44]. The standard deviation of the normal distribution (σ = 0.000319 kg hp^{-1}hr^{-1}) reflects 95% CI of +/- 50% around the mean. Power ratings of on-site reciprocating compressors are drawn randomly from the ERG data set [12], which includes a record of all compressors found in their Fort Worth campaign. It is not clear how representative the wellpad compressor power distribution from the ERG study is of the national distribution.

5.2.3. Completions and workovers

OPGEE 3.0's primary functionality is to calculate a carbon intensity (mass of carbon dioxide equivalent per megajoule of primary energy produced). In calculating carbon intensity, completion and workover emissions are amortized over the lifetime energetic production of the well. However, for this calculation we are interested in a gross, non-amortized emission estimate for 2015. Rather than work around these calculations in OPGEE, we calculate non-amortized completion and workover emissions outside of OPGEE.

5.2.4. Methane from flare stacks

In the EPA's GHGRP, uncombusted CH_4 from flares is calculated as follows:

$$\text{Flare CH}_4 = V_{gas,flare} \times X_{CH_4} \times [(1-\eta) \times Z_{lit} + Z_{unlit}] \quad (21)$$

Here Flare CH_4 (scf) is calculated based upon the volume of gas sent to the flare ($V_{gas,flare}$, scf), the mole fraction of CH_4 (X_{CH_4}), flare combustion efficiency (η, fraction gas combusted), fraction of feed gas sent to a burning flare (Z_{lit}), and fraction of feed gas sent to an un-lit flare (Z_{unlit}).

Calculation of the GHGI emissions estimates based on GHGRP data is described in Supplementary Methods 6. Briefly, the operators report flaring activity and emissions to the GHGRP under the categories of well completions and workovers, routine venting of flash gas from storage tanks, flaring of associated gas, and other miscellaneous activities not covered under other categories. Although emissions are spread across various reporting tables, activity data is contained within the single table “EF_W_FLARE_STACKS_UNITS”. This table is useful for parameterizing activity data for our modified approach.

As reported in **Supplementary Table 39**, many operators apply the default flare combustion efficiency (η) of 98% and rarely report gas sent to unlit flares. We implement a similar approach in OPGEE, but with modifications to estimated flare combustion efficiency and fraction of gas sent to unlit flares. First, we calculate an equipment-based activity factor for number of flare stacks per well using a well-based extrapolation procedure as described in Supplementary Methods 6, with an adjustment for number of flare stacks per well:

$$n_{stacks,pop} = \left(\frac{n_{wells,pop}}{n_{wells,subW}}\right) \times n_{stacks,subW} \quad (22)$$

Here, we use the same approach as the EPA to partition Subpart W data into natural gas and petroleum system categories (see Supplementary Methods 6, based on reported formation type by facility).

Supplementary Table 20: The total count of flare stacks in the United States oil and natural gas production segment.

	Natural gas systems	Petroleum systems
Total wells	433,430	571,761
Reported flaring wells[2]	98,117	149,884
Reported wells[1]	290,710	214,993
Flaring wells per reported wells	0.3	0.7
Reported flare stacks[3]	9,313	13,012
Reported flaring wells	98,117	149,884
Flare stacks per reported flaring wells	0.1	0.1
Total flare stacks	13,885	34,605

[1]The number of wells reporting to "EF_W_FACILITY_OVERVIEW" is different from the number of wellheads reported to "EF_W_EQUIP_LEAKS_ONSHORE"
[2]Calculated by matching wells reporting to "EF_W_FACILITY_OVERVIEW" with facilities reporting to "EF_W_FLARE_STACKS_UNITS"
[3]Counted by summing unique flare stacks reported to "EF_W_FACILITY_OVERVIEW"

To gain a better understanding of how the EPA flaring data set compares with other studies, we will use the parameter "GAS_SENT_TO_FLARE" as a basis for comparison. Filtering for onshore petroleum and natural gas production, the sum of "GAS_SENT_TO_FLARE" is 86.1 Bscf year^{-1}. This is the total flared gas reported to the GHGRP. If we use a well-based extrapolation (see definition of method in Supplementary Methods 6), then we multiply this total by approximately 2 (505,703 wells reported to 1,005,191 total wells) to estimate a population total of 172.1 Bscf/year. This is very close to the EIA estimate, but smaller than the VIIRS estimate for the production segment (**Supplementary Table 21**).

Supplementary Table 21: Comparison of different estimates for total quantity of gas flared. Estimates included are by by EPA Subpart W reporting [35], NOAA VIIRS [45] and EIA [46]

	Subpart W gas flared [Bscf/year]	Extrapolation factor	Total gas flared [Bscf/year]
EPA (Subpart W extrapolated)	86.1	2.0	172.1
NOAA VIIRS			245.6
EIA			170.9

In incorporating flare stacks into OPGEE, we assume high throughput flares are located at high productivity wells and low throughput flares are located at low productivity wells. Although it is very possible that many smaller wells might route to a single flare, this assumption allows us to utilize our existing productivity tranches to simulate variability in the volume of gas sent to flares. Like we describe in Supplementary Methods 6, the GHGRP Table "EF_W_FLARE_STACKS_UNITS" only reports emissions for miscellaneous flaring activities, but it contains all activity data for all flaring activities (e.g., miscellaneous flares, flaring of associated gas, flaring from crude and condensate storage tanks). Activity data is reported for all parameters in the flaring equation on a flare basis (i.e., each row corresponds to one flare stack). Therefore, to account for the distribution of gas volumes sent to flares we take the sample of $V_{gas,flare}$ from "EF_W_FLARE_STACKS_UNITS" and randomly draw 48,490 gas volumes. Note that these gas volumes are corrected for the default CH_4 composition in OPGEE (68.3% for oil sites and 83.2% for gas sites).

$$\left(V_{gas}\right)_{corrected} = \left(V_{gas}\right)_{subpartW} \times \frac{\left(X_{CH_4}\right)_{subpartW}}{\left(X_{CH_4}\right)_{OPGEE}} \tag{23}$$

These flare stack volumes are then binned into the same tranches used for well productivity volumes (**Supplementary Table 16-19**) to determine the fraction of wells flaring for each tranche. This parameter is treated like an equipment-level activity factor. For our two sets of natural gas and petroleum system tranches (**Supplementary Table 16-17** for natural gas systems and **Supplementary Table 18-19** for petroleum systems), flare stacks are assigned proportionally to well count.

Supplementary Table 22: Counts of flare stacks per well for oil wells with associated gas. This table corresponds to productivity tranches in **Supplementary Table 18**.

Bins (Mscf well^{-1}day^{-1})	Counts of wells	Count of flare stacks	Flare stacks per well
0-1	65606	5406	0.082
1-5	89163	5360	0.060
5-10	38948	2341	0.060
10-20	44410	2891	0.065
20-50	54343	2199	0.040
50-100	32775	1025	0.031
100-500	42848	616	0.014
500-1,000	7280	47	0.006
1,000-10,000	4464	24	0.005
10,000-inf	348	0	0.000

Supplementary Table 23: Counts of flare stacks per well for oil wells with no associated gas. This table corresponds to productivity tranches in **Supplementary Table 19**.

Bins (Mscf well^{-1}day^{-1})	Counts of wells	Count of flare stacks	Flare stacks per well
0-0.5	114,460	9432	0.082
0.5-1	27,149	2237	0.082
1-10	45,205	2717	0.060
10-inf	4,759	310	0.065

Supplementary Table 24: Counts of flare stacks per well for dry natural gas wells. This table corresponds to productivity tranches in **Supplementary Table 16**.

Bins (Mscf well^{-1}day^{-1})	Count	Counts of flare stacks	Flare stacks per well
0-1	38225	8326	0.218
1-5	65138	2975	0.046
5-10	43154	1426	0.033
10-20	44523	386	0.009
20-50	57148	154	0.003
50-100	27868	30	0.001
100-500	31429	20	0.001
500-1,000	6727	3	0.000
1,000-10,000	5875	1	0.000
10,000-inf	87	0	0.000

Supplementary Table 25: Counts of flare stacks per well for natural gas wells with associated oil production. This table corresponds to productivity tranches in **Supplementary Table 17**.

Bins (Mscf $well^{-1}day^{-1}$)	Count	Counts of flare stacks	Flare stacks per well
0-1	400	87	0.218
1-5	2880	132	0.046
5-10	3907	129	0.033
10-20	9384	81	0.009
20-50	30938	83	0.003
50-100	29916	32	0.001
100-500	30031	19	0.001
500-1,000	3094	1	0.000
1,000-10,000	2684	0	0.000
10,000-inf	22	0	0.000

However, it is worth mentioning that due to some discrepancies in the GHGRP data, our method of utilizing activity data in "EF_W_FLARE_STACKS_UNITS" might present a lower bound. According to 98.236(n) all flare stacks must be reported. A possible source of confusion in how activity data is reported versus emission data could mean, however, that this is not the case. According to 98.233(n)(9) flare stack emissions in "EF_W_FLARE_STACKS_UNITS" "must be corrected for flare emissions calculated and reported under other paragraphs of this section to avoid double counting". Therefore, activity data in "EF_W_FLARE_STACKS_UNITS" should be comprehensive, but emissions data will not account for data already reported in completions and workovers, associated gas, and storage tank tables.

If we were to apply the flaring equation to activity data, then recalculated emissions should ideally match the sum of flaring emissions across all categories. Sorting for "onshore petroleum and natural gas production", we recalculated emissions in "EF_W_FLARE_STACKS_UNITS" using the flaring equation and calculated a total of 38 kt CH_4. This is lower than the sum of reported emissions across associated gas flaring, storage tanks, and reported miscellaneous emissions (11 + 44 + 6 = 61 kt CH_4). Clearly, some activity data is not being properly reported.

Supplementary Table 26: Comparison of flaring emissions reported to Subpart W.

Reported	Table	CH_4 emissions (kt)
Flare stacks (miscellaneous only)	EF_W_FLARE_STACKS_UNITS	11
Associated gas	EF_W_ASSOCIATED_NG_UNITS	44
Tanks	RY2015_ATM_STG_TANKS_CALC1OR2, RY2015_ATM_STG_TANKS_CALC3	6
Calculated emissions using Equation 98.233(n)		
Flare stacks	EF_W_FLARE_STACKS_UNITS	38

Emission factors are implemented in OPGEE as follows. For every well, flare emissions are calculated according to the draw of a uniformly distributed random number (p) on [0,1]. If the random number is drawn less than the probability of an unlit flare (f_{unlit}), then the emission factor is equal to the CH_4 produced at that well (Q_{well}) (as we described earlier, we allocate flare stacks assuming that flares are routed from single wells only). If the random number is greater than the probability of an unlit flare, then the emission factor is equal to the CH_4 produced at the well multiplied by the fraction of CH_4 uncombusted ($1 - \eta$).

$$EF = \begin{cases} Q_{well}, & p < f_{unlit} \\ Q_{well} \times (1 - \eta), & p > f_{unlit} \end{cases} \tag{24}$$

Instead of applying the EPA default combustion efficiency of 98%, we assume the distribution by Gvakharia et al. [47], based upon aerial surveys in the Bakken region.

For fraction of wells sending gas to unlit flares, we base our estimate on recent reporting for the EDF PermianMAP campaign [48]. Over four helicopter surveys, EDF observed a range of 4%-6% flares unlit. Conservatively assuming that these ranges might not be representative of other oil producing regions, we apply a range 3%-7% in OPGEE.

5.3. Grouping per-well model outputs into production sites for a consistent comparison with facility-level studies

The facility-level measurements synthesized by Omara et al. [20] and Alvarez et al. [4] aggregate, as the name implies, emissions from all equipment located at a production-site (e.g., well-pad, see definitions in Supplementary Methods 3). The nature of facility-level studies is to remotely measure all emissions from equipment that comprise a single production site, which includes one

or multiple wells, pumps, separation equipment, tanks, and potentially other processing equipment. In contrast, OPGEE produces emissions outputs for each type of equipment on a per well basis. To compare emissions results from Omara and Alvarez with our study, we needed to cluster our per-well results into facility-level groupings.

First, it should be noted that, although the Enverus dataset describes which wells are clustered into single-well and multi-well facilities, because wells were binned into specific columns for processing in OPGEE this detail was lost. To cluster our OPGEE results post-hoc, the following approach was taken:

1. For clustering, we use the well-count column from the same Enverus dataset described in the beginning of this Supplementary Methods section.
2. Both OPGEE output data and the Enverus dataset are binned on the basis of product stream – gas only, gas with associated oil, oil wells with associated gas – with the exception that oil only wells are removed (as they were in Omara et al. [20]). The Enverus dataset is further binned into the same productivity tranches as our OPGEE output results.
3. We iterate across rows (well, production, emissions combinations) in the OPGEE outputs and randomly assign a bin-specific well-count. Because OPGEE emissions outputs and the Enverus well count data have been binned in the same manner, any variation in wells per site across product class and productivity tranche is captured in our model.
4. According to the drawn well count, that number of per-well emissions are grouped into a site-level emissions value.

5.4. Uncertainty analysis

This study applies the Monte Carlo method to estimate uncertainty. Input parameters – component-level emission factors, component counts, and fraction of components emitting – are assigned distributions, and the range of uncertainty in these distributions is propagated through the model. Therefore, the full range of uncertainty is captured to the extent that these distributions encompass the full set of possible values.

A single OPGEE simulation will produce an estimate of total US CH_4, but it will not output a distribution. For a distribution, OPGEE must be run multiple times. We use the Autorun Excel macro, described in this Supplementary Methods section, to produce a distribution of 100 Monte Carlo results.

In this section we review treatment of uncertainty in the equipment-level emission factors, treatment of uncertainty in tank emissions, and conclude with a brief comment on the limitations of our uncertainty assessment.

5.4.1. Uncertainty in equipment-level emission factors

The uncertainty in component-level emission factor distributions is captured in the resampling algorithm discussed in Supplementary Methods 4. However, equipment-level emission factors are also a function of component counts and fraction of components emitting. Since a single equipment-level emission factor distribution is a function of constant component counts and fraction of components emitting values, a full assessment of uncertainty requires calculating multiple equipment-level emission factor distributions. Therefore, a different set of equipment-level emission factor distributions is used for each of our 100 Monte Carlo simulations. Distributions for component counts and fraction of components emitting are approximated as uniform distributions between the maximum and minimum values found in our surveyed studies (see **Supplementary Table 6** and **Supplementary Table 7** for component counts and **Supplementary Table 11** for fraction of components emitting). Unfortunately, our sparse dataset does not allow us to determine a likely distribution shape for these parameters so a uniform distribution is assumed for simplicity.

5.4.2. Uncertainty in tank emissions

In Supplementary Methods 4, we described how we treat tank flash emissions and abnormal venting emissions separately (based on the ERG and HARC sources [12,32]) but acknowledge that there is likely overlap between the studies drawn upon. To provide a conservative estimate of tank emissions, we model uncertainty in double counting between the two emissions sources (**Supplementary Fig. 8**). We denote the probability of an emissions event from HARC $P(A)$, and the probability of an emissions event from ERG $P(B)$, with the probability of intersection $P(A \cap B)$.

What we are interested in is the probability of an ERG emitting event, given an uncertain amount of overlap with HARC. We denote ERG emissions not overlapping with HARC as B'. In other words, what fraction of emissions events in the ERG dataset are emissions events due to equipment malfunction, not already represented in the HARC dataset? This can be denoted as:

$$P(\mathrm{B}') = P(\mathrm{B}) - P(\mathrm{A} \cap \mathrm{B}) \quad (25)$$

But how do we describe $P(A \cap B)$? We can conservatively assume that $A < B$ (or that an emissions event due to regular functioning is more likely than an emissions event due to irregular functioning. Therefore, the size of $A \cap B$ could be as small as zero (A and B are mutually exclusive) and as large as $P(B)$ ($P(B)$ is contained within $P(A)$).

To capture this range of possibilities in our modelling we adjust our fraction leaking values for tank venting from pressure relief valves. The lower bound is set at 0%, given the possibility of complete overlap between $P(A)$ and $P(B)$ (**Table S10**). The upper bound is that derived from ERG directly, which accounts for the possibility of null overlap between $P(A)$ and $P(B)$.

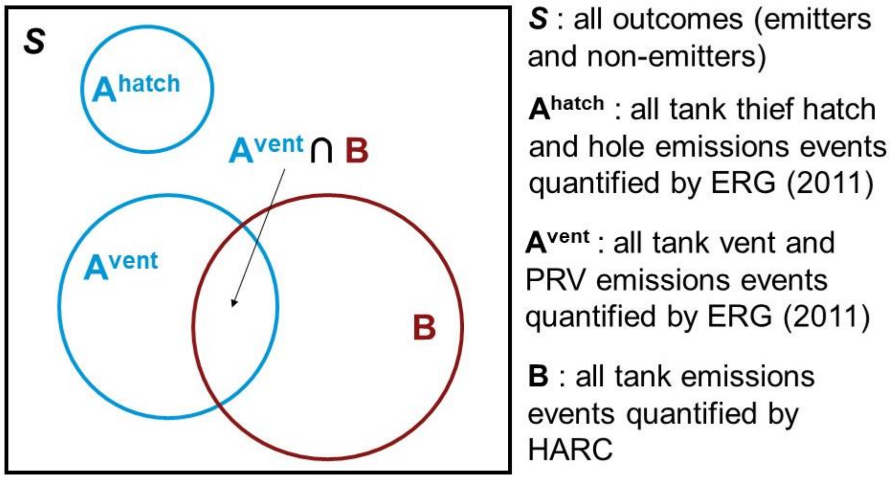

Supplementary Fig. 10: Venn diagram illustrating approach to uncertainty quantification for storage tank emissions. Our approach to uncertainty quantification specifically addresses possible overlap between the ERG [12] and HARC [32] tank emissions measurements.

5.4.3. Limits to uncertainty assessment of this study

Given the data limitations in component-level assessments, however, our distributions cannot capture the full range of uncertainty in component-level input parameters. Our dataset does not encompass all operators in the US, who may differ in leakage management. It’s also possible that our dataset doesn’t represent all possible configurations of equipment (e.g., component counts). Finally, we assume equipment-level activity factors (number of equipment per well) identical to the GHGI (which are themselves based on GHGRP data) although equipment counts will be

highly variable from site to site. This represents a significant source of uncertainty which is not captured by our model. Thus, it is reasonable to assume that the uncertainty ranges presented here are a lower bound on the possible ranges.

5.4.4. Monte Carlo uncertainty results

This manuscript is part of a larger project of improving CH_4 modelling in the Oil Production and Greenhouse Gas Emissions Estimator (OPGEE). OPGEE is a Microsoft Excel-based model which has slow run times. Because running a single instance of all ~1 million wells takes multiple hours, 100 bootstrapping iterations was the maximum allowable time for this manuscript.

Supplementary Table 27: Description of computer running OPGEE uncertainty realizations.

Operating System	MS Windows 10 Education
Excel version no.	MS Office Professional Plus 2016
Total Runs	100

In **Figure 1** and **Figure 2 (**main text**)**, we calculate error empirically (95% confidence interval based on the 2.5 and 97.5 percentile values extracted from the empirical distributions). Here, we will use a different approach to illustrate model convergence with increasing Monte Carlo realizations. For this calculation, we estimate a 90% confidence interval (CI) using the formula:

$$\bar{\mu} \mp 1.645 \frac{\bar{\sigma}}{\sqrt{n}} \tag{26}$$

Where $\bar{\mu}$ and $\bar{\sigma}$ are the sample mean and standard deviation, which we expect will converge towards the expected values of μ and σ, at a rate of $1/\sqrt{n}$, with sufficient model realizations *n*. This formula assumes that the distribution of error converges towards a normal distribution.

After 100 Monte Carlo realizations, total CH_4 emissions results converge on an error of approximately 6% (with 90% probability, see **Supplementary Fig. 11**).

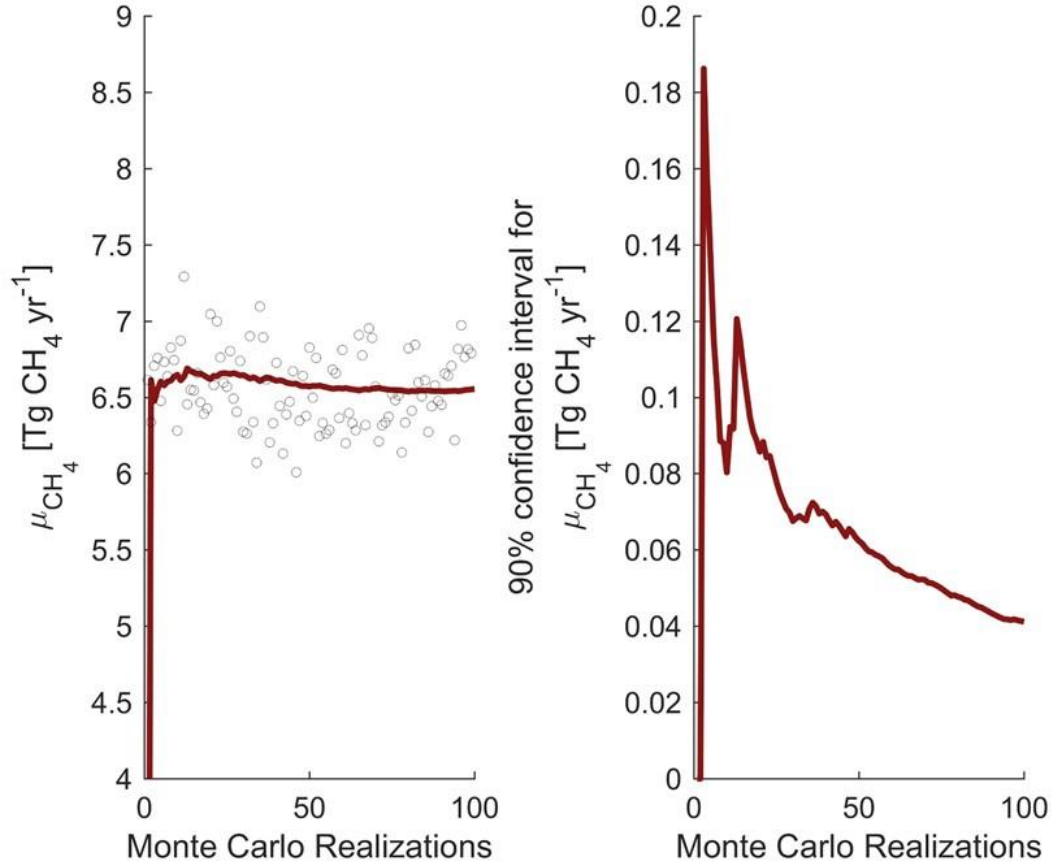

Supplementary Fig. 11: Monte Carlo uncertainty assessment of total CH_4 emissions. This figure illustrates evolution of (a) sample mean ($\bar{\mu}$) and (b) 90% confidence interval for $\bar{\mu}$ versus number of Monte Carlo realizations.

5.5. Further discussion of data and model representativeness

Dozens of distinct oil and natural gas (O&NG) producing basins are scattered across the US. Each of these regions will have different geological and operations related characteristics affecting CH_4 emissions. Some of these characteristics are addressed by our model, and some are not, suggesting the need for further research. In this section, we will begin by discussing the aspects that are captured by our model. We will conclude by addressing possible bias in what is not captured by our model.

The relationship between site-level productivity (Mscf $site^{-1}day^{-1}$) and production normalized CH_4 emissions has been discussed extensively in Omara et al. [6]. According to Omara, top-down

studies have demonstrated a wide variability in production normalized CH_4 emissions across basins, and “these trends are caused by differences in the distribution of both the number of sites and their natural gas production characteristics”. Basins such as Appalachia, Greater Green River, and Arkoma produce most of the gas from highly productive sites (>70% of gas from sites > 1,000 Mscf $site^{-1}day^{-1}$). Based on Omara’s model, which is calibrated to downwind, site-level surveys (see Supplementary Methods 2), these sites also demonstrate low production normalized CH_4 emissions (<1.5%). It is the opposite case for low productivity basins such as San Juan and San Joaquin (10% and 7% of gas, respectively, from sites > 1, 000 Mscf $site^{-1}day^{-1}$) which demonstrate high production normalized CH_4 emissions (>4%). In **Supplementary Fig. 12**, we demonstrate how our model reproduces the same relationship as Omara between site-level productivity and production normalized CH_4 emissions.

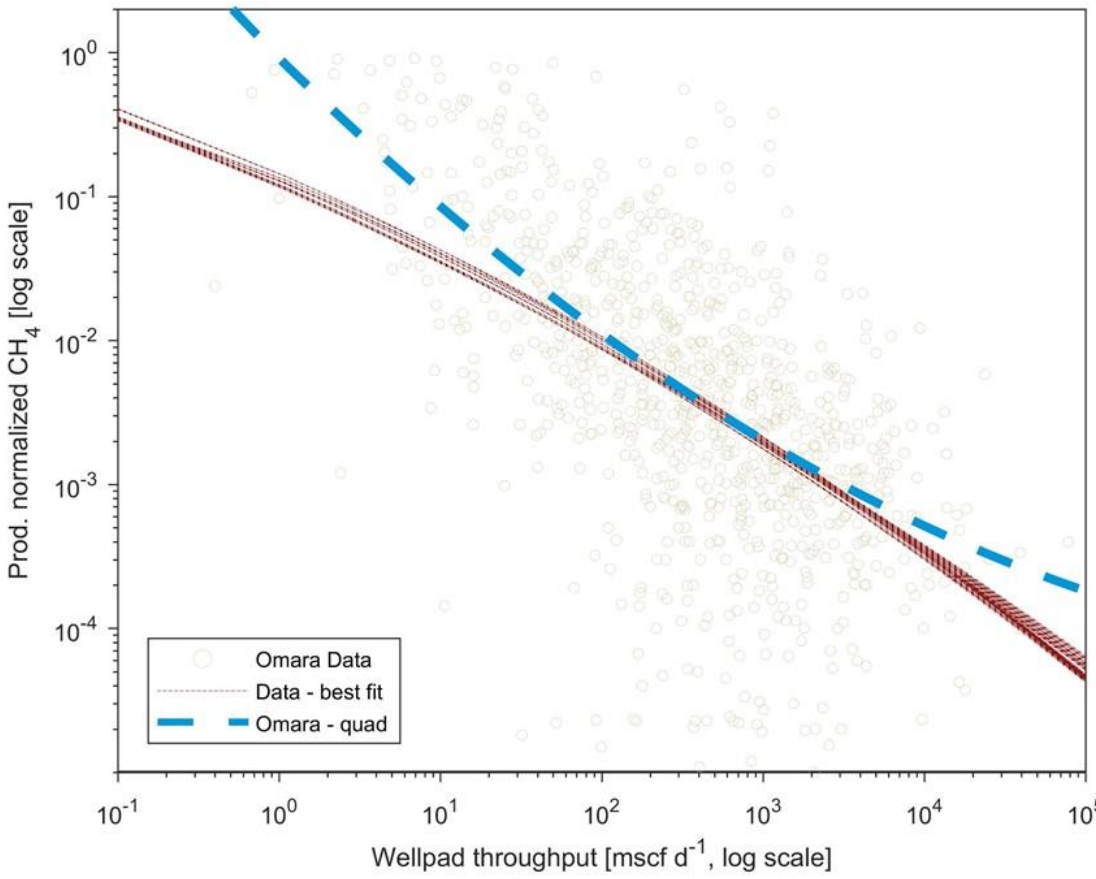

Supplementary Fig. 12: Relationship between site-level productivity and production normalized emissions. Here, we compare production normalized emissions (e.g., fractional loss rate) for this study and Omara et al. [6], respectively (model fits calculated using a quadratic weighted least squares regression).

Site-level studies have also demonstrated higher emissions at liquids-rich sites versus gas-rich sites [49,50]. This is likely due to a higher prevalence of high-emitting equipment like tanks at liquids-rich sites, which is reflected the activity factor parameterization of our model (see **Supplementary Table 14**). We note, however, that this relationship between liquids production and site-level emission in our model is relatively weak, and only provides suggestive evidence (**Supplementary Fig. 13**).

Thus, our model demonstrates key mechanistic trends which have been shown to explain a substantial portion of variability in CH_4 emissions across US basins. However, unlike the site-level data from Omara et al., few component-level measurement studies provide information on

gas and oil production volumes of measured wells and equipment. Our model can replicate Omara's scale dependence through application of a conservation of mass, described in detail in Supplementary Methods 4.

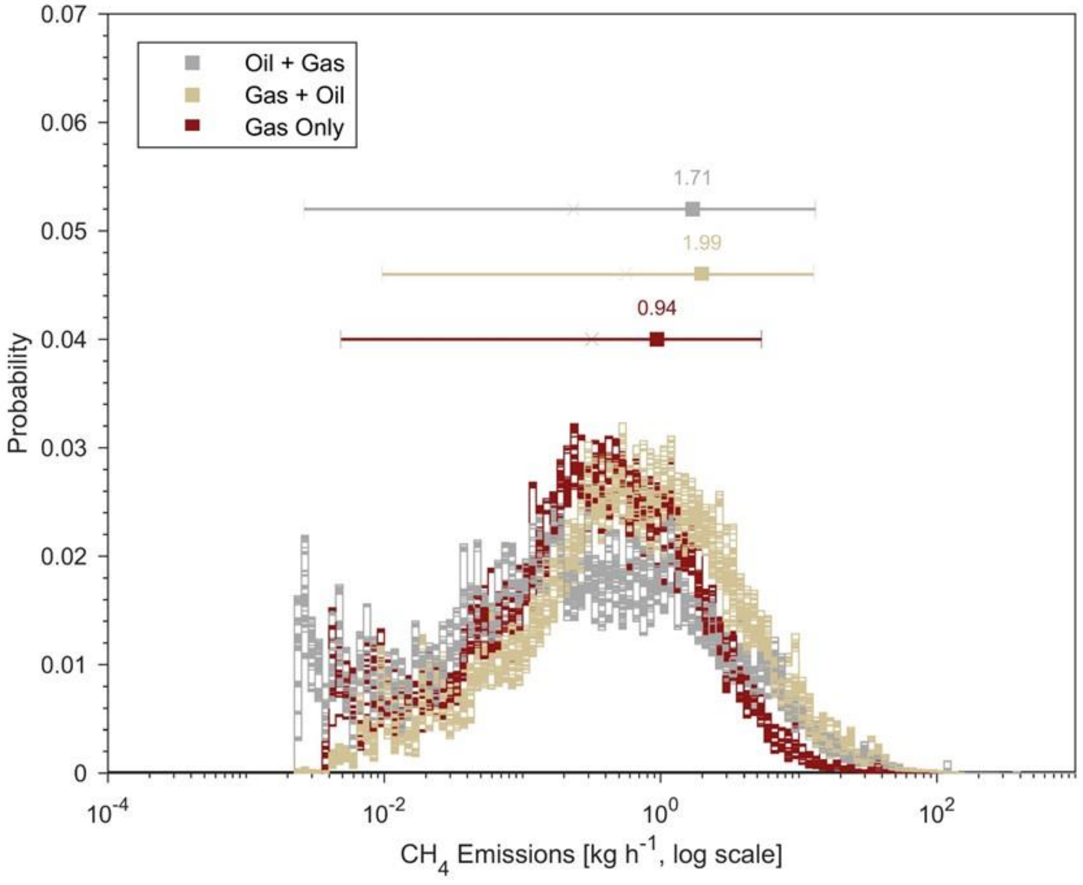

Supplementary Fig. 13: Distributions of emissions rate per site for subsets of results distinguished by production stream. Note that "oil + gas" refers to sites with a GOR < 100 mscf/bbl producing associated gas, and "gas + oil" refers to sites with a GOR > 100 mscf/bbl producing associated liquids. Probability distribution of emissions rate per well-site with the mean (filled square), median (x), and 95% confidence intervals shown above the plots.

Omara et al. [6] also acknowledge that "other factors … such as new state/local regulations or voluntary emissions reduction programs performed by specific operators likely also contribute to basin-to-basin variability". These factors would differ across operators, jurisdictions, and time periods. With a geographically representative set of component-level CH_4 emissions

measurements, hypothetically, it would be possible for our model to capture this variability. However, although we include measurements taken from most major gas producing basins, our limited data set does not mirror exactly the distribution of US gas production. We acknowledge this is likely a source of bias in our model. For example, a large proportion of quantified emissions measurements for storage tanks, and to a lesser extent equipment leaks, are derived from ERG's [12] Fort Worth campaign. Further, our data sample includes measurements published between the period 1993 – 2019. Clearly operating practices have changed during this time period, therefore older data may be less representative of conditions in 2015 (our basis).

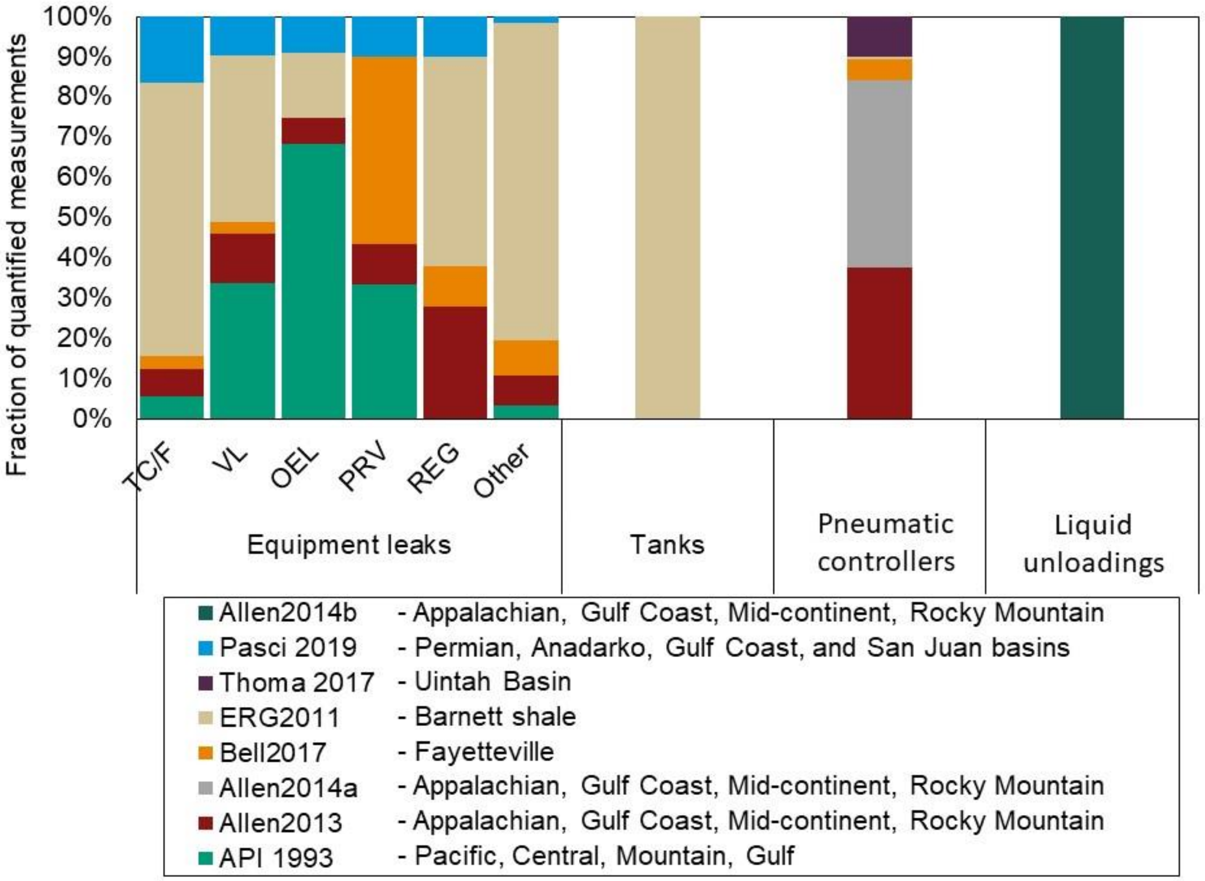

Supplementary Fig. 14: Quantified measurement count by study for key emission sources. The legend is annotated to indicate study measurement locations for all studies included in our dataset [12,15,24–26,34,51]. Equipment leak abbreviations are as follows: TC = threaded connection, F = flange, VL = valve, OEL = open-ended line, PRV = pressure-relief valve, REG = regulator.

When evaluating the utility of our results, we must be clear that the baseline we are comparing to is not a world with perfect information about CH_4 emissions. It is the current GHGI, which is even more data limited. As we elaborate in Supplementary Methods 6, the GHGI emission factors for equipment leaks are based on a regional weighting of “Eastern” and “Western” CH_4 emissions data. However, by regionally segmenting data, this has resulted in very low sample sizes by component for Eastern US gas (e.g., 10 quantified measurements of open-ended lines and 24 measurements of valves). Therefore, while component-level emission factors in the Eastern data (e.g., **Supplementary Fig. 20**) are significantly smaller compared to both this study and the EPA Western US data, it is unclear if this is based on significant differences in operations.

Since these measurements were made, natural gas production in the Eastern US has grown from <5% of US domestic production to ~28% (**Supplementary Fig. 15**).

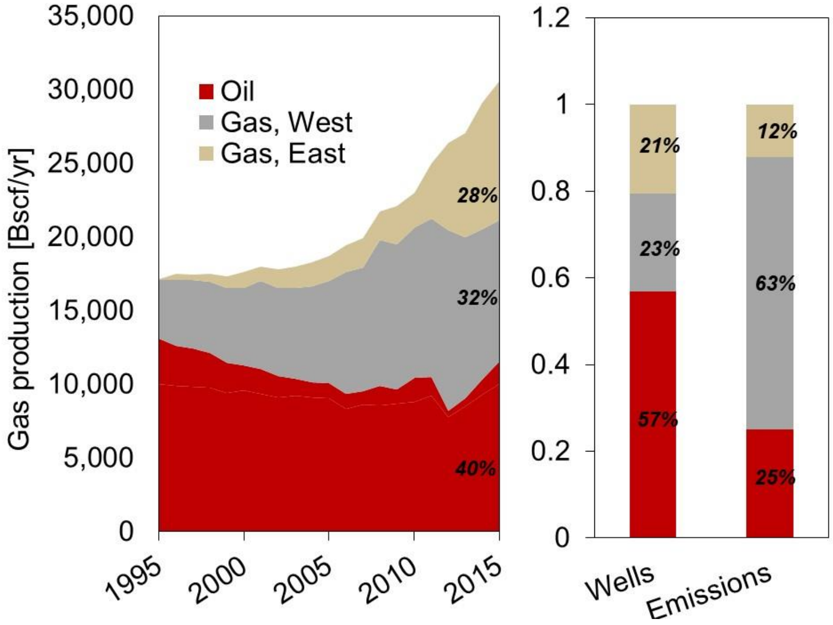

Supplementary Fig. 15: Comparison of gas production, wells, and estimated share of United States production sector CH_4 emissions. Here we compare gas production and well count based on classification schemes use in the Greenhouse Gas Inventory (petroleum systems and natural gas systems in the Western and Eastern United States).

6. Supplementary Methods 6: Production segment CH_4 in the Greenhouse Gas Inventory

The purpose of this section is to explain in detail how the US EPA Greenhouse Gas Inventory (GHGI) estimates production segment CH_4 emissions with the goal of reconstructing the GHGI total emissions values. Unfortunately, for a variety of reasons including data gaps and methodological alignment we cannot fully reconstruct the GHGI total CH_4 emissions estimate. But because our approach uses activity factors very similar to GHGI activity factors (see Supplementary Methods 4) differences in total emissions can be isolated to differences in equipment-level emission factors. Therefore, the focus of this section will be on decomposing GHGI equipment-level emission factors into their underlying data for comparisons with the underlying data of this study.

We begin by investigating the broad categorization of CH_4 emissions within the GHGI production segment, which includes all equipment associated with a well pad and ends prior to centralized gathering and processing facilities (**Supplementary Fig. 1**). We focus our attention on equipment leaks and tank emissions because this is where we observe the biggest discrepancy with our study (**Figure 3,** main text).

Next, we describe the process of reconstructing GHGI equipment-level emission factors from the underlying data sets for equipment leaks, storage tanks, and methane from flares, respectively. All sections contain a similar approach: reviewing the studies underpinning GHGI equipment-level emission factors and their associated datasets, deriving the approach to reconstructing the emission factors (component-level and equipment-level) from the datasets, and verifying our reconstructed emission factor distributions by comparing our averaged data with factors reported in the various studies.

The goal of this reconstruction exercise is to produce decompositions of the data used to produce GHGI equipment-level emission factors (e.g., component-level emission factors, component counts, and fraction of components emitting) for a rigorous comparison with the results of this study.

6.1. Overview of the US GHGI production segment

The US EPA has produced a GHGI since 1997. Each release updates historical and current GHG emissions from the US across all anthropogenic sources [5]. GHGs inventoried include carbon dioxide, methane (CH_4), nitrous oxide, hydrofluorocarbons and others. CH_4 emissions contribute 10% of US anthropogenic GHG emissions (with 100 year GWP = 25, [52]). According to the 2020 GHGI, 2018 CH_4 emissions are estimated to be 25.4 Tg [5]. The largest source of CH_4 emissions was agriculture (enteric fermentation and manure management) contributing 38%, with natural gas and petroleum systems (O&NG) contributing 28% of emissions. The O&NG sectors are contained within the broader energy sector in the GHGI. Within these sectors, CH_4 emissions are categorized by supply chain segment (e.g., production, distribution, etc.). Within the O&NG sectors, CH_4 emissions are calculated at a high resolution with 67 and 45 separate categories, respectively, for the production segment only [5]. Based on the approach of the GHGI, CH_4 emissions have been steadily decreasing since the early 90s (**Supplementary Fig. 16**).

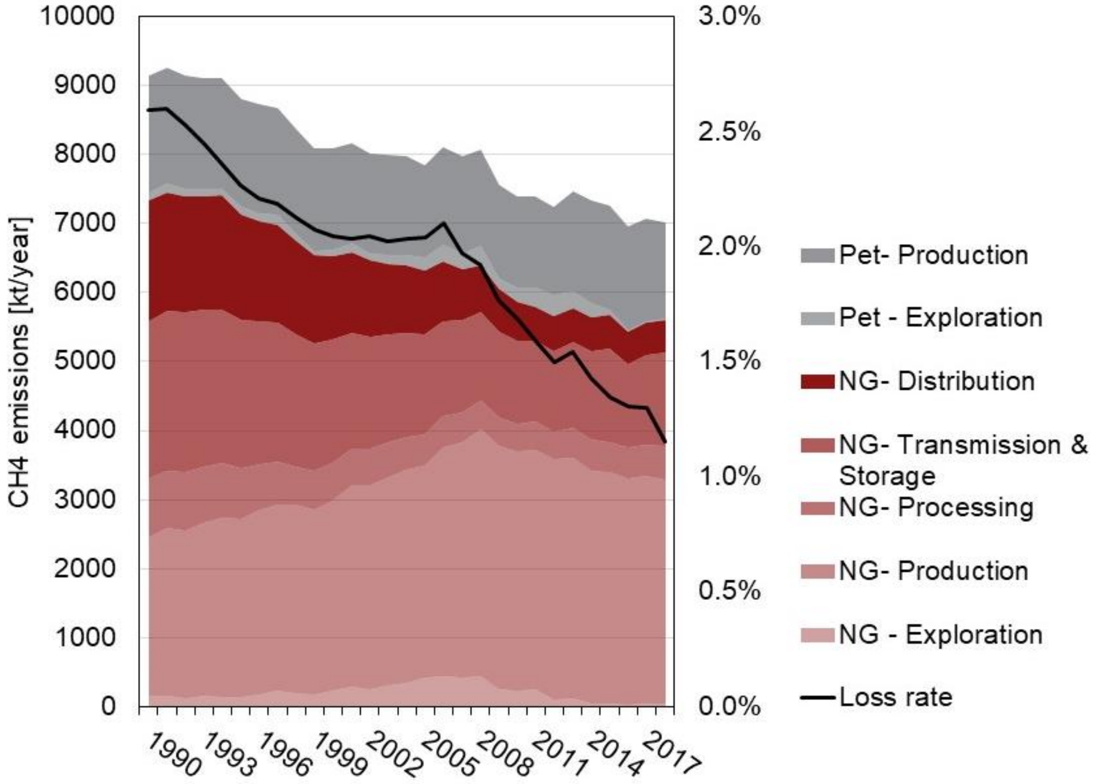

Supplementary Fig. 16: Historic time series of CH_4 emissions according to the Greenhouse Gas Inventory [5]. Loss rate calculated as O&NG production CH_4 emissions divided by gross CH_4 withdrawals (using EIA data and a CH_4 fraction of 82.2%, [39,53]). Note that the loss rate includes all CH_4 emissions from the petroleum system extraction and production segments (i.e., no allocation was performed between natural gas and petroleum systems).

Consistent with the bottom-up approach described in the main text (**Figure 1**), aggregate source-specific emissions in the GHGI are calculated by multiplying emission factors (CH_4 per unit activity, usually number of equipment or in some cases volume throughput) by activity factors. The EPA further distinguishes between potential emissions and net emissions. Potential emissions, the product of emission factors and activity factors, represent uncontrolled emissions. Net emissions represent potential emissions minus emissions reductions, which include voluntary or mandatory emissions reductions (based on EPA analysis of the Natural Gas STAR program and New Source Performance Standards). Given difficulties in obtaining reductions by source category, emissions reductions are typically reported by supply chain segment. However, based on incorporation of new data (described below), these reductions are largely concentrated

in the equipment leaks category. Any comparisons made in this study are with potential emissions before mitigation.

The methodology of the GHGI went relatively unchanged until 2009. However, after increased scrutiny, including a set of field studies led by University of Texas at Austin and the Environmental Defense Fund [24,26,34] the EPA began to revise the methodology. Improvements to the production segment of the O&NG sector have largely relied on data from the Greenhouse Gas Reporting Program (GHGRP). Since 2010 (reported in 2011), through the GHGRP the EPA has mandated that industrial facilities emitting > 25,000 tonne year^{-1} in CO_2eq. terms must report GHG data. Data is available for petroleum and natural gas systems beginning with reporting year 2011 (reported in 2012).

A list of notable (but not exhaustive) revisions to treatment of O&NG emissions in the GHGI includes:

- 2014 GHGI: CH_4 emissions estimates for completions and workovers were revised downwards based on GHGRP data [54]. This downward revision was consistent with field observations reported by Allen et al. [24].
- 2016 GHGI: Using reported counts from GHGRP, pneumatic controllers activity data was adjusted. This resulted in a substantial upward revision in total device counts (nearly double) [55]. Use of the GHGRP data also allowed the EPA to stratify pneumatic controller data by bleed type. This also resulted in a slight upward revision in the average pneumatic controller emission factor. Overall, this resulted in an approximate tripling of estimated CH_4 emissions for pneumatic controllers between 2015 and 2016 (728 Gg to 2672 Gg, [56,57]). Since the 2016 inventory, however, total emissions have been corrected downwards based on further adjustments to component counts (2672 Gg to 1765 Gg in 2017, [57,58]).
- 2017 GHGI: CH_4 emissions estimates for liquids unloadings were updated based on GHGRP data [30]. Prior to this adjustment, Allen et al. [34] had noted that GHGI estimates were already in close agreement with field observations. In the most recent GHGI, 2012 liquids unloadings are still in close agreement with Allen et al. Emissions from liquids unloadings have decreased over time from 263 Gg to 177 Gg in 2018 [5].
  The 2017 GHGI also revised activity and emissions factor data for crude and condensate storage tanks [30].

Despite these modifications incorporating GHGRP data, the best available site-level estimates of production segment emissions suggest that the GHGI still substantially underestimates (see further discussion in main text and [4,6]).

Further, results from our modelling suggest that some data in the GHGI might lead to overestimation of emissions.

- Pneumatic controllers: Since this study uses the same activity factors as the GHGI, differences in emissions must be due to differences in emission factors. Although emission factors in the GHGI have been updated based on GHGRP data, GHGRP emission factors for pneumatic controllers are based on early data from the 1990s [59] comprising a very small sample of measurements (19 data points for intermittent bleed controllers and 41 data points for continuous bleed controllers [60]). Based on our larger sample size of more recent data, we believe that our lower emission factors are more representative of conditions today.
- Methane slip from reciprocating compressors: Methane slip in reciprocating engines is higher in the GHGI, although the overall magnitude in difference is small. The combustion emission factor used in the GHGI for methane slip from reciprocating gas engines is based on a 1991 TRANSDAT dataset published by the Gas Research Institute [61]. The difference compared to our study is probably explained by substantial improvement in engine emissions since publication of that report (based on manufacturer reported specifications for reciprocating gas engines [44]).

We are able to isolate two sources likely contributing to the downward bias of the GHGI: equipment leaks and crude and condensate storage tanks. Our goal with this section is to reconstruct the GHGI published emission factors to understand what drives low emissions estimates in the GHGI.

6.2. Reconstructing GHGI emissions factors for equipment leaks

The construction of equipment-level emission factors for equipment leaks in the GHGI is very similar to the approach of our model (see **Figure 1**, main text), therefore demonstrating how these emission factors were constructed might illuminate sources of bias in the GHGI. Our approach to reconstructing equipment-level emission factors is two-fold. First, we demonstrate how equipment-level emission factors in the inventory are linked to underlying studies and datasets. Second, we review the method of deriving equipment-level emission factors from the

component-level emissions datasets. Finally, we reconstruct component-level emission factors and perform a verification of our reconstructed factors with reported values in the underlying reports.

6.2.1. Sources of emissions factors in the GHGI

The EPA draws from separate reports for constructing equipment-level emission factors, and these reports themselves reference separate component-level datasets. In this section we will begin by outlining the sequence of different studies underpinning the GHGI. We will then describe how these various studies are used to calculate the equipment-level emission factors found in the GHGI. Recall from the previous section how the EPA GHGI constructs separate inventories for petroleum systems and natural gas systems, differentiating gas and oil wells at a cutoff GOR of 100 mscf bbl^{-1} [41]).

The structure of the GHGI is rooted in a suite of studies from the 1990s ([15,16,62]). Some emissions sources have been updated using data from the GHGRP (see previous section), however many sources are still based on this original data. The GHGI cites a 1996 report by the Gas Research Institute ([63], henceforth referred to as the “GRI report”) for natural gas systems and a 1996 calculation workbook by the American Petroleum Institute ([64], henceforth referred to as “API 4638”) for petroleum systems (see the first branch connecting the GHGI to these studies in **Supplementary Fig. 17**).

The GRI report and API 4638 were not measurement campaigns, rather these reports summarized the results of multiple earlier works. The GRI report references API ([15], sites 9-12) for the Western US natural gas system and Star Environmental [16] for the Eastern US natural gas system. API 4638 references data from API ([15], sites 1 – 8) for petroleum systems. Therefore, only two measurement campaigns underlie GHGI equipment leakage: API [15] and the Star Environmental [16] datasets (see the second branch connecting these studies to the GRI report and API 4638 in **Supplementary Fig. 17**).

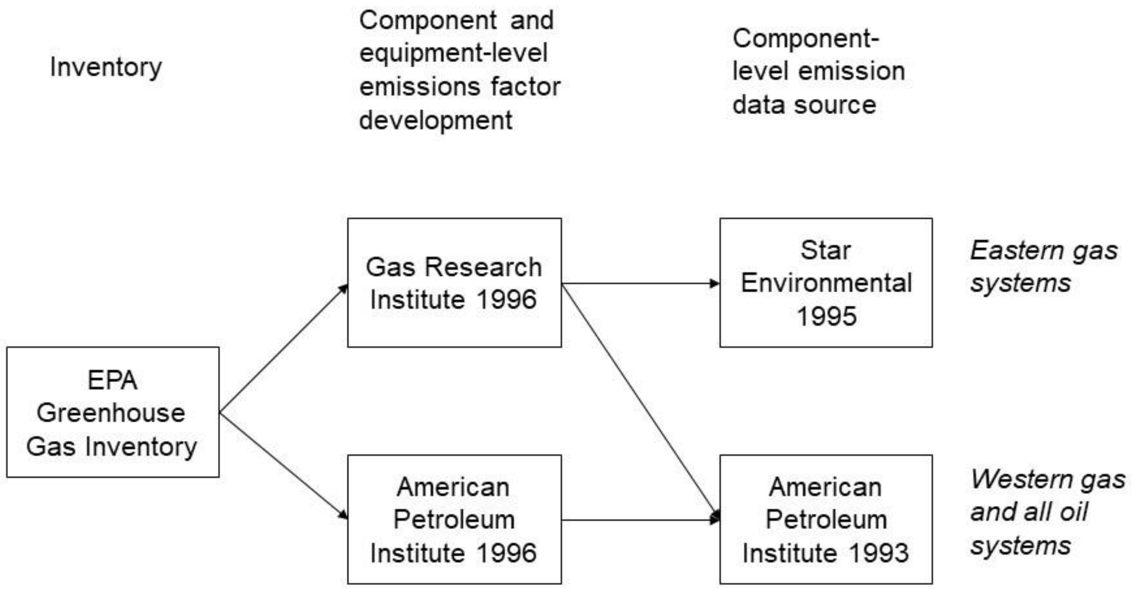

Supplementary Fig. 17: Flow chart representing relationships between various cited studies in the Greenhouse Gas Inventory. The GRI study [63] and API [64] are cited by the Greenhouse Gas Inventory [5] in the development of component and equipment-level emission factors. These two studies cite Star Environmental [16] and API [15] for primary, component-level emissions data**.**

Consistent with the underlying studies, the GHGI further segments equipment-level, equipment leakage emission factors for natural gas systems by region (Western gas versus Eastern gas), and for petroleum systems by product stream (light oil versus heavy oil). For petroleum systems the GHGI maintains this disaggregation by product stream. For natural gas systems, however, the GHGI only reports aggregate emission factors and little documentation is available on how emission factors were constructed from the more detailed data in the underlying studies. To demonstrate the connection between the GRI report and the inventory we attempt an aggregation here.

For onshore natural gas production, the GRI report distinguishes between the Eastern US (Atlantic and Great Lakes regions) and Western US (rest of country) because "regional differences are found to exist" according to the study [63]. Up until the 2015 GHGI [56], emissions were reported in a form broken down by region (Northeast, Mid-Continent, Rocky Mountain, Southwest, West Coast, Gulf Coast, see **Supplementary Table 28**), but they are no longer reported

in this fashion and it is not clear why this practice was stopped. However, by averaging regional emission factors and weighting according to equipment counts – calculated emission factors closely match emission factors applied in the GHGI today (**Supplementary Table 28**).

Supplementary Table 28: Summary of Greenhouse Gas Inventory (GHGI) regional emission factors. EPA GHGI emissions factors are calculated by averaging regional emission factors and weighting according to equipment counts. The last time regional emission factors (and not just averaged totals) were presented in the GHGI was in 2015 [56].

	Equipment counts					
	wells	separator	heater	dehydrator	meter	compressor
North East	72,422	108,357	305	21,277	7,803	153
Mid Continent	74,442	43,996	41,063	14,101	135,554	11,429
Rocky Mountain	10,069	38,333	35,029	10,710	90,791	8,527
South west	22,250	27,624	13,320	6,853	69,076	6,685
West Coast	2,190	1,868	2,559	357	4,569	2,971
Gulf Coast	25,906	45,408	15,458	9,621	82,792	5,590
	Emissions factors [$kgCH_4$ day^{-1}]					
North East	0.15	0.02	0.29	0.45	0.18	5.50
Mid Continent	0.14	0.02	0.29	1.83	0.18	5.39
Rocky Mountain	0.68	2.32	1.10	1.73	1.01	5.09
South west	0.72	2.40	1.13	1.79	1.04	5.26
West Coast	0.82	2.74	1.29	2.04	1.19	6.00
Gulf Coast	0.15	2.63	1.24	1.96	1.14	5.77
	Regional and national average emissions factors [$kgCH_4$ day^{-1}]					
East	0.14	0.02	0.29	0.45	0.18	0.00
West	0.59	2.52	1.19	1.87	1.09	5.50
Total US	0.24	1.06	0.82	1.36	0.74	5.40

The GHGI reports adjustments to emission factors year over year. It is generally not clear in the GHGI documentation why these adjustments were made, but a likely explanation is adjustments to regional shares of well counts and gas composition. Regardless of these adjustments, the equipment-level emission factors have not deviated far from the values published in the GRI report. **Supplementary Fig. 18** compares the emission factors over time (solid lines) from subsequent GHGI reports to the emission factors from the original GRI report dataset (dashed lines) [63].

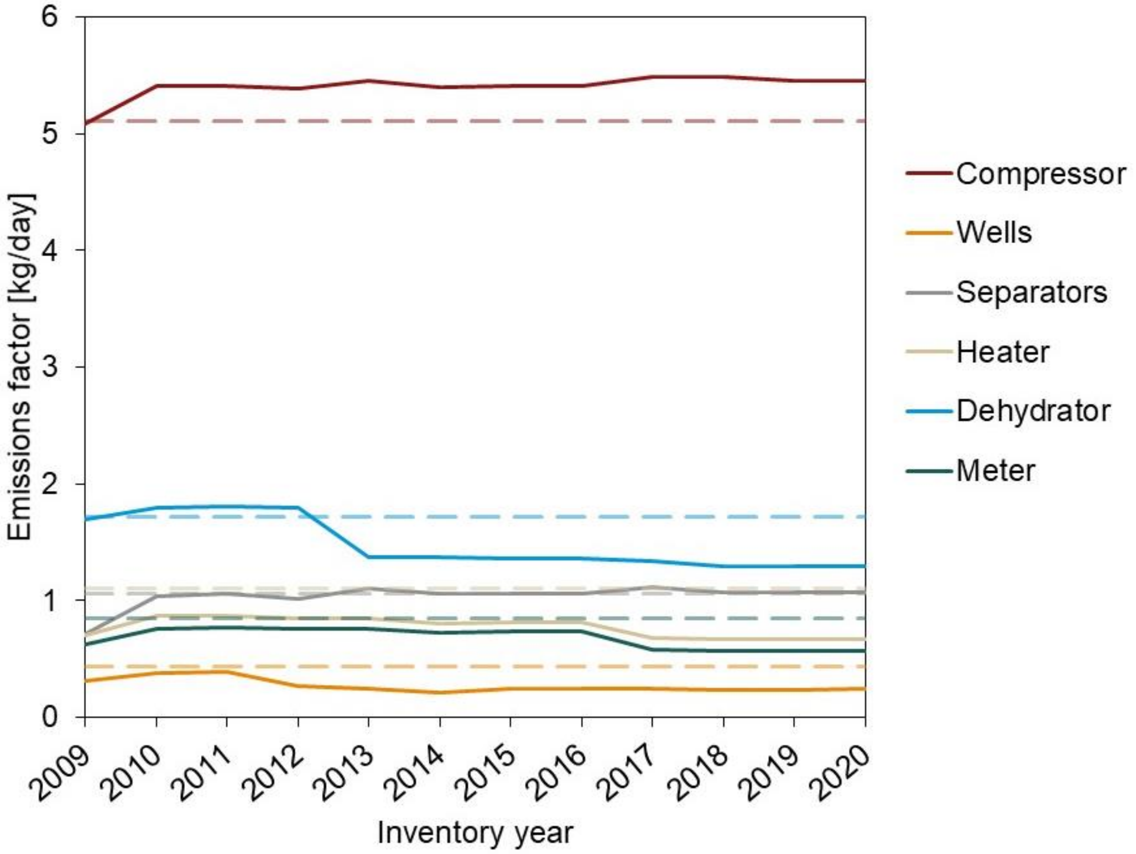

Supplementary Fig. 18: Equipment leakage emission factors in the Greenhouse Gas Inventory (GHGI) have been consistent across historical inventory reports. This figure illustrates year-over-year EPA GHGI emission factors (inventory years 2009 – 2020, corresponding to emission factors for years 2007 – 2018, respectively [5,56,71,72,57,58,65–70]) for NG systems (solid lines). GHGI emission factors closely match those reported in the GRI study [63] (hashed lines) with some deviation. This deviation is not described in published documentation (to the authors knowledge).

6.2.2. Deriving emission factors – General approach

In the previous section, we described how equipment-level emission factors for equipment leakage in the GHGI are based on the GRI report and API 4638 [63,64]. These emission factors are themselves derived from component-level survey data from API [15] and Star Environmental [16] datasets (henceforth referred to as “API 4589” and “Star Environmental” datasets). The approach taken by the GRI report and API 4638 is broadly similar to our approach in that equipment-level emission factors represent a product of component-level data. In the remainder of this section on the GHGI we focus on identifying the key decomposition factors of the equipment-level

emission factors: Component-level emissions measurement data, component counts, and fraction of components emitting. In this section, we will briefly explain this approach, with a focus on the differences with the approach of our study. To begin we will focus on the component-level emissions data.

The general approach taken in the GRI report and API 4638, referred to as the correlation approach, is summarized in the 1995 Protocol document ([17], henceforth referred to as the "EPA Protocol document"). The motivation of the correlation approach is the significant difference in labor between the two types of measurements made in methane surveys: screening measurements and quantified emissions measurements. Ideally, because these techniques result in actual flux measurements, one could gather a large sample of quantified emissions measurements using the bagging or high-flow sampler techniques (see Clearstone [22] for a detailed description of these techniques), however this is often impractical. Instead, screening data are collected according to the EPA Method 21 protocol, usually using a flame ionization device (FID). Screening FID measurements are recorded in units of concentration (ppmv), and correlation equations (correlations of mass flux versus concentration) are used to transform screening data to units of emissions or mass flux (pounds per day).

To fit the correlations, a large sample size of quantified emissions measurements with corresponding screening values is still required. Therefore, the EPA protocol document aggregates quantified emissions measurements across multiple industry segments (refineries, marketing terminals, oil and gas production facilities) and product streams (oil, gas). See Appendix C of the EPA Protocol document for more details [17].

Correlation equations are of the following form, where THC (total hydrocarbon) is the mean emissions rate of total hydrocarbons (lb day^{-1}) and ISV is the instrument screening value (ppmv). a and b are constants defined in **Supplementary Table 29**.

$$\mathrm{THC} = a \cdot (\mathrm{ISV})^{b} \tag{27}$$

A key difference, therefore, between the GHGI-derived emissions factors and the emission factors in our study is the use of correlation equations. Whereas the GHGI has used correlation equations to boost the sample size of underlying measurements our study does not, and instead

relies upon the aggregation of directly quantified leakage volumes reported across multiple studies.

Supplementary Table 29: Correlation equation parameters for various components. Data from the GRI report [63] page 21**.**

Component Type	Correlation equation parameters	
	Constant, *a*	Exponent, *b*
Connector	7.99×10^{-5}	0.735
Flange	2.35×10^{-4}	0.703
Open-ended Line	1.14×10^{-4}	0.704
Pump Seal	2.55×10^{-3}	0.610
Vale	1.21×10^{-4}	0.746
Other	6.98×10^{-4}	0.589

Practical upper limit and lower detection limits of instruments limit the ability of screening approaches. In most studies, the valid range of screening measurements is 10-10,000 ppmv (in some studies the upper limit is 100,000 ppmv). Pegged source and default zero factors were developed to work around these limitations (**Supplementary Table 30**).

If the screening value is reported at the lower limit of the device (10 ppmv), this means that the concentration in the air is at or below the minimum detection limit of the device and the default zero emission factor is used. The default zero emission factor is used instead of a true zero for sources screened at the lower limit of the device to recognize that some small leaks will screen below 10 ppm and should not be counted as truly zero leakage. If the screening concentration is higher than the upper limit of the device, it will report the upper limit value (i.e., 10,000 ppmv in most studies) and the measurement is regarded as pegged. Pegged source factors were developed in the EPA Protocol Document based upon quantified emission measurements. Like the correlation equations, in order to boost sample size, the pegged factors are based upon quantified measurements aggregated across the entire petroleum industry and across all service categories (n = 445, note that this sample includes quantified measurements from both API 4589 [15] and other studies conducted at refineries and marketing terminals, [73,74]). These pegged factors are meant to represent all leaks that result in larger than top-end FID measurements.

A second key difference therefore between the GHGI derived emission factors and our study then is the use of pegged source factors. Whereas our study uses only direct measurements, the GHGI applies an average pegged factor based on previously measured data. We attempt to make a comparison between the GHGI pegged factors and our emissions measurements > 10,000 ppmv later in this section.

Supplementary Table 30: Default zero and pegged emissions factors for various components. Data from the GRI report [63].

Component type	Default Zero Factor [lb THC/day]	Pegged Factor [lb THC/day]
Connector	3.97 x 10-4	1.48
Flange	1.64 x 10-5	4.50
Open-ended Line	1.06 x 10-4	1.59
Pump Seal	1.27 x 10-3	3.92
Vale	4.13 x 10-4	3.39
Other	2.12 x 10-4	3.86

A final difference to note between our study and the GHGI is the use of population emission factors versus leaker emission factors (these approaches are described in greater detail in the EPA Protocol document [17]). Our study takes a leaker emission factor approach, with units of kilogram per day per number of emitting components. As discussed in Supplementary Methods 4, screening data is used to define leakers and non-leakers according to a pre-defined threshold (500 ppmv, or optical gas imaging identification). When calculating total emissions, a random subset of components are designated as leakers, with a probability defined by the fraction of components emitting found in field campaigns that include both emissions measurements and component counts.

This contrasts with the population emission factor approach taken by the GHGI. The population emission factor *(*units of kilogram per day per individual source*)* is the average across the population of components, including both leakers and non-leakers. To calculate population emission factors, we divide the total leakage across all components by the total number of potential leak sources. Because the population emission factor represents the average emissions

across all components, explicitly specifying the fraction leaking value is not necessary. Rather, population emission factors simply are multiplied by component counts.

The general equation for population emission factors is as follows,

$$EF_{pop} = \frac{0.454 \cdot Y_{C1} \cdot \left(n_{nl} \cdot DZF + \sum_{i=1}^{n_l} a \cdot (ISV)^b + n_e \cdot PSF\right)}{n_{nl} + n_l + n_e} \quad (28)$$

Here, screening data (ISV , units of ppmv) is converted to mass of total hydrocarbon using correlation equations, default zero factors (DZF), and pegged source factors (PSF) (see **Supplementary Table 29** and **30**). In converting from mass of total hydrocarbon to mass CH_4, we apply mass fractions (Y_{C1}). Mass of CH_4 is converted to a population emissions factor (EF_{pop}, units lb CH_4 component^{-1}d^{-1}) by dividing by total components screened which is the sum of components screened by non-emitting (n_{nl}), the sum of components emitting (n_l), and the sum of components pegged (n_e).

We don't examine in detail the statistical properties of the correlation equation approach, but note that direct quantitative measurements (for example, using a high flow sampler) should be preferred to the correlation equation approach. Caution should especially be used given the treatment of large emitters using pegged factors, given the likely importance of the top 5% (super-emitters).

It is important to note here that API 4589 and Star Environmental themselves report component-level emission factors. However, the GRI report and API 4638 do not use these directly reported values. Rather, the GRI report and API 4638 assemble the data collected in these studies and conduct a slightly modified analysis. We also note that some specificity in component-level data was lost when measurements from API 4589 [15] and Star Environmental [16] was applied to the GRI report [63] and API 4638 [64]. Although 9-component and 7-component classification schemes were used in API 4589 and Star Environmental, respectively (see detail in Supplementary Methods 7), this detail was lost when the data was applied in the GRI report and API 4638 (reduced to a 4-component classification scheme). This is likely because correlation equations and pegged source factors are only available for a limited number of components (see **Supplementary Table 29** and **30**). However, given that the majority of components (and their emissions) for equipment

leaks can be attributed to connectors, valves, and open-ended lines, this issue is not of primary concern.

For additional details on API 4589 and the Star Environmental reports see Supplementary Methods 7.

6.2.3. Summary of EPA GHGI emission factors

Before reconstructing emission factor distributions, we reconstruct average emission factors from the GHGI data. We reconstruct the average equipment-level emission factors calculated in the GRI report and API 4698 [63,64], and subsequently used by the EPA GHGI by summing the emission factors of individual components according to estimated counts of components per piece of equipment. (**Supplementary Table 31**). For natural gas systems, the component-level emission factors and component counts referenced in **Supplementary Table 31** are directly as reported (with the appropriate unit conversion) in the GRI report [63]. For petroleum systems, the GHGI references API publication 4638 [64] for component-level emission factors. Component counts are given directly in the GHGI data source documentation, based on the "consensus of an industry review panel" [5].

Supplementary Table 31: Summary of Greenhouse Gas Inventory equipment-level emission factors and underlying component-level data. Equipment-level emission factors are calculated as the product of component-level emission factors (population emissions factors, or average across both leaking and non-leaking components) multiplied by the corresponding component count, summed across all components. The underlying studies supporting the GHGI report separate equipment-level emission factors for Western US gas, Eastern US gas, light oil, and heavy oil systems. Note that the numbers for Eastern Gas Wells are as reported in the GRI report, therefore are slightly different from what is reported in the Star Environmental study.

		Western US gas systems			Eastern US gas systems			Light oil systems			Heavy oil systems		
		Component EF kg comp^{-1}d^{-1}	Comp count	Equipment EF kg eq^{-1}d^{-1}	Component EF kg comp^{-1}d^{-11}	Comp count	Equipment EF kg eq^{-1}d^{-1}	Component EF kg comp^{-1}d^{-1}	Comp count	Equipment EF kg eq^{-1}d^{-1}	Component EF kg comp^{-1}d^{-1}	Comp count	Equipment EF kg eq^{-1}d^{-1}
Gas or oil well	Valve	0.044	11	0.701	0.010	8	0.137	0.036	5	0.320	1.88E-04	5	0.002
	Flange							0.002	10		8.98E-06	10	
	Connection	0.006	36		0.001	38		0.003	4		1.71E-04	4	
	OEL	0.011	1		0.022	0.5							
	Polished rod							?	1		?	1	
Separators	Valve	0.044	34	2.346	0.010	1	0.017	0.036	6	0.267	1.88E-04	6	0.003
	Flange							0.002	12		8.98E-06	12	
	Connection	0.006	106		0.001	6		0.003	10		1.71E-04	10	
	OEL	0.011	6										
	PRV	0.070	2										
Heaters	Valve	0.044	14	1.110	0.010	14	0.273	0.036	8	0.369			
	Flange							0.002	12				
	Connection	0.006	65		0.001	65		0.003	20				
	OEL	0.011	2		0.022	2							
	PRV	0.070	1		0.015	1							
Dehydrators	Valve	0.044	24	1.752	0.010	24	0.418						
	Connection	0.006	90		0.001	90							
	OEL	0.011	2		0.022	2							
	PRV	0.070	2		0.015	2							
Meter/piping	Valve	0.044	14	1.017	0.010	12	0.173						
	Connection	0.006	51		0.001	45							
	OEL	0.011	1										
	PRV	0.070	1										
Gathering compressor	Valve	0.044	73	5.149	0.010	12	0.233						
	Connection	0.006	179		0.001	57							
	OEL	0.011	3		0.022	2							
	PRV	0.070	4										
	comp seal	0.125	4										
Header	Valve							0.036	5	0.209	1.88E-04	5	0.002
	Flange							0.002	10		8.98E-06	10	
	Connector							0.003	4		1.71E-04	4	

6.2.4. Comparing re-analyzed component-level emission factors

Using the data presented from the underlying studies in the previous section, we can develop component-level emission distributions for comparison with distributions generated in this study. Here, we first describe how these distributions were developed. Next, using these component-level emissions data, we attempt to re-calculate the component-level emission factors reported by the GHGI's underlying studies.

We first analyze the screening data in API 4589 [15] and Star Environmental [16] and follow the methods outlined in prior subsections of Supplementary Methods 6. In API 4589, we scanned and tabulated screening concentrations from Appendix C. Unfortunately, it was not possible to re-derive the component-level emission factors in the Star Environmental dataset. This was for two reasons. First, in the Star Environmental emissions quantification data (provided in Appendix F, [16]), information is not provided on components measured. Therefore, quantified leaks cannot be connected to the screening values contained in Appendix E. Second, the Eastern dataset does not report how they assigned leak volumes to the 81 instrument readings > 10,000 ppmv which were not quantified with the Hi Flow sampler. Therefore, the remainder of this section will focus on re-generated emission factors from API 4589.

Several issues arise in regenerating component-level emission factors from the API 4589 data. First, it is unclear which correlation equations, pegged source factors, and default zero factors were applied for pressure relief valves and compressor seals. No equation and factors matching these component names are available in the EPA Protocol Document [17] or GHGI. To generate a first order estimate, we apply equations and factors under the "other" category. Second, classification of "threaded connections" and "flanges" is not consistent across the GHGI's underlying studies. After presenting our results, we will investigate uncertainty in connector/flange classification.

After completing our re-analysis of the API 4589 data, we can compare our re-analyzed results with reported emission factors in the GHGI. For both natural gas systems and petroleum systems there is significant variability in alignment with our computed component-level emission factors based on the same data GHGI cites (**Supplementary Table 32**). Valves compare well, with absolute variation ≤6% across sources. For other component classes, although emission factors match the

correct order of magnitude, alignment is less precise. There are several possibilities for the source of misalignment. In general, although GRI and the EPA Protocol Document recalculate emission factors based upon the original API 4589 dataset, only a limited amount of information is provided on how the data is used, therefore, it was not possible to perfectly reconstruct emission factors.

Supplementary Table 32: Reconstructed Greenhouse Gas Inventory component-level emission factors. We recalculated the Greenhouse Gas Inventory component-level emission factors (based upon those reported in the GRI Report [63] and the EPA Protocol document [17]) and compared with the original values. The reconstructed emission factors match the reported values within the correct order of magnitude, which is close considering discrepancies with emission factors from this study's model.

Component	Reported emission factor [$kgCH_4$ component^{-1}d^{-1}]	Reconstructed emission factor [$kgCH_4$ component^{-1}d^{-1}]	Percent difference [%]
Gas production sites[1]			
Connector	6.01E-03	4.71E-03	-22%
Valve	4.40E-02	4.52E-02	3%
Open-ended line	1.13E-02	1.25E-02	10%
Pressure relief	7.02E-02	2.57E-02	-63%
Compressor Seal	1.25E-01	8.58E-02	-31%
Light oil sites[2]			
Connector[3]	3.08E-03	2.51E-03	-19%
Flange[3]	1.62E-03	2.97E-03	84%
Valve	3.67E-02	3.41E-02	-7%
Open-ended line	2.06E-02	3.30E-02	61%
Heavy oil sites[2]			
Connector[3]	1.70E-04	1.11E-04	-35%
Flange[3]	8.82E-06	7.37E-06	-16%
Valve	1.90E-04	1.91E-04	1%
Open-ended line	3.17E-03	3.27E-03	3%

[1]Reported emissions factor from Hummel et al. (1996) [63]
[2]Reported emissions factor from EPA (1995) Protocol document [17]
[3]Recalculated iteratively assigning connectors and flanges

As it was mentioned, for natural gas system emission factors the classification of "threaded connections" and "flanges" is not consistent across studies. In the EPA Protocol document, separate correlation equations and pegged source factors are provided for these two components. However, in the API 4589 source data, both "threaded connections" and "flanges" are classified

as connectors. In the GRI report, they describe how a "combined component emission factor for flanges/connectors was calculated" but little information is given on how this is accomplished [63]. The EPA Protocol Document derives an emission factor for flanges by assuming (based on gas plant measurements in API 4615, [62]) that 71% of all "connectors" measured were threaded connections while the remainder were flanges. Unfortunately, neither the EPA Protocol Document or API 4615 specify which measurements are assigned as connectors and which are assigned as flanges.

To test the sensitivity of the emission factor to how the measurements are assigned, we iteratively re-shuffle the measurements and assign connectors and flanges using the EPA Protocol document's 71% share of "threaded connections". The results demonstrate a high sensitivity to re-shuffling for light oil but not heavy oil (**Supplementary Fig. 19**). This is because for light oil, 356 leaks were measured at connectors, while for heavy oil only 4 leaks were measured (with relatively small variations in emissions rates). It should be noted that although API 4615 notes how many leakers were classified as connectors versus flanges, they do not specify the distribution of non-leakers. We assume a 50% split in our main analysis.

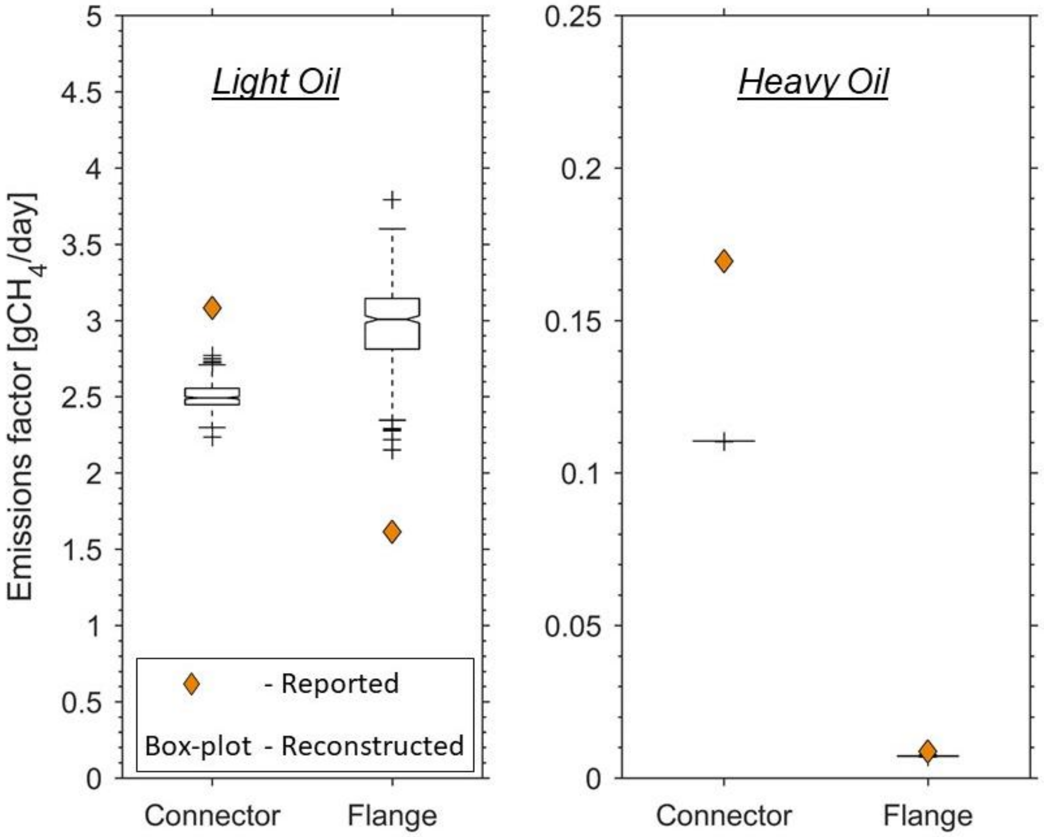

Supplementary Fig. 19: Comparisons of the range of reconstructed component-level connector and flange emission factors with reported emission factors. Box plots represent the range of possible emissions factors determined by reshuffling measurements and randomly assigning connectors and flanges for light oil (500 realizations) and heavy oil (20 realizations). For each box, the median is the central notch and the 25th and 75th percentiles, respectively, are the bottom and top edges of the box. Reported emission factors (based upon the GRI report) are represented by orange diamonds**.**

6.2.5. Comparison of emission factors cited by the GHGI with emission factors generated in this study

After digitization and re-engineering of the GHGI methods, we can compare the distributions of the resulting component-level estimates with our dataset. In this section we first compare the distributions. As it is noted in the main text, a key result is the striking difference in component level emissions measurements. This could either be the result of fundamentally different O&NG systems, or it could be the result of under-sampling or the use of constant pegged source factors

to represent the upper tail of emission values. We investigate this question in this section with an iterative Monte Carlo test of sample size.

As described in more detail in Supplementary Methods 4, our dataset is split at a threshold screening concentration of 10,000 ppmv. This was done because different screening technologies will sample different parts of the emissions distribution. It must be taken care that emissions frequency and emissions measurements are not confused between technologies.

We also compare our dataset separately with data underlying the GRI report's (i) Western and petroleum systems and (ii) Eastern systems. For Eastern systems, low emitters (< 10,000 ppmv) are based upon the Star Environmental [16] dataset combined with EPA Protocol Document correlation equations [17]. Higher emitters (> 10,000 ppmv) are based solely upon High Flow sampler measurements made for the Star Environmental study. For Western and petroleum systems, low emitters are based upon API 4589 [15] combined with the EPA Protocol Document correlation equations. High emitters are based upon pegged source factors developed in the EPA Protocol Document [17]. For this section, rather than simply comparing with the pegged source factor, we present the full distribution of emissions measurements used to generate the pegged source factor. Recall that pegged source factors were developed based on quantified emissions measurements (High Flow sampler) from refinery, marketing terminal, and oil and gas production facilities (measurements digitized from the EPA Protocol document Appendix C Attachment 2, [17]). It should be noted that quantified emissions measurements from API 4589 (production segment only) are also contained in our study. On average, our dataset has much larger population-level component leakage estimate than both the Western and Eastern datasets (**Supplementary Fig. 20**). This issue is discussed in greater detail in the main manuscript.

Supplementary Fig. 21 contains a component-by-component comparison. Recall that we were not able to do a component-by-component breakdown from the Star Environmental Eastern dataset, therefore data here is only presented for API 4589 (Western gas systems and petroleum systems). Here, we do not present the underlying data for pegged source factors. The pegged source factors in API 4589 probability density functions can be easily spotted as the tall bars near the upper end of the emissions magnitudes.

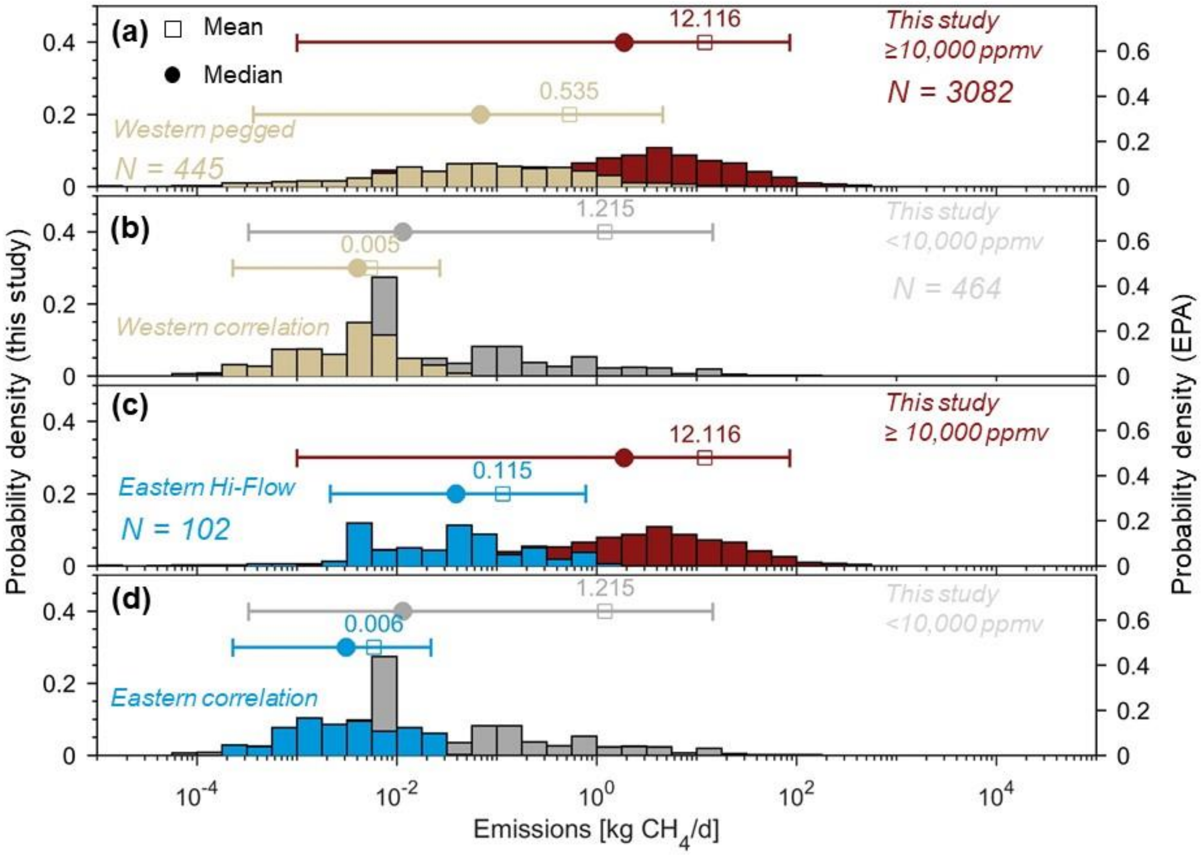

Supplementary Fig. 20: Comparison of component-level quantified emission datasets between this study and underlying data sources for the Greenhouse Gas Inventory. Separate comparisons are made between the Western US and petroleum data set (API 4589 and EPA protocol document) [15,17](**a** and **b**) and the Eastern US data set (Star Environmental) [16] (**c** and **d**). Western and Eastern datasets are disaggregated into component-level emissions underlying pegged factors for comparison with our dataset ≥ 10,000 ppmv (**a** and **c**) and emissions generated from correlation equations (corr.) are compared with our dataset < 10,000 ppmv (**b** and **d**). Rather than comparing with the pegged source factors (a single number), in panel (**a**) we compare our dataset with the underlying emissions measurements used in developing the pegged source factors (digitized from EPA Protocol document Appendix C Attachment 2, [17]). Note that quantified measurements from API 4589 [15] are present in both our dataset and the EPA Protocol document pegged source factor dataset. Horizontal bars span the 5th and 95th percentile, the hollow box is the median, and the filled circle is the mean. Note that the histogram scale for this study's dataset is on the left-hand side of the plots, and the scale for the GHGI datasets is on the right hand side of the plots. Note the log scale of emissions with the implication that discrepancies span 1-3 orders of magnitude.

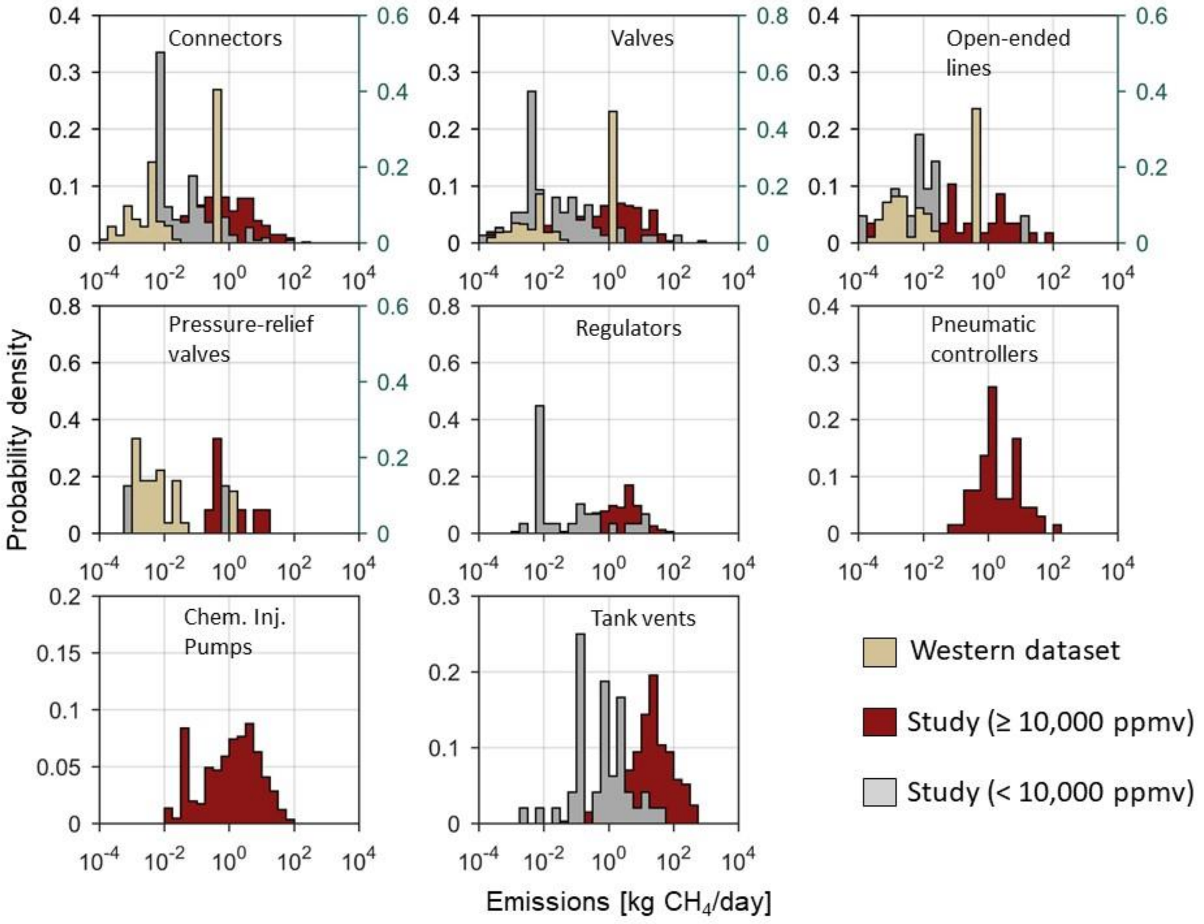

Supplementary Fig. 21: Disaggregated probability distributions of component-level emissions. Here, we only compare the Western US (API 4589) dataset with our dataset. Note that the histogram scale for this study's dataset is on the left hand side of the plots, and the scale for the API 4589 is on the right hand side of the plots. Note the log scale of emissions with the implication that discrepancies span 1-3 orders of magnitude.

Both the pegged source factors generated in the EPA Protocol document and high-flow sampler data in the Eastern gas dataset have smaller sample sizes compared to our dataset (n = 445 and n = 102, respectively, versus n = 3082 >10,000 ppmv in our dataset). Could an insufficient sample size explain the difference in magnitude of component-level emission factors between our studies? As described in detail in Brandt et al. [22], small sample sizes can miss low-frequency super-emitters, resulting in low emissions. The lower range of the sample mean increases as sample size increases and as super-emitters are increasingly captured. To test this this theory of insufficient sample size (or, in other words, if EPA's small averages are due to a small sample which misses super-emitters), we perform a Monte Carlo exercise.

First, we filter our dataset into connectors, valves, and open-ended lines (because these are clearly marked in our datasets and [25]). Next, we resample (without replacement) subsets of different sizes of our full dataset. The mean of our sample set is calculated for each subset. In this way, we test how sample size affects component-level emission factors (**Supplementary Fig. 22**). This exercise is performed 500 times (multiple colored lines in **Supplementary Fig. 22**). By comparing the probability envelopes generated with this resampling exercise with emissions factors from the EPA, we can estimate the likelihood of sample size explaining the discrepancy.

The small sample sizes of Star Environmental (65 connections, 28 valves, 7 open-ended lines) might have resulted in finding lower component-level emission factors compared to a larger dataset. However, when we compare actual sample sizes and mean emissions rates from Star Environmental (orange diamonds in **Supplementary Fig. 22**) to our envelopes generated via Monte Carlo resampling, we see that the Star Environmental mean emissions rates are very low even accounting for their small sample size. In fact, it is very difficult to recreate emission factors this low by sampling from our dataset. Further, even though pegged source factors in the EPA Protocol document are sourced from a larger dataset, the emission factors still fall outside of the expected range demonstrated by the 95% uncertainty envelope generated with resampling from our dataset.

We can think of three options to explain this discrepancy: (1) there is sampling bias in the original collection process; (2) the two populations being sampled (original 1990s studies and our studies) are fundamentally different due to differences in equipment type, age, or complexity (e.g., most O&NG is now produced from unconventional shale formations, where it wasn’t in the original GRI study); or (3) there is a time trend in emissions rates between the 1990s and today.

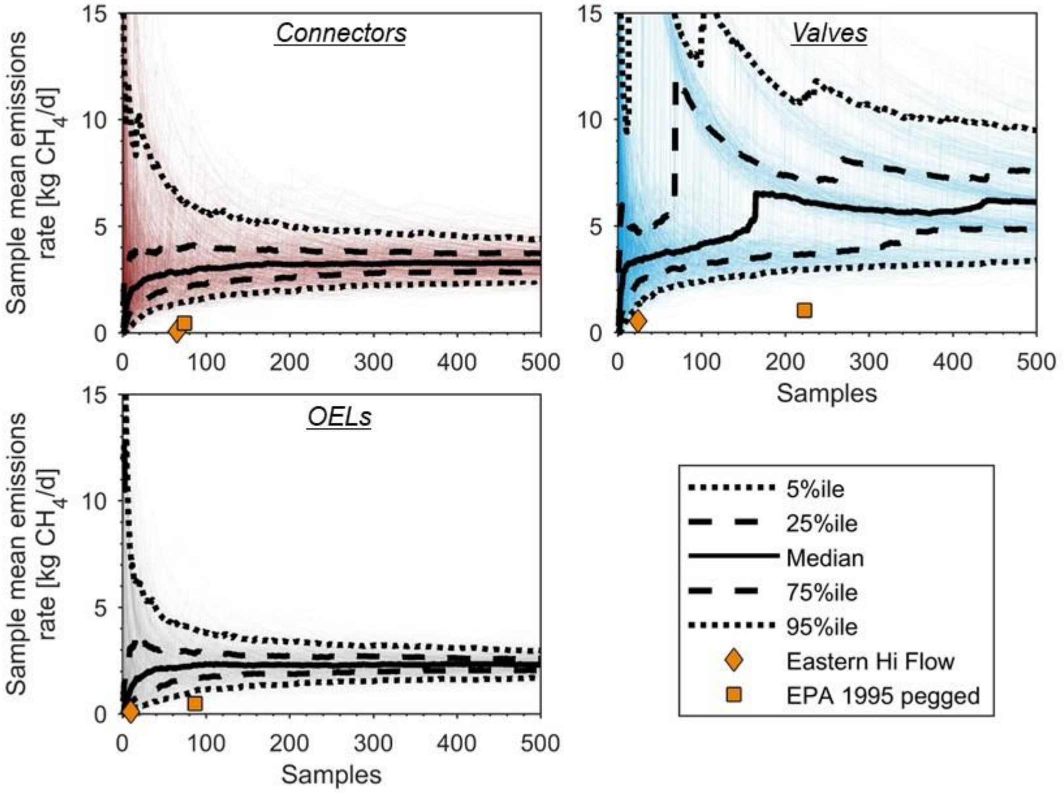

Supplementary Fig. 22: Realizations of sample mean emission rate versus sample size for various components derived by resampling our modern dataset. By comparing the probability envelopes generated with this resampling exercise with emission factors from the EPA [16,17], we can estimate the likelihood of sample size explaining the discrepancy. This exercise demonstrates that the odds of EPA emission factors coming from a similar distribution as ours are very small.

6.2.6. Emission factor decompositions

In the main text we presented a decomposition of equipment leakage emission factors for gas wells in Western natural gas systems. Below we present additional decompositions for both Eastern and Western systems and various equipment (**Supplementary Fig. 23 –31**).

Recall that, for this study, equipment-level emission factors are the superposition of large emitters (>10,000 ppmv) and small emitters (500-10,000 ppmv), each defined by a separate set of measured leaks and fraction of components emitting (see discussion in Supplementary Methods 4). Similarly, for EPA GHGI construction of equipment-level emission factors, large

emitters are characterized by pegged source factors and small emitters are characterized by correlation equations. For illustrative purposes, and because large emitters (>10,000 ppmv) constitute the majority of total emissions, our decomposition plots do not include component-level emission factors and fraction of components emitting for small emitters. Thus, total equipment level emission factors will not calculate exactly as the product of the constituent parts. For example, for heavy oil wellheads in **Supplementary Fig. 25**, the fraction of components emitting is zero. This is because in the API 4589 [15] survey, no components were found emitting at heavy oil wellheads at >10,000 ppmv. However, the equipment level emission factor is still non-zero because components were found emitting at 500-10,000 ppmv.

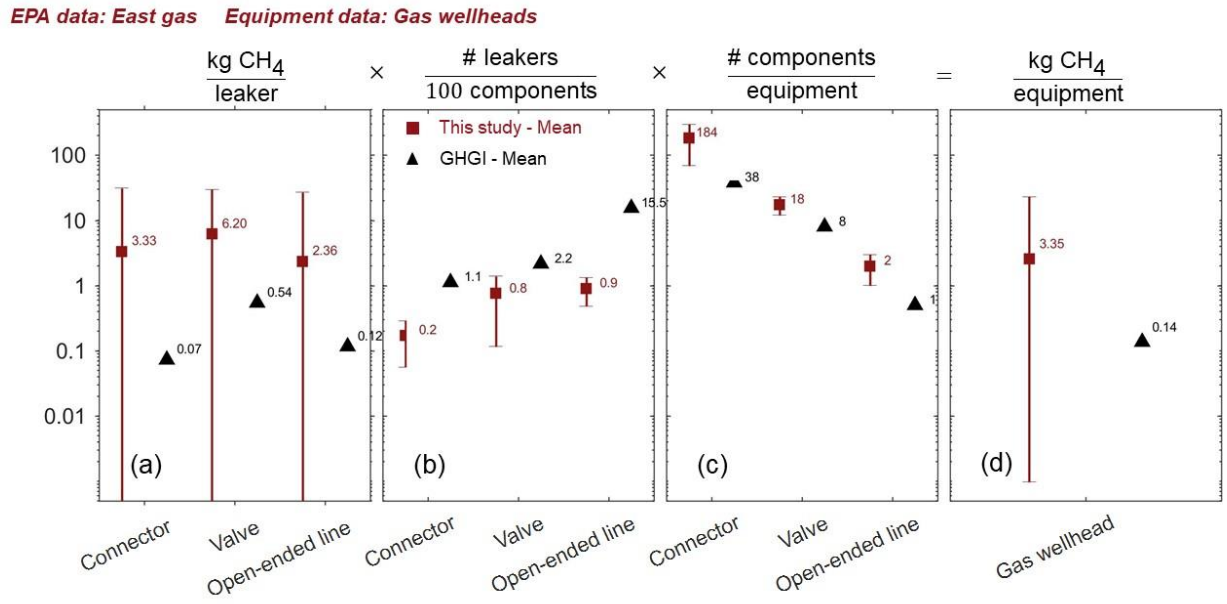

Supplementary Fig. 23: Decomposition of GHGI equipment-level emission factor for gas wellheads. Equipment-level emission factors are decomposed into constituent parts. Equipment-level emission factors for gas systems are a function of data for both Western systems (API 4589, [62]) and Eastern gas systems (Star Environmental, [16]). Here, we only show constituent data for Eastern gas systems. Error bars reflect the 95% confidence interval based on the 2.5 and 97.5 percentile values extracted from the empirical distributions and filled squares and triangles represent the mean.

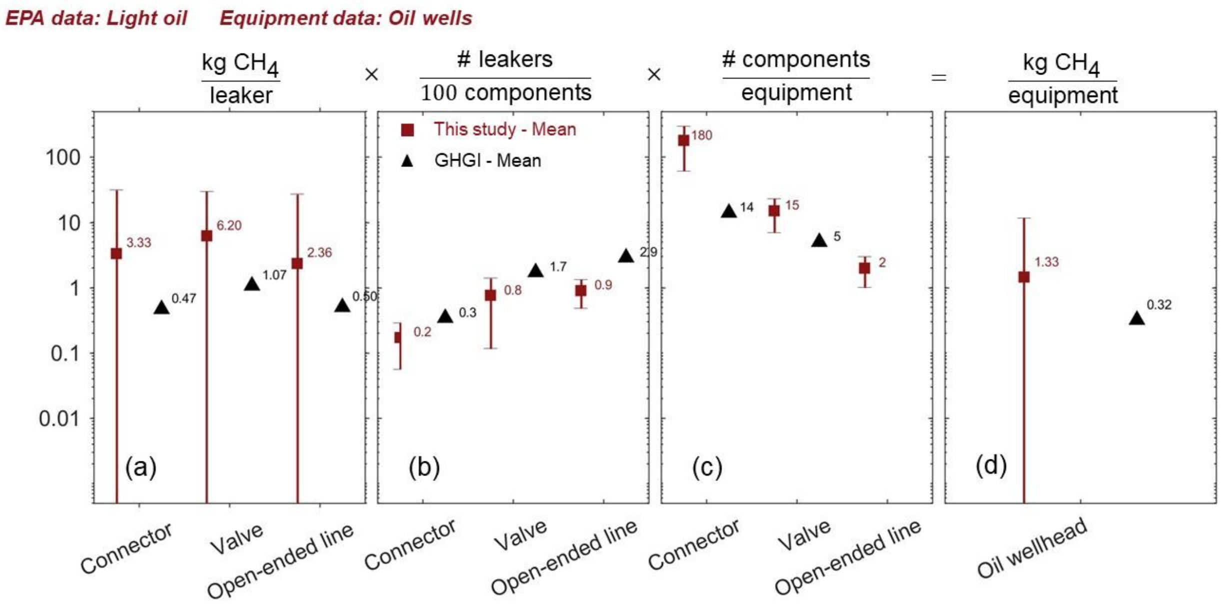

Supplementary Fig. 24: Decomposition of GHGI equipment-level emission factor for oil wellheads. Equipment-level emission factors are decomposed into constituent parts. Equipment-level emission factors for oil systems are a function of data for both light oil systems and heavy oil systems (both from API 4589, [62]). Here, we only show constituent data for light oil systems. Error bars reflect the 95% confidence interval based on the 2.5 and 97.5 percentile values extracted from the empirical distributions and filled squares and triangles represent the mean.

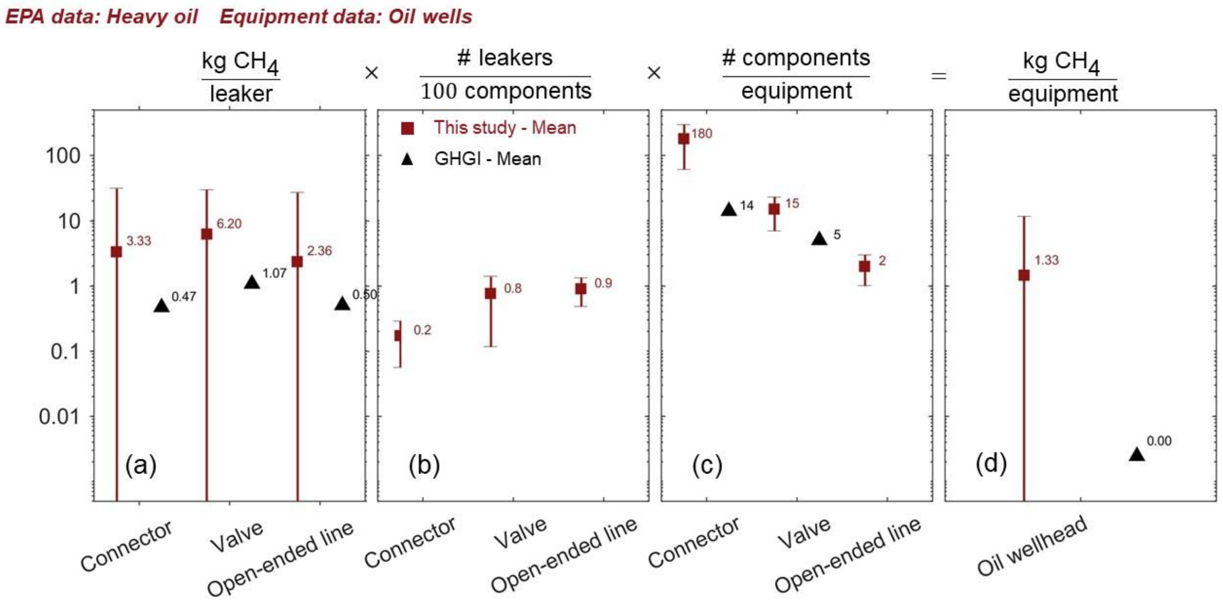

Supplementary Fig. 25: Decomposition of GHGI equipment-level emission factor for oil wellheads. Equipment-level emission factors are decomposed into constituent parts. Equipment-level emission factors for oil

systems are a function of data for both light oil systems and heavy oil systems (both from API 4589, [62]). Here, we only show constituent data for heavy oil systems. Error bars reflect the 95% confidence interval based on the 2.5 and 97.5 percentile values extracted from the empirical distributions and filled squares and triangles represent the mean.

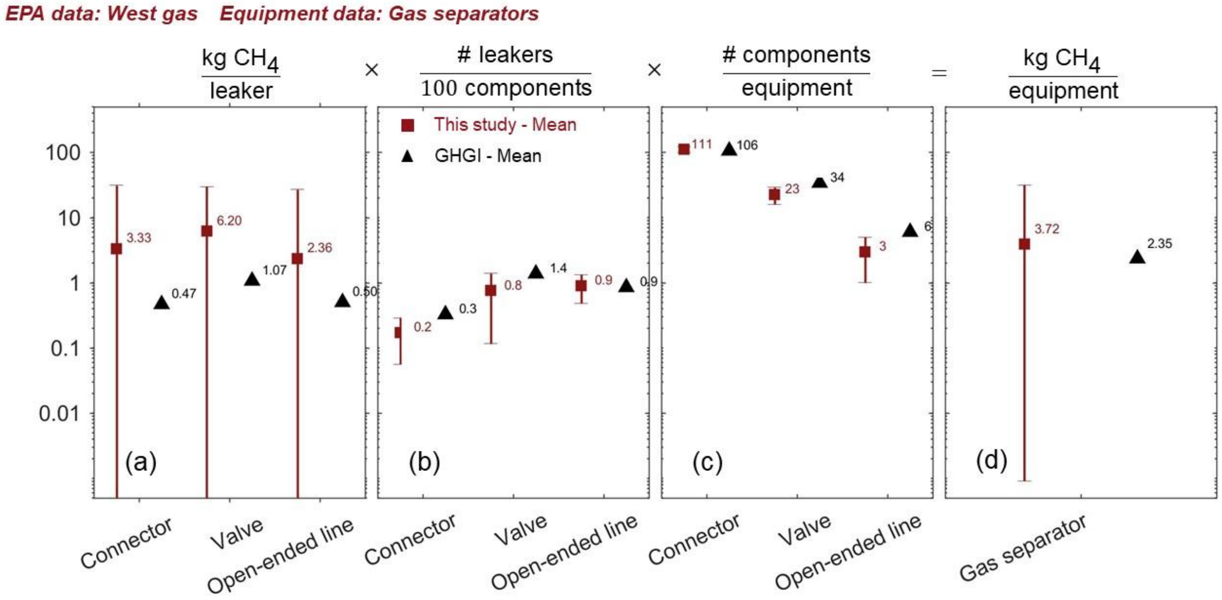

Supplementary Fig. 26: Decomposition of GHGI equipment-level emission factor for gas separators. Equipment-level emission factors are decomposed into constituent parts. Equipment-level emission factors for gas systems are a function of data for both Western systems (API 4589, [62]) and Eastern gas systems (Star Environmental, [16]). Here, we only show constituent data for Western gas systems. Error bars reflect the 95% confidence interval based on the 2.5 and 97.5 percentile values extracted from the empirical distributions and filled squares and triangles represent the mean.

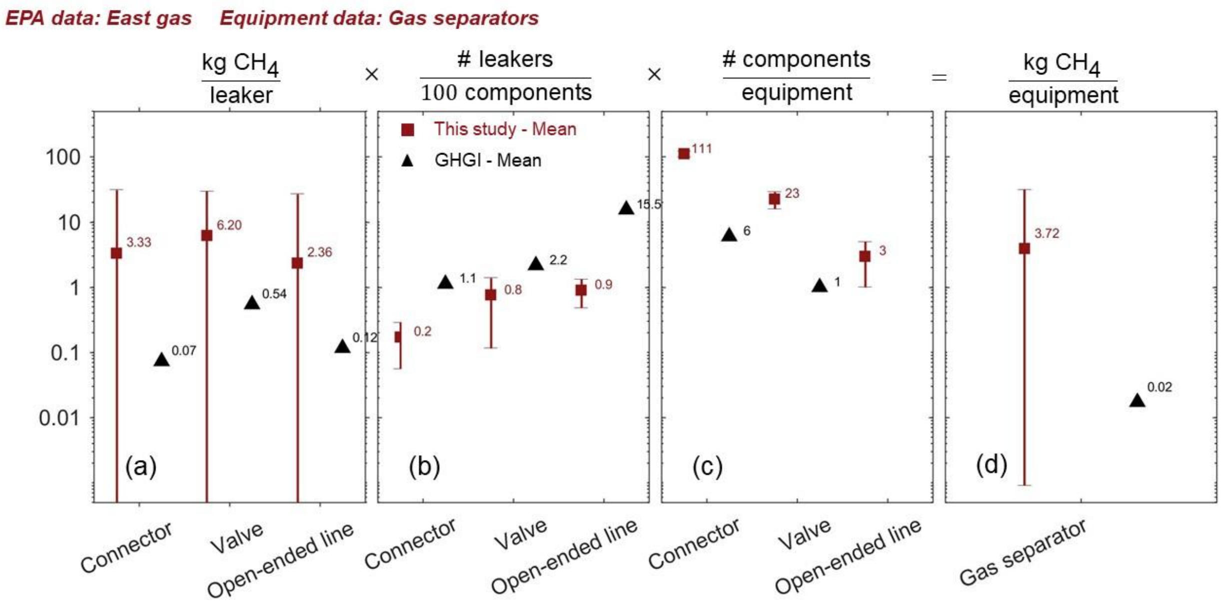

Supplementary Fig. 27: Decomposition of GHGI equipment-level emission factor for gas separators. Equipment-level emission factors are decomposed into constituent parts. Equipment-level emission factors for gas systems are a function of data for both Western systems (API 4589, [62]) and Eastern gas systems (Star Environmental, [16]). Here, we only show constituent data for Eastern gas systems. Error bars reflect the 95% confidence interval based on the 2.5 and 97.5 percentile values extracted from the empirical distributions and filled squares and triangles represent the mean.

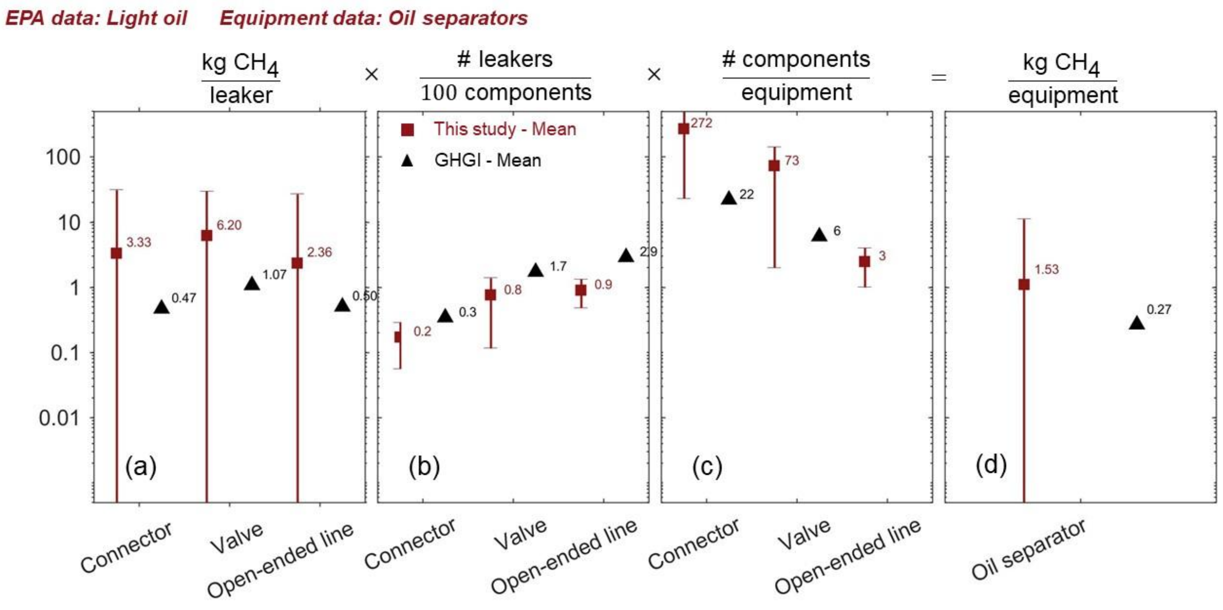

Supplementary Fig. 28: Decomposition of GHGI equipment-level emission factor for oil separators. Equipment-level emission factors are decomposed into constituent parts. Equipment-level emission factors for oil systems are a function of data for both light oil systems and heavy oil systems (both from API 4589, [62]). Here, we only show constituent data for light oil systems. Error bars reflect the 95% confidence interval based on the 2.5 and 97.5 percentile values extracted from the empirical distributions and filled squares and triangles represent the mean.

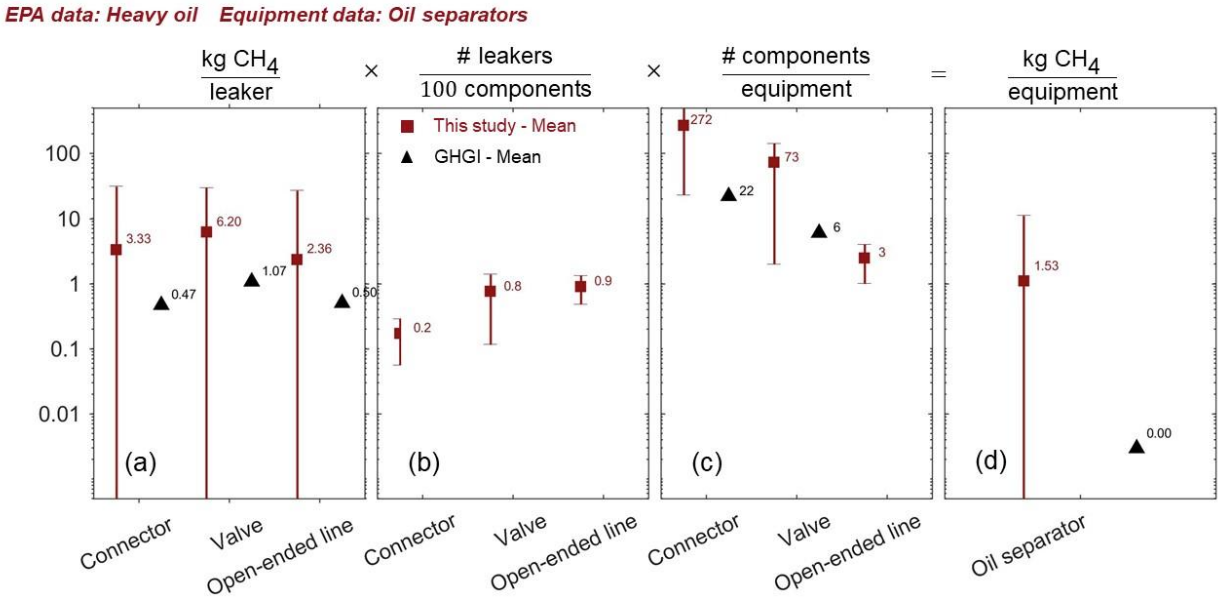

Supplementary Fig. 29: Decomposition of GHGI equipment-level emission factor for oil separators. Equipment-level emission factors are decomposed into constituent parts. Equipment-level emission factors for oil

systems are a function of data for both light oil systems and heavy oil systems (both from API 4589, [62]). Here, we only show constituent data for heavy oil systems. Error bars reflect the 95% confidence interval based on the 2.5 and 97.5 percentile values extracted from the empirical distributions and filled squares and triangles represent the mean.

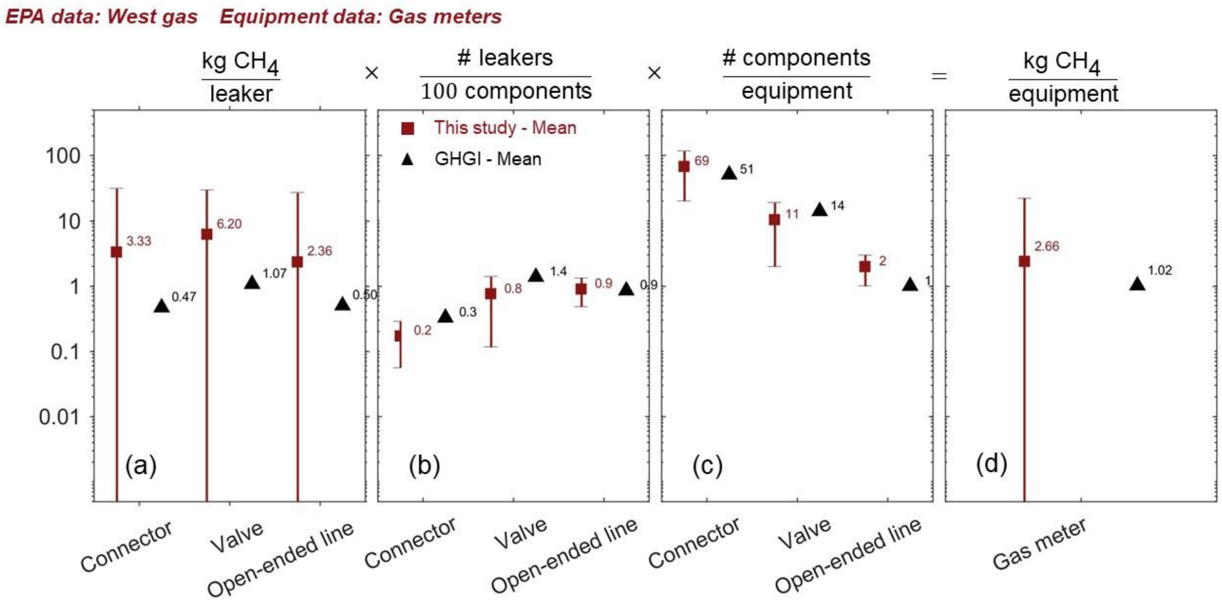

Supplementary Fig. 30: Decomposition of GHGI equipment-level emission factor for gas meters. Equipment-level emission factors are decomposed into constituent parts. Equipment-level emission factors for gas systems are a function of data for both Western systems (API 4589, [62]) and Eastern gas systems (Star Environmental, [16]). Here, we only show constituent data for Western gas systems. Error bars reflect the 95% confidence interval based on the 2.5 and 97.5 percentile values extracted from the empirical distributions and filled squares and triangles represent the mean.

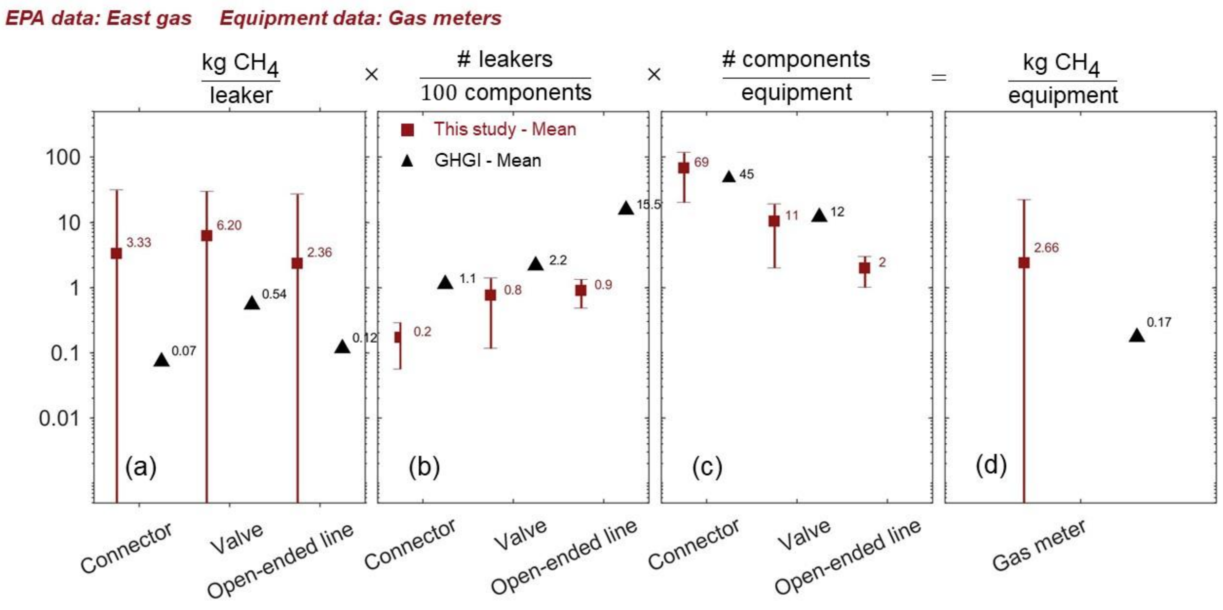

Supplementary Fig. 31: Decomposition of GHGI equipment-level emission factor for gas meters. Equipment-level emission factors are decomposed into constituent parts. Equipment-level emission factors for gas systems are a function of data for both Western systems (API 4589, [62]) and Eastern gas systems (Star Environmental, [16]). Here, we only show constituent data for Eastern gas systems. Error bars reflect the 95% confidence interval based on the 2.5 and 97.5 percentile values extracted from the empirical distributions and filled squares and triangles represent the mean.

6.3.Reconstructing GHGI emissions factor for storage tanks

Like our analysis of equipment leakage data, the goal of this section is to reconstruct the GHGI dataset for tank emissions and make a comparison with the data for this study. First, we describe the underlying data sources for the GHGI and how data are converted to emission factors. Second, we reconstruct the GHGI emission factors in two ways. We apply one method to verify that our reconstructed emission factors match those in the GHGI, and another separate method for a more consistent comparison with storage tank emission factors in this study. A complete description of our study's formulation of storage tank emissions can be found in Supplementary Methods 4 (and summarized in **Supplementary Fig. 8** and **9**).

6.3.1. Deriving emission factors – General approach

In the 2017 Greenhouse Gas Inventory (GHGI), the EPA revised its approach to calculating CH_4 emissions from crude and condensate storage tanks [30]. Prior to 2017, emission factors were "developed from default sample runs available through E&P Tank" [30,75]. Since 2017, the EPA has implemented an approach leveraging data from the Greenhouse Gas Reporting Program (GHGRP). However, it must be emphasized that emission factors through the GHGRP are still developed based on software programs such as E&P Tank (see further description below), only since 2017 the simulations are based on operator data (e.g., pressure, temperatures, throughput). Therefore, in contrast to our emission factors which are based on direct measurements of emissions, the GHGRP approach is based on simulated emissions volumes. One argument in favor of our approach, versus simulations, is the generally poor correlations that have been demonstrated between E&P Tank and HYSIS software and measurements [76].

The GHGRP is a program administered by EPA which collects greenhouse gas data from high emitting industrial facilities across multiple industry segments. Reporting requirements for petroleum and natural gas systems are described in the Code of Federal Regulations (CFR) Title 40 Part 98 Subpart W, which states that facilities emitting greater than 25,000 metric tonnes of CO_2 equivalent must report data to GHGRP [3]. GHGRP reporting requirements and emissions calculations differ for tanks storing throughput from wells producing at less than 10 bbl d^{-1} (referred to as small tanks) and tanks storing throughput from wells producing at greater than or equal to 10 bbl d^{-1} (referred to as large tanks).

In the current approach (since 2017), the GHGRP applies separate emissions calculation methods for large versus small tanks. Operators have a choice between calculation approaches 1 and 2 for large tanks. Operators apply calculation approach 3 for small tanks.

- Calculation approach 1: Use a software program such as API E&P Tank or AspenTech HYSYS [75,77].
- Calculation approach 2: Assume all CH_4 in liquid is emitted.
- Calculation approach 3: Emissions are calculated by multiplying a population emission factor by the number of wells and applying an average throughput of 2.2 bbl d^{-1}.

Within each of these categories (small/large, petroleum/natural gas), the GHGRP subcategorizes tanks as follows:

- Tanks with vapor recovery units (VRU)
- Tanks with flares
- Tanks venting to the atmosphere

For large tanks only, there is a separate inventory category for "malfunctioning separator dump valves".

The EPA uses data from the GHGRP to calculate storage tanks emission factors for the GHGI. The steps taken by the EPA in processing GHGRP data are reported in EPA [30]. These are also the steps we will use in the next section to reconstruct EPA emission factors. Note that the EPA elected to use a throughput-based approach, where emission factors are calculated per unit of crude throughput. This differs from the approach of this study (for unintentional emissions for thief hatches, PRVs) where emission factors are calculated per unit tank.

Briefly, the steps we take in reproducing GHGI emission factors on a per unit tank basis are as follows:

1) Gather GHGRP simulated data from [35]: Separate GHGRP data tables are available for large tanks and small tanks, respectively. Both tables list data at the sub-basin level.
2) Partition data sets into natural gas and petroleum systems: As in other areas, the EPA reports separate emissions volumes for natural gas systems and petroleum systems. Because natural gas production (and thus, gas-to-oil ratio) is not reported in the GHGRP, the EPA uses the subpart W formation type to partition data between petroleum and natural gas production systems. Data assigned to "high permeability gas", "shale gas", "coal seam", or "other tight reservoir rock" are allocated to natural gas production systems. All other formation types are allocated to petroleum production systems.
3) Partition data sets into tank categories: In addition to product stream, EPA also partitions data based on storage tank class (tanks with VRUs, tanks venting to the atmosphere, tanks with flares). Though emissions and tank counts are already allocated by storage tank class, throughput is only reported by sub-basin. Due to lack of data we assume that if multiple storage tank classes are reported in a single sub-basin, throughput is apportioned evenly proportional to tank counts between the storage tank classes.
4) Sum emissions and throughput: For natural gas systems and petroleum systems and all tank classes, the EPA sums reported emissions (kilotonnes) and liquids throughput (MMbbl).
5) Calculate emission factors, EF_i: For natural gas and petroleum systems and for each tank class i (i = VRU, flaring, or venting), the EPA calculates emission factors, EF_i, as

GHGRP reported emissions, $E_{i,GHGRP}$, divided by GHGRP liquids throughput for each tank class, $Q_{i,GHGRP}$.

6) Calculate total population emissions, $E_{i,pop}$: Because operators are only required to report to GHGRP if the facility emissions exceed a threshold of 25,000 tonnes CO_2e year^{-1}, GHGRP emissions and throughput totals are not comprehensive and therefore not suitable for direct use in the GHGI. The EPA therefore calculates total population emissions using the methods described in the next section.

6.3.2. Extrapolation methods

Because operators are only required to report to the GHGRP if facility emissions exceed a threshold of 25,000 tonnes CO_2e year^{-1}, GHGRP data (emissions, throughput, equipment counts) are not comprehensive. There are two approaches to extrapolating GHGRP activity to total population estimates (for the purposes of usage in inventories) which have been discussed in EPA GHGI documentation [30,78]: a throughput-based approach and a well-count-based approach. These extrapolations are required in any inventory source category which leverages GHGRP data (e.g., tank venting and flare methane).

The latest versions of the EPA inventory use a throughput-based approach for extrapolation [5,78]. Here, the emission factor for each tank class, EF_i, is calculated in units of emissions per unit liquid throughput as discussed above (kg CH_4 MMbbl^{-1}). Thus, to calculate total emissions, an activity factor of total throughput for a given tank class must be calculated. First, total population tank throughput ($Q_{tank,pop}$) is calculation from the following extrapolation.

$$Q_{tank,pop} = \left(\frac{Q_{tot,pop}}{Q_{tot,subW}}\right) \times Q_{tank,subW} \qquad (29)$$

Where the throughput-based extrapolation factor is calculated according to the ratio of total US liquids production ($Q_{tot,pop}$, EIA values as reported in [30]) to total liquids production reported to Subpart W ($Q_{tot,subW}$). $Q_{tank,subW}$ is the total liquids production sent to storage tanks and reported to Subpart W.

Next, the total can be apportioned by tank class (where *i*, for example, could be large tanks with flares) as follows.

$$Q_{i,pop} = \left(\frac{Q_{i,subW}}{Q_{tank,subW}}\right) \times Q_{tank,pop} \quad (30)$$

Where $Q_{i,pop}$ is total throughput for a particular tank class, and $(Q_{i,subW}/Q_{tank,subW})$ is the fraction of throughput sent to that particular tank class, as reported in Subpart W.

In EPA 2017, a well-based extrapolation approach was also considered. Here, emission factors are calculated in units of emissions per tank (kg CH_4 $tank^{-1}$). Thus, the activity factor must be total tank count. Total population tank count is extrapolated from Subpart W tank count using the following equation:

$$n_{tank,pop} = \left(\frac{n_{well,pop}}{n_{well,subW}}\right) \times n_{tank,subW} \quad (31)$$

Where the tank-based extrapolation factor is calculated according to the ratio of total US wells ($n_{well,pop}$, Enverus values as reported in [30]) to total wells reported to Subpart W ($n_{well,subW}$). $n_{tank,subW}$ is the total tank count reported to Subpart W.

Extrapolation figures are presented in **Supplementary Table 33** below.

Supplementary Table 33: Extrapolating reported Subpart W tank throughput and count to population totals.

	Condensate		Oil	
	Count	Throughput (MMbbl)	Count	Throughput (MMbbl)
Subpart W tank throughput/count	127469	228	169341	1306
Subpart W well throughput/count	306,553	297	216,527	2,140
Population well throughput/count	419,692	297	600,519	3,442
Extrapolation factor	1.4	1.0	2.8	1.6
Population tank throughput/count	174514	228.2	469653	2100.3

It can be seen that the well-based extrapolation factor is noticeably higher compared to the throughput-based extrapolation factor. What this means is that, as we would expect, more coverage of higher throughput wells is better compared to coverage of lower throughput wells in the GHGRP. But, should emissions from low throughput wells be discounted on the basis of the suggested relationship between emissions and oil throughput? Lyon et al. [49] conducted a helicopter based campaign in several basins and examined statistical relationships with basins and well-pad characteristics. While emissions were observed more frequently in oil-producing regions, Lyon et al. found basin and well-production characteristics to be poor predictors of emissions. This suggests that emissions are largely a stochastic process, and that emissions from low throughput sites should not be weighted less heavily compared to high throughput sites.

6.3.3. Reanalysis of average emission factors

To reconstruct emission factors, we begin by downloading GHGRP data from the Envirofacts GHG Customized Search tool [35] according to the following steps:

1. Access EPA custom GHG search site on July 25, 2020. URL: https://www.epa.gov/enviro/greenhouse-gas-customized-search
2. Select "Petroleum and natural gas systems"
3. Select reporting year 2015 and click "Go to step 2"
4. Click on the appropriate table for the subject, “EF_W_ATM_STG_TANKS_CALC1OR2” which pertains to emissions from large storage tanks.
5. Click on step 3: Select Columns button

6. Select all columns
7. Click on "Go to step 4"
8. Do not select any narrowing criteria
9. Click on "output to CSV file"

We conduct the same steps for small tanks, where the table downloaded from Envirofacts is titled “EF_W_ATM_STG_TANKS_CALC3”.

We attempt to reconstruct GHGI emission factors by following the steps described in the previous section. We reconstruct emission factors both on a throughput basis, to match those presented in the GHGI, and on a per-tank basis. We calculated tank basis emission factors for a closer comparison with emission factors from our study, which are on a tank-basis. Fortunately, in addition to tank throughput, the EPA also reported atmospheric storage tank counts to the GHGRP by tank class (in EPA [30], the tank-based approach is discussed as an alternative considered at the time). The steps in constructing a tank-basis estimate are like those outlined above. Steps 1-5 are identical, with the exception that tank counts are used for scaling of tank throughput. For extrapolation, we scale up activity data based on the ratio of subpart W reported well counts to actual well count in the United States. This extrapolation assumes that the number of tanks per well is consistent, on average, between reporting wells and non-reporting wells.

We present reconstructed emission factors in **Supplementary Table 34** (tank flashing emissions) and **Supplementary Table 35** (those due to malfunctioning dump valves). We also demonstrate how activity data is scaled up from subpart W (representative sample) to the national population. Note that, although we find reasonable agreement, our reconstructed values do not match perfectly with emission factors reported in the GHGI. It is possible that differences between our reconstructed emission factors and reported values in the GHGI could be due to differences in how throughput was allocated across storage tank classes. **Supplementary Table 34** and **Supplementary Table 35** also compare our extrapolated emissions totals with those presented in the GHGI. In most cases our extrapolations overestimate the GHGI, except for condensate tanks with a throughput-basis extrapolation. This overestimation is higher on a tank-basis versus a throughput-basis. We encounter these differences because tank throughput does not necessarily correlate with tank count. Without more detailed reporting on throughput per class, we cannot reconstruct these total emissions estimates more accurately.

Supplementary Table 34: Comparing flash emissions activity, emission factors, and total emissions for liquids storage tanks in natural gas systems and petroleum systems. Here, we compare this study's GHGI reconstructed values and reported GHGI values (reporting year 2015). Reconstructed values are calculated using GHGRP data downloaded from the EPA Envirofacts website [35] processed using steps outlined in [30]. Totals do not match exactly with totals reporting in the EPA GHGI.

	Condensate					Oil				
	Large			Small		Large			Small	
	VRU	Vent	Flare	Vent	Flare	VRU	Vent	Flare	Vent	Flare
**Reconstruction - Throughput basis**										
Throughput (MMbbl/year)										
Extrapolated Total			228.2					2100.3		
Fraction by tank type	11%	12%	55%	14%	7%	23%	14%	58%	5%	1%
Total by tank type	25.5	28.2	126.0	32.1	16.5	479.5	291.5	1215.3	96.5	17.5
Emissions factor (kg CH4/bbl/year)	0.003	0.202	0.005	0.364	0.007	0.008	0.207	0.007	0.161	0.003
Emissions (kilotonnes/year)	0.085	5.693	0.632	11.690	0.111	4.047	60.352	8.967	15.509	0.055
Total emissions (kilotonnes/year)			18.2					88.9		
**Reconstruction - Tank basis**										
Tank count										
Extrapolated Total			174514					469653		
Fraction by tank type	2%	7%	12%	66%	12%	7%	22%	43%	21%	7%
Total by tank type	3776	11645	21693	115874	21526	33899	104014	203521	97671	30546
Emissions factor (kg CH4/tank/year)	31.0	669.2	39.9	138.1	7.1	205.9	1000.5	76.0	274.0	3.1
Emissions (kilotonnes/year)	0.117	7.793	0.866	16.004	0.153	6.979	104.067	15.462	26.765	0.094
Total emissions (kilotonnes/year)			24.9					153.4		
**Greenhouse Gas Inventory - Throughput basis**										
Throughput (MMbbl/year)	25.2	32.5	124.7	35.2	17.5	394.0	325.7	970.8	85.3	28.8
Emissions factor (kg CH4/bbl/year)	0.003	0.173	0.005	0.518	0.006	0.008	0.149	0.007	0.045	0.002
Total emissions (kilotonnes/year)	0.085	5.633	0.626	18.211	0.110	3.257	48.573	7.217	3.823	0.044
Total emissions (kilotonnes/year)			24.7					62.9		

Supplementary Table 35: Comparing malfunctioning separator dump valve throughput-basis activity, emission factors, and total emissions for liquid storage tanks in natural gas systems and petroleum systems. Here, we compare this study's GHGI reconstructed values and actual GHGI values (reporting year 2015). Calculated using GHGRP data downloaded from the EPA Envirofacts website [35] processed using steps outlined in [30]. Totals do not match exactly with totals reporting in the EPA GHGI.

	Condensate	Oil
	Reconstruction - throughput-basis	
Throughput (MMbbl/year)	180	1986
Emissions factor (kg CH4/bbl/year)	0.0003	0.0030
Emissions (kilotonnes/year)	0.056	5.984
	Reconstruction - tank-basis	
Tank count	37114	341435
Failure rate	0.06%	1.56%
Emissions factor (kg CH4/tank/year)	724	1233
Emissions (kilotonnes)	0.017	6.586
	Greenhouse Gas Inventory	
Throughput (MMbbl/year)	182	1690
Emissions factor (kg CH4/bbl/year)	0.0003	0.0028
Emissions (kilotonnes/year)	0.055	4.816

6.3.4. Reanalysis of emission factor distributions and comparison with this study

Using our datasets constructed in the previous section, in addition to reconstructing average emission factors we can also construct emission factor distributions. To make an apples-to-apples comparison between this study's emission factor distributions and the GHGI emission factor distributions we will use emission factors with a tank-basis. For every sub-basin (or row in the GHGRP datasets), sub-basin emission factors are calculated by dividing sub-basin emissions by tank count.

This study's approach to calculating emission factors is described in Supplementary Methods 4. Briefly, our approach relies on quantified emissions measurements made at multiple sources on tanks, including pressure-relief valves and thief hatches to estimate unintentional emissions. These measurements are combined with flash emissions measurements from the HARC study, which we use to estimate intentional emissions from uncontrolled storage tanks.

In the GHGI emissions from storage tanks are classified as those emitted from controlled tanks (equipped with VRUs or flares) or those emitted from uncontrolled tanks (not equipped with a control device). Emissions due to malfunctioning separator dump valves are only possible at uncontrolled tanks. In contrast, our approach to estimating tank emissions is source-based (see Supplementary Methods 4). Therefore, to make a comparison between emissions distributions we adopt the GHGI classification scheme and bin emissions sources according to **Supplementary Table 36**.

Supplementary Table 36: Categorization of emissions sources in this study's model into GHGI categories. This categorization is required because emissions in our model database are directly attributed to specific tank components. Assumptions about how these emissions sources are reflected in controlled and uncontrolled tanks must be made post-hoc. In contrast, assumptions about specific emissions sources are inherent to the simulations made by emissions reporters.

	Dataset	
	This study	GHGI
Controlled	- Quantified measurements of emissions from hatches, holes, pressure-relief valves	- Simulated emissions from controlled tanks (based on operator reported efficiencies of VRUs and flares)<br>- Flaring emissions
Uncontrolled	- Quantified measurements of emissions from hatches, holes, pressure-relief valves<br>- Quantified measurements of flash emissions	- Simulated emissions from uncontrolled tanks<br>- Reported malfunctioning separator dump valve emissions

We make several observations based on the graphical comparison of this study's emission factor distributions with the reconstructed distributions of the GHGI (**Supplementary Fig. 32**). Perhaps most notably, our study finds much greater emissions from controlled tanks (i.e., tanks with VRUs or flares attached). This study's emissions from controlled tanks are based on empirical datasets [12,22,79] which identify multiple points of failure on tanks beyond the vent stack, VRU, or flare. Key points of failure driving emissions include open thief hatches and rusted holes. If thief hatches are open or the tank exterior is compromised with a hole, VRUs and flares are irrelevant as the gas will escape from the open port naturally as it is the path of least resistance. It should be noted that the GHGI also includes a separate category for malfunctioning separator dump valves. As they are reported in the GHGI, emission factors from malfunctioning separator dump valves can be high (100s of kgCH_4 day^{-1}), but because these are extremely infrequent, total emissions contributions are low.

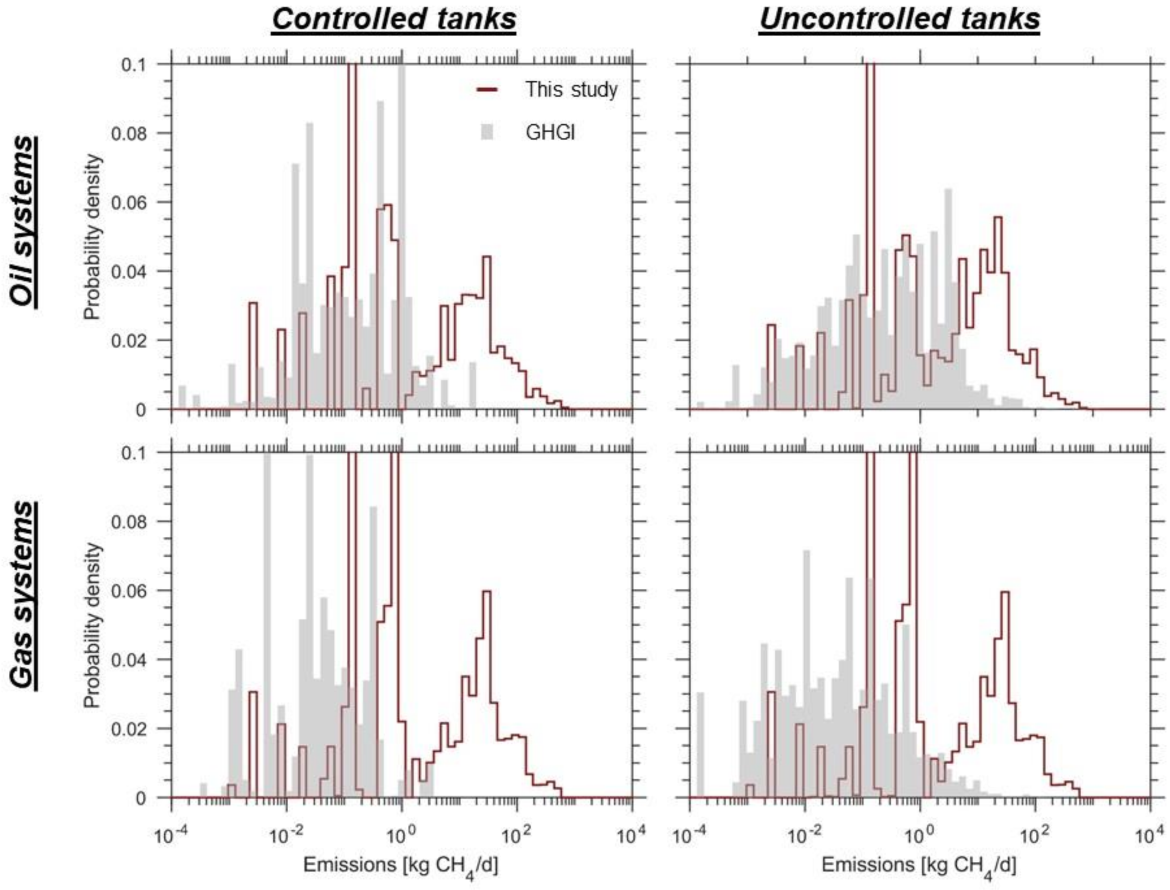

Supplementary Fig. 32: Comparison of storage-tank related emissions probability distributions [kg CH_4 tank^{-1} day^{-1}] between this study and the GHGI. Comparisons are made according to product-stream (natural gas versus petroleum, bottom-row versus top-row, respectively) and tank class (controlled versus uncontrolled, left-column versus right-column, respectively). For organization of emissions streams by tank class, see **Supplementary Table 36**. Note that that there are some simulated volumes over an order of magnitude lower compared to the lowest measured volume in our dataset. This is because measured emission volumes are limited by the lower detection limit of the measurement device, whereas simulated volumes are not. We speculate that spikes in our data are probably based on measurement lower-limits for Hi Flow samplers.

One outcome of the comparison in **Supplementary Fig. 32** between this study and the GHGI is that while there are clear differences in emissions distributions between controlled tanks and uncontrolled tanks and between gas sites and petroleum sites for the EPA, this is less the case for our model (i.e., the distributions for all categories look similar). This has to do with the fact that in our model emissions are dominated by unintentional emissions events from thief hatches,

pressure-relief valves, and rust related holes. Because each of these sources precede capture or control devices like VRUs and flares, these emissions can occur at either controlled or uncontrolled tanks. We elaborate on this result in the main text, with additional supporting evidence from aerial and ground surveys in **Supplementary Table 37**.

Supplementary Table 37: Ground and aerial surveys conducted with a focus on liquid storage tanks. These studies did not contain quantified emissions measurements useful for our model dataset (while Clearstone [22] did, the measurements were taken in Canada and thus may not be representative of the U.S.) but provide evidence that unintentional emissions events at storage tanks are frequent and large in magnitude, both at controlled and uncontrolled tanks.

Clearstone 2018 [22]	Clearstone conducted a ground-based, component-level survey of 333 O&NG well-pads in Alberta, Canada. Clearstone found that emissions from production storage tanks accounted for 28% of the total survey emissions. Thief hatch emissions were documented at 6 of 52 (11%) of surveyed tanks.
Lyon et al. 2016 [49]	Lyon et al conduct helicopter-based IR camera surveys of 8220 O&NG well pads in seven basins. Lyon et al detected a total of 494 unique high emissions sources at 327 wells pads (4% of wellpads, ranging from 1% in the Powder River basin to 14% in the Bakken), with tank hatches and tank vents comprising 92% of observations.<br>Correlations were also assessed between emissions detection frequency and several defining well-pad characteristics. As Lyon et al. conclude: “this study found statistically significant correlations between the presence of detected emissions and several well pad and operator parameters, these relationships were weak and GLM models were able to explain less than 15% of the variance. This low degree of predictability indicates that these large emission sources are primarily stochastic”.
Lyman et al. 2019 [80]	Lyman et al conducted a study with methods similar to Mansfied et al and Lyon et al. In this study, 3,225 well-pads are surveyed from the air, and 419 of the same well-pads are surveyed from the ground. Counterintuitively, well-pads with controlled tanks were more likely to have detected emissions compared to well-pads with uncontrolled tanks. According to Lyman et al., this was due to the fact that “most emissions were not from the control devices themselves, but from tank hatches, vents or piping upstream of control devices”.
Mansfield et al. 2017 [81]	Mansfield et al conducted a ground-based survey of 454 O&NG well-pads with controlled tanks only. Even though the tanks were controlled, 196 plumes were observed at 178 wells (39% of well-pads). 79% of plumes were from thief hatches or pressure-relief valves. As Mansfield et al. suggest: “the problem is not so much a failure of control devices themselves, but a failure to adequately deliver escaping gases to the control device”.
Englander et al. 2018 [82]	One year after the Lyon et al. survey, Englander et al. performed an infrared optical gas imaging survey in the Bakken formation using the same helicopter crew to examine persistence of large emitters. Englander et al. found that well pads emitting in 2014 were more likely (than random chance) to be emitting in 2015. Overall, emissions were detected at 11% of wellpads with tank vents or hatches accounting for 90% of observations.

6.3.5. Emission factor decompositions

In the main text we present and discuss a decomposition of emission factors for storage tanks. Here, emission factors are the summation of flash emission factors and unintentional emission factors (e.g., thief hatch emissions for this study and malfunctioning separator dump valves for the EPA). The decomposition for petroleum systems is shown as **Figure 5**. The decomposition for natural gas systems is shown as **Supplementary Fig. 33**. Note that although both our study and the GHGI are based upon GHGRP data for fraction of tanks without controls (second panel in decompositions), OPGEE applies a universal value of 51%, whereas the GHGI applies differentiated values for oil versus gas systems.

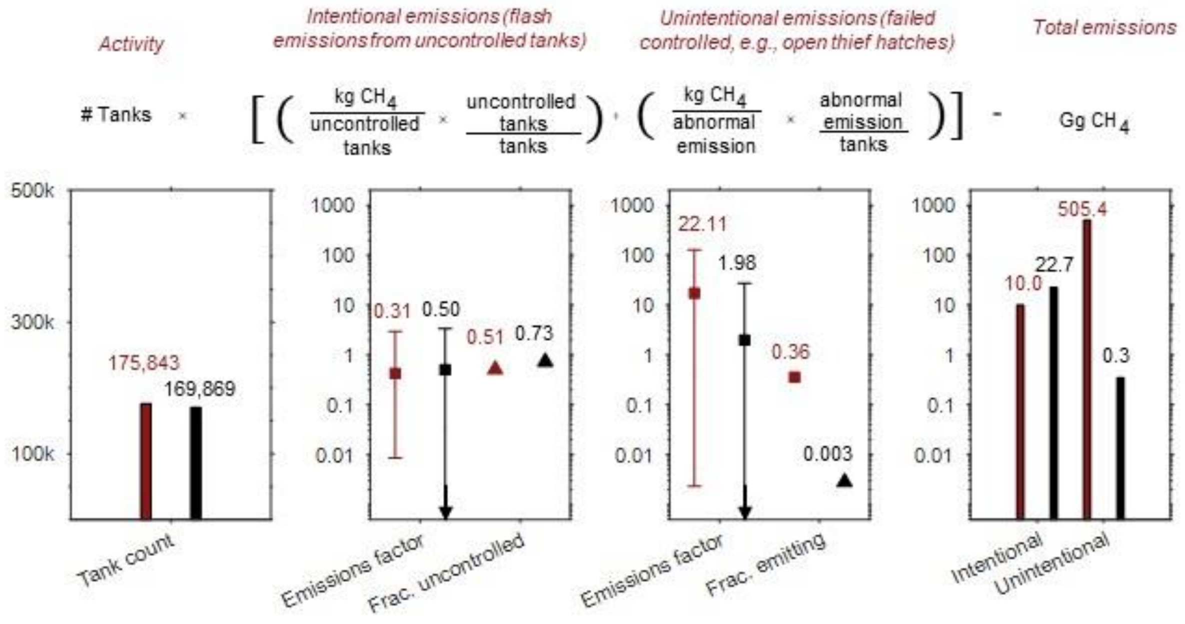

Supplementary Fig. 33: Example decomposition of total CH_4 emissions for condensate storage tanks. Total CH_4 emissions for condensate storage tanks in natural gas systems (for a decomposition of CH_4 emissions from condensate storage tanks in natural gas systems see Figure 5, main text) are decomposed into several constituent parts and compared with corresponding factors in the Greenhouse Gas Inventory. Error bars reflect the 95% confidence interval based on the 2.5 and 97.5 percentile values extracted from the empirical distributions and filled squares and triangles represent the mean. From left to right: Total activity, flash emission factor panel, unintentional

emission factor panel, and total emissions. Flash and unintentional emission factors are decomposed into emission factors (kg CH_4 per emitting tank) and control rates (fraction of total tanks emitting).

6.3.6. How does the GHGRP account for thief hatch emissions?

If the GHGRP were accounting for thief hatch emissions, it would be under 40 CFR 98.233(q) "Equipment leak surveys" or 98.233(j) "Onshore production and onshore petroleum and natural gas gathering and boosting storage tanks" [3]. However, components under the purview of "Equipment leak surveys" are listed in 98.232(c)(21) which explicitly states that it "does not include thief hatches or other openings on a storage vessel". 98.233(j) does not mention thief hatches or any other openings, although it is debatable if thief hatches could be contained within "storage tank vented emissions from produced hydrocarbons" (as the section is defined 98.232(c)(21)).

This point is clarified in the EPA's 2016 "Leak detection methodology revisions" (81 FR 86490, [83]) where the EPA "amend[ed] subpart W to add new monitoring methods for detecting leaks from oil and gas equipment in the petroleum and natural gas systems source category consistent with the NPSP subpart OOOOa". This required clarifying the definition of fugitive emissions components. The EPA notes that thief hatches are defined as a "fugitive emissions component" for the purposes of NSPS subpart OOOOa [2] but not for the purposes of subpart W. The decision is made to maintain the exclusion of thief hatches from the list of "fugitive emissions components" in subpart W as "the subpart W calculation methodology for storage tanks in 40 CFR 98.233(j) already includes emissions from thief hatches or other openings on storage vessels". The EPA defends this decision by noting how "if a reporter sees fugitive emissions from a thief hatch or other opening on a controlled storage vessel during an equipment leak survey conducted using OGI, the reporter should consider that information as part of the 'best available data' used to calculate emissions from that storage tank".

Unfortunately, based on how information is reported to the GHGRP, it is impossible to determine how many reporters are taking thief hatches and storage vessel openings into account.

6.4. Reconstructing GHGI emission factors for methane from flare stacks

Like the previous two sections, the goal of this section is to reconstruct the GHGI approach for uncombusted methane from flare stacks. First, we describe the underlying data sources for the GHGI and how data are converted to emission factors. Second, we reconstruct the GHGI emission factors.

6.4.1. Deriving emission factors – General approach

According to the 2020 EPA GHGI [5], flaring occurs during non-continuous operations such as well completions and workovers, continuous operations such as routine venting of flash gas from storage tanks, and flaring of associated gas when there is no economic route for sales. Other miscellaneous activities include flaring of dehydrator vents and well testing.

Supplementary Table 38: Summary of methane emissions due to flaring in the Greenhouse Gas Inventory. Here we present 2015 emissions from 2020 GHGI [5].

	Petroleum emissions [kt CH4]	Natural gas emissions [kt CH4]	Percentage production segment	Percentage total
Completions and workovers (C&W)	11.6	10.9	23%	11.62%
Total Tanks	7.3	0.7	8%	7.29%
Misc	12.4	5.6	19%	12.49%
Associated	48.8		50%	49.02%
Gathering and boosting		10.7		
Total NG processing	19.5			19.59%
Total Production Segment	80.1	17.2		
Total Production (minus C&W)	68.5	6.3		
Total	99.6	27.9		

The EPA GHGI methane volumes for flaring are based upon analysis of data reported to the Greenhouse Gas Reporting Program. On the “Envirofacts GHG Customized Search” website, CO_2 and CH_4 emissions due to flaring are found on various sheets (e.g., based on reporting requirements in Subpart W, tables specified below). Separate approaches are applied as follows:

(i) *Flares from associated gas*: As specified in 98.233(x), based on the estimated gas to oil ratio and composition of each well flare emissions are estimated using 98.233(n) (Data is contained in the sheet "EF_W_ASSOCIATED_NG_UNITS").

(ii) *Flaring of vented gas during completion and workover events*: As specified in 98.233(g)(4)(ii) and 98.233(h)(2)(ii), flare emissions during completions and workovers are calculated using 98.233(n) (Data is contained in the sheets "EF_W_COMP_WORKOVERS_FRAC" and "EF_W_COMP_WORKOVERS_NO_FRAC").

(iii) *Flares from storage tanks*: As specified in 98.233(j)(5), if the storage tank is equipped with a flare, flash gas is estimated and flare emissions are calculated using 98.233(n) (Data is contained in the sheets "EF_W_ATM_STG_TANKS_CALC1OR2" and "EF_W_ATM_STG_TANKS_CALC3").

(iv) *Flare stack emissions*: Section 98.233(n) describes how to calculate CO_2, CH_4, and N_2O emissions from flare stacks. Emissions listed in the table "EF_W_FLARE_STACKS_UNITS" are those that did not fit into one of the previously listed categories (flares from associated gas, flaring of vented gas during completion and workover events, and flares from storage tanks). However, according to EPA instructions to GHG reporters, "EF_W_FLARE_STACKS_UNITS" contains all activity data for all categories (on the EPA Confluence website it is stated "To the extent that monitoring information includes amounts associated with reported emission under [previously listed categories], those emissions should be deducted from the total emissions reported according to the methods in 98.233(n). If all emissions are deducted, then report 0 for the emissions and report all of the other required data elements" [84]). This includes total volume of gas sent to flare, average GHG mole fraction, combustion efficiency, and fraction of gas sent to flare when it was unlit

The calculation procedure for all categories that reference 98.233 (n) is:

$$\text{Flare CH}_4 = V_{gas,flare} \times X_{CH_4} \times [(1-\eta) \times Z_{lit} + Z_{unlit}] \tag{32}$$

Here Flare CH_4 (scf) is calculated based upon the volume of gas sent to the flare ($V_{gas,flare}$, scf), the mole fraction of CH_4 (X_{CH_4}), flare combustion efficiency (η, fraction gas combusted), fraction of feed gas sent to a burning flare (Z_{lit}), and fraction of feed gas sent to an un-lit flare (Z_{unlit}). Summary statistics of activity data for “EF_W_FLARE_STACKS_UNITS” are given in **Supplementary Table 39** below. **Supplementary Table 39** demonstrates how many operators apply the default flare combustion efficiency of 98% and rarely report gas sent to unlit flares.

Supplementary Table 39: Summary statistics from EF_W_FLARE_STACKS_UNITS

Column	Statistic	Value	Unit
GAS_SENT_TO_FLARE	Average	10.6	Mscf/day
GAS_SENT_TO_FLARE	Sum	86.1	Bscf/year
FLARE_COMBUSTION_EFFICIENCY	Average	0.976	[-]
FRACTION_OF_GAS_SENT	Average	0.004	[-]

The steps the EPA uses in transforming GHGRP data into GHGI totals is given in a series of memos [30,78]. These are also the steps we will use in the next section to reconstruct EPA emission factors. Note, like storage tanks, that the EPA elected to use a throughput-based approach, where emission factors are calculated per unit of crude throughput.

Briefly, the steps we take in reproducing GHGI emission factors are as follows:

(i) *Gather GHGRP tables*.

(ii) *Calculating emissions factors:*

a. *Flares from associated gas*: Sum of flaring CH_4 emissions divided by volume of oil produced as reported in “EF_W_ASSOCIATED_NG_UNITS”.

b. *Flares from miscellaneous processes*: Miscellaneous flare CH_4 emissions in “EF_W_FLARE_STACKS_UNITS” are not distinguished according to oil versus gas systems. For each facility, the proportion of oil wells and gas wells are calculated from the “EF_W_EQUIP_LEAKS_ONSHORE” table. These proportions are assigned using the facility column in “EF_W_FLARE_STACKS_UNITS” and emissions are calculated accordingly.

c. *Calculate total population emissions*: Subpart W emissions are extrapolation to the total population of facilities using methods described in the earlier section titled “Extrapolation methods”.

6.4.2. Reanalysis of average emissions factors

We attempt to reconstruct GHGI CH_4 emission factors from flares by following the steps described in the previous section. Like we did for tanks, we attempt to reconstruct emission factors both on a throughput basis, to match those presented by the GHGI, and on a per-well basis. Well-basis emission factors were developed for a closer comparison with the emission factors from our study.

We present reconstructed emission factors in **Supplementary Table 40**. Note that although we did find reasonable agreement our reconstructed values do not match perfectly with emission factors reported in the GHGI.

Supplementary Table 40: Comparing flare methane emission activity, emission factors, and total emissions in natural gas systems and petroleum systems for GHGI reconstructed and reported values (reporting year 2015).

	Natural gas systems				Petroleum systems			
	Assoc.	Misc.	Tanks-small	Tanks-large	Assoc.	Misc.	Tanks-small	Tanks-large
**Reconstruction - Throughput basis**								
Throughput (MMbbl $year^{-1}$)[1]	-	31,473	16	126	555	3,434	17	1,215
Emissions, subpart W (kilotonnes $year^{-1}$)	-	2.2	0.1	0.6	44.3	8.5	0.0	5.6
Emissions factor (kg CH_4 $bbl^{-1}year^{-1}$)[2]	-	0.0001	0.007	0.005	0.117	0.004	0.003	0.007
Total emissions (kilotonnes $year^{-1}$)	-	2.9	0.1	0.6	65.0	12.5	0.1	9.0
**Reconstruction - Wells basis**								
# Flaring equipment[3]	-	433430	22,145	22,317	66395	571761	28,698	191,209
Emissions, subpart W (kilotonnes $year^{-1}$)	-	2.232	0.111	0.632	44.3	8.525	0.034	5.575
Emissions factor (kg CH_4 $equip^{-1}year^{-1}$)[4]	-	7.3	7.1	39.9	1761.7	39.4	3.1	76.0
Total emissions (kilotonnes $year^{-1}$)	-	3.2	0.2	0.9	117.0	22.5	0.1	14.5
**Greenhouse Gas Inventory - Throughput basis**								
Throughput (MMbbl/year)[1]	-	31,807	17.5	124.7	381.5	2877.8	28.8	970.8
Emissions factor (kg CH_4 $bbl^{-1}year^{-1}$)[2]	-	0.0002	0.006	0.005	0.128	0.004	0.002	0.007
Total emissions (kilotonnes $year^{-1}$)	-	5.6	0.1	0.6	48.8	12.4	0.0	7.2

[1]For all items throughput activity is MMbbl $year^{-1}$, except for miscellaneous condensate the units of throughput activity are Bscf $year^{-1}$

[2]For all items units are kg CH_4 $bbl^{-1}year^{-1}$, except for Miscellaneous condiate the EF units are kg CH_4 mscf $year^{-1}$

[3]For all tank items, the activity basis is # tanks, for all other items the activity basis is # wells

[4]For all tank items, the emission factor unit is kg CH_4 $tank^{-1}year^{-1}$, for all other items the emission factor unit is kg CH_4 $well^{-1}year^{-1}$

6.4.3. Reanalysis of emission factor distributions and comparison with this study

Using our datasets constructed in the previous section, in addition to reconstructing average emission factors we can also construct emission factor distributions for the GHGI. To make an apples-to-apples comparison between this study's emission factor distributions and those of the GHGI we will use emission factors on a flare-stack basis (this study's approach to calculating emission factors is given in Supplementary Methods 5). Our approach is very similar and uses activity data from the GHGRP but uses empirical measurements of flare combustion efficiency and rate of unlit flares. Comparing emission factor distributions of our approach with those reported to the GHGRP we notice that total emissions are highly sensitive to the rate of unlit flares. This isn't surprising given that the volume of gas that is sent to flares and intended for combustion.

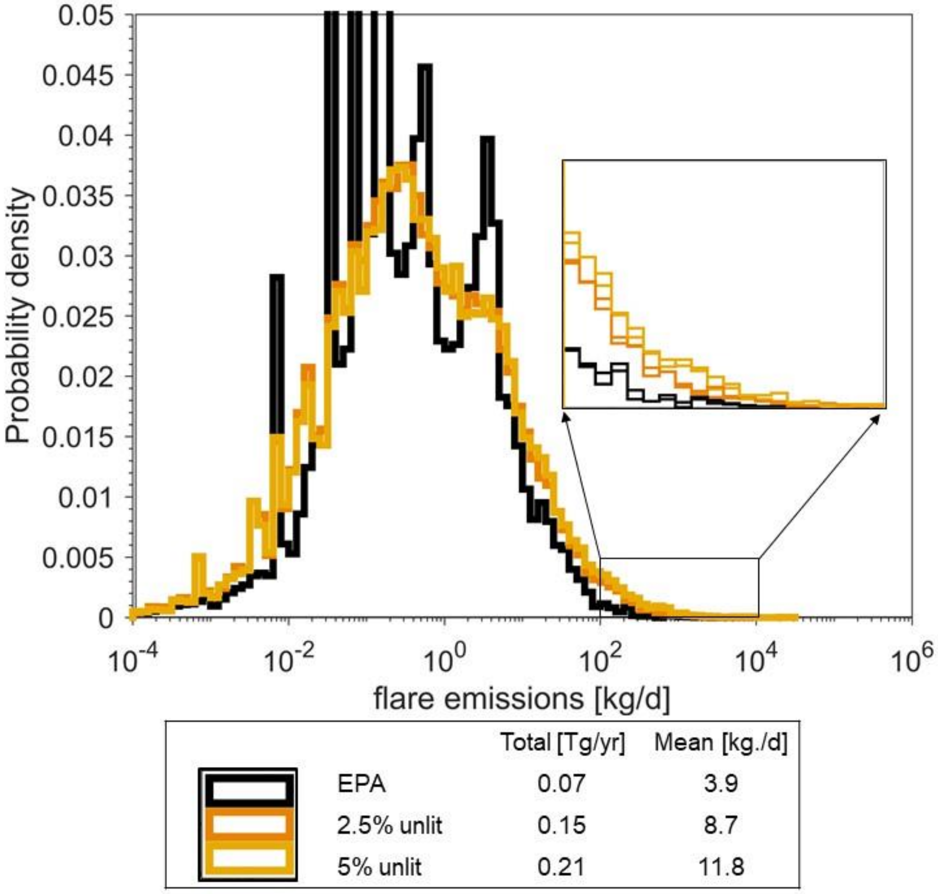

Supplementary Fig. 34: Comparison of simulated CH_4 emissions per flare stack for various parameterizations of η and Z_{unlit}. For the "EPA" parameterization, we apply η = 98% and Z_{unlit} = 0.4%. For others, we apply an η distribution according to Gvakharia et al. [47], and variable Z_{unlit}.

7. Supplementary Methods 7: Summary of surveyed studies

Key details of each study can be found in **Supplementary Table 41**. Additional details are provided in the following sections.

Supplementary Table 41: Summaries of studies used for emissions measurements, fraction components emitting, or component counts

**Allen et al 2013 [24]**	
Location	Gulf Coast, Midcontinent, Rocky Mountain, and Appalachian production regions. All wells sampled were hydraulically fractured
Types of equipment	Production phase and drilling/completions operations
Emissions quantification data:	Various components sampled. According to Allen et al., “The focus in this work was on measuring emissions from pneumatic pumps and controllers and measuring leaks from equipment, pipes, flanges and fittings”
Component counts	Counts of components surveyed was not reported
Fraction emitting	Fraction of components leaking was not reported (given that total components weren’t counted).
**Allen et al 2014a [26]**	
Location	Sampling was conducted across four regions (Appalachian, Gulf Coast, Mid-continent, Rocky Mountain) and a stated goal was to "sample a cross section of typical facilities" across different service types.
Types of equipment	Pneumatic controllers
Emissions quantification data	Allen et al quantified emissions from 377 pneumatic controllers (PC). Each PC is classified as using its actuation pattern as “intermittent” or “continuous”, as well as by its emissions rate as “low-bleed”, “high-bleed”, “intermittent”, or “not classified”
Component counts	Allen 2014a gives the number of pneumatic devices measured per site visited, total number of pneumatic devices per site, count of wells per site, and pneumatic devices per well. See Allen 2014a, SI table S6-1.
Fraction emitting	According to the manuscript, 75% of wells have pneumatic controllers with documented emissions (the other 25% have non-pneumatic controllers/actuators)
**Bell et al 2017 [25]**	
Location	Fayetteville AR
Types of equipment	All upstream equipment
Emissions quantification data	Bell et al. 2017 conducted on-site measurements using a High-flow sampler at 261 facilities
Component counts	Counts of components surveyed was not reported
Fraction emitting	Fraction of components leaking was not reported (given that total components weren’t counted).
**ERG 2011 [12]**	
Location	Fort Worth, TX (Located in the Barnett shale)
Types of equipment	Production sites, compressor stations, etc.
Emissions quantification data	ERG 2011 supplemental information contains ~2000 quantified leaks with leak type, site type, and other information. Leaks are broken down into 95 component categories, many more than other studies. ERG does not give equipment categories. Each measurement contains supplemental text or notes, but these are not standardized and therefore difficult to parse for information.

Component counts	Supplemental information provides a mix of equipment-level and component-level counts. The dataset does not give information about which valves and connectors are associated with each piece of equipment. This makes the dataset not useful for estimating, for example, the number of connectors or valves per compressor
Fraction emitting	Because component counts are available for connectors, valves, and tanks, we can estimate fraction of components emitting. However, ERG note that only one in ten components were screened using Method 21. Therefore, in calculating fraction of components emitting, the emitter count < 10,000 ppmv was multiplied by ten.

Thoma et al 2017 [51]

Location	Uintah Basin, UT
Types of equipment	Pneumatic controllers
Emissions quantification data	Thoma et al. surveyed 80 pneumatic controllers (PC). All PCs in the Thoma study are assigned a minimum continuous emissions rate of 0.1 scf/h
Component counts	n/a
Fraction emitting	n/a

Pasci et al 2019 [23]

Location	Permian, Anadarko, Gulf Coast, and San Juan basins
Types of Equipment	Production and gathering and boosting segments
Emissions quantification data	Supplementary information contains 331 speciated leaks, of which 261 are at well sites
Component counts	At each site an equipment count and component count was determined. Components were classified into one of 12 categories
Fraction emitting	Both emitter count and component counts are reported so total fraction of components emitting can be determined by component

API 4589 [15]

Location	Various US
Types of equipment	Sites 1-4 were light crude oil fields. Sites 5-8 were heavy crude oil fields. Sites 9-12 were gas production fields. Sites 13-16 were gas plants. Sites 17-20 were offshore fields. A later study (API 1995 or API#4615, [62]) added four more gas plants as sites 21-24 .
Emissions quantification data	Appendix C contains concentration measurements (ppmv) for all screened components. These concentrations can be converted to emissions fluxes using correlation equations and "pegged source factors". Technology applied: Method 21 flame ionization detector
Component counts	Appendix B of API 1993 contains activity data on component counts for pieces of equipment contained across all 20 sites, with a total of 1446 pieces of equipment included. Component counts are given for the following component types: Connectors, valves, open-ended lines, compressor seals, pump seals, pressure-relief valves, and other. The miscellaneous "other" category includes dump-lever-arms, polished-rod pumps, and miscellaneous.
Fraction emitting	API 1993 contains data on the fraction of components classified as emitters via the ISV threshold of >10 ppmv. For the 20 sites, the fraction of components classified as emitters is given in in Table 1-1 of API 1993. In the appendices, the-per leak ISV readings are presented which would allow one to adjust for a different fraction of components emitting.

Clearstone 2018 [22]

Location	Alberta, Canada
Types of equipment	Upstream oil and natural gas sites and compressor stations

Emissions quantification data	Appendix Raw data provided by Lindsay Campbell of the Alberta Energy Regulator to Jeff Rutherford on November 4, 2019<br>Technology applied: Hi Flow Sampler for leak quantification
Component counts	Field work included a detailed equipment and component count inventory (12 standardized component types).
Fraction leaking	Both leakage count and component counts are reported so total fraction leaking can be determined by component

Allen 2013: "Measurements of methane emissions at natural gas production sites in the United States"

Allen et al. [24] present results from one of the earliest studies in the recent era of methane quantification research and the first results from the large multi-study EDF campaign. Allen et al. quantified emissions from 190 onshore gas sites, including 150 production sites, 27 well completions flowbacks, 9 liquids unloading, and 4 well workovers. Gas sites were located in the Gulf Coast, Mid-continent, Rocky Mountain, and Appalachian regions of the United States. It is noted in the study that measurements were made exclusively at shale gas, hydraulically fractured well sites. Quantified volumes are presented for a variety of emitters.

The approach taken by this study was to first scan a site using an infrared camera to identify possible emitters. All identified emitters were measured with a high-flow sampler. Component counts per equipment were not inventoried. Rather, emitters are reported "per well" and emissions are scaled according to well count.

The Allen et al. dataset contains 124 measurements of tank vents. However, according to correspondence with David Allen (August 17, 2018) these measurements were for exploratory purposes only and should not be used in analysis.

Allen 2014: "Methane emissions from process equipment at natural gas production sites in the United States: Pneumatic controllers"

Allen et al. [26] examined pneumatic controllers at well sites, focusing on natural gas production sites. Sampling was conducted across the Gulf coast, Mid-continent, Rocky Mountain, and Appalachian regions of the United States. A stated goal of the study was to "sample a cross-section of typical facilities" across different service types. Measurements were made from 377 pneumatic controllers.

The majority of measurements (333 of 377) were made using a Fox flow meter. The remainder of measurements were made using a high-flow sampler. Because pneumatic controllers activate discontinuously, average emissions were taken over 15 minute periods for both the flow meter

and High flow sampler approaches. Allen et al. note that it's possible that some emissions may have been missed if devices did not activate during the 15 minute period.

Supplemental information files from the study include number of pneumatic controllers per well, as shown in Table S6-1 in file "es5040156_si_002.xlsx". Table S4-1 gives many properties of each measured pneumatic controller, including the basic application and detailed application. Unfortunately, though some pneumatic controllers were found on dehydration systems or separators, the study does not report equipment counts for those types of equipment per site, only well counts per site.

Allen et al. 2015: "Methane Emissions from Process Equipment at Natural Gas Production Sites in the United States: Liquid Unloadings"

Allen et al. [34] generate emission factors through direct measurements. Methane emissions from liquid unloadings were measured at 107 natural gas wells, both with and without plunger lifts. Emission measurements were taken by directing production separator gas through a temporary stack. Volumetric flow rates were calculated by multiplying the measured gas velocity by the cross-sectional area of the stack.

Allen et al stratify activity data at two levels. First, based on whether the well is with or without plunger lift, and second, based on the frequency of unloading events. The source of this activity data was a survey of participating companies in the study. According to Allen et al. [34], "while this work uses total well counts from the 2012 GHGRP FLIGHT data in estimating national emissions, event counts will be based on data from a survey of companies participating in this work. One reason for using survey data for event counts is the lack of complete data on event frequency for non-plunger wells in the 2012 GHGRP FLIGHT data… A second reason for using the survey data collected in this work, rather than GHGRP FLIGHT data for event counts, was apparent quality assurance issues in event counts in the GHGRP FLIGHT data".

Bell et al. 2017: "Comparison of methane emission estimates from multiple measurement techniques at natural gas production pads"

Bell et al. [25] present results from field studies in the Fayetteville shale gas play. No additional details are provided on the service type (e.g., gas versus oil) or completions type (e.g.,

hydraulically fractured) of these wells. Several measurement techniques were compared, including onsite measurements, the dual tracer flux ratio method, and the EPA Other Test Method 33a. 268 gas production facilities were sampled, and on-site measurements were made at 261 facilities.

Onsite direct measurement involved detection using “a combination of optical gas imaging and handheld laser methane detection” and quantification using a high-flow sampler. Only onsite direct measurements were used for this study.

Several “zero” values are contained in the Bell et al. dataset. This includes data points classified as “Below High Flow Range” (some but not all equal to zero) and “Observed not measured”. Most measurements labelled “Below High Flow Range” and all “Observed not measured” were filtered out of our final dataset.

Eastern Research Group (2011): “City of Fort Worth Natural Gas Air Quality Study”

This project was conducted by Eastern Research Group (ERG) for the Fort Worth Natural Gas Air Quality Study (FWAQS) in 2010 and 2011. Fort Worth is located within the Barnett shale, the formation where hydraulic fracturing was first implemented.

The ERG study had unique advantages compared to other component-level campaigns. Given that the municipal government granted full site access to ERG teams (the City Gas Inspector, who always has access to well pads, was present for all site visits), results of the ERG study are likely less subject to sampling bias. Most studies rely upon voluntary study partners, potentially oversampling better-performing operators.

These unique access provisions provided additional benefits for emissions quantification. First, it is less likely that tank emissions were missed given that each IR camera survey involved “climbing up the stairs to the tank walkway in order to view each thief hatch and pressure relief valve (PRV) vent line” [12]. Other studies have noted difficulties measuring tanks due to safety issues [85]. If emissions were detected on tanks, remarkable efforts were made in quantification. For example, ERG document the use of extensions to the High Flow Sampler tubing to access out-of-reach components, large nylon bags to sample oversized openings such as thief hatches, and even the “use of a man lift and operator to provide the point source crew with access to the

emission point" [12,86]. Finally, beyond the unique access provisions of this study, emission sources were classified with a high degree of detail.

The FWAQS team deployed two teams to detect leaks:

- A team with an infrared camera (FLIR) surveyed all components at the site and all observed leaks were reported.
- A second team used Method 21 testing (using a toxic vapor analyzer) to survey 1 out of every 10 connectors and valves.
- While the IR camera survey was taking place, the second crew member would conduct an equipment/component count which included number of storage tanks and counts of connectors and valves. The report notes that only valves were counted, and a multiplier of 7 was used to estimate connector counts.

A Bacharach Hi Flow sampler was used to measure emission points identified with the FLIR camera or Method 21 screening > 500 ppmv. ERG [12] document extensive detail on 388 sites visited. These data are recorded on sheet "Executive PS Site Summary" in worksheet "EmissionsCalculations.xlsx". The sites are classified by type of gas and listed with operator and location. Equipment counts for each site include number of wells, tanks, and compressors per site.

The ERG report documents significant emissions from tanks. For example, "the largest source of emissions detected with the IR camera was leaking tank thief hatches". It is confusing, however, that the study notes that "other sources of emissions, including but not limited to, storage tank breathing and standing losses … were not calculated. Non-routine emissions such as those generated during upsets or from maintenance, startup, and shutdown activities were also not measured or calculated as part of this study unless they were observed at the time of the site visit." We assume this statement is intended to reflect that no additional calculations were made to reflect emissions resulting from the normal functioning of uncontrolled tanks (e.g., emissions released during the filling of tanks or due to diurnal changes in temperature). Estimates of tank flashing and working and breathing emissions requires measurement of tank vents over periods up to 24 hours (to capture a full diurnal cycle). However, measurements of tank thief hatches, pressure-relief valves, and vents were made with the High Flow sampler if they were identified by a FLIR camera or Method 21 screening.

ERG collected canister samples at a subset of well pads and laboratory analysis of the canister samples is used to speciate the emitted gas (the High Flow Sampler only reports emissions as a flow rate of natural gas). Because canister samples for speciation were not obtained at all facilities, correlation equations were developed based on the emissions results from canister measurements (in total organic carbon, lb/year) and the corresponding gas flow rate of the High Flow Sampler (reported %CFM). The methods applied are based on the guidance from the EPA's Protocol document [17].

ERG also note that some low-level emitters were below the detection capability of the High flow sampler. For Phase II measurements, some of these low-level emissions were estimated using default zero factors from the EPA Protocol document [17].

Thoma 2017: "Uinta Basin Well Pad Pneumatic Controller Emissions Research Study"

Thoma et al. [51] analyzed a total 80 pneumatic controllers at five oil sites (containing 6 wells) and three gas sites (containing 12 wells) in the Uinta Basin. Emissions were quantified from the pneumatic controllers and counts of pneumatic controllers per well were computed.

Screening was conducted with both an infrared camera (FLIR) and Method 21 testing using a handheld probe. Emissions quantification was conducted using a combination of flow meter measurements and high flow sampler measurements.

Pacsi et al. 2019: "Equipment leak detection and quantification at 67 oil and gas sites in the Western United States"

Pacsi et al. [23] analyzed 67 oil and natural production and gathering and boosting sites in the Permian, Anadarko, Gulf Coast, and San Juan basins. Measurements were taken at both oil and natural gas producing sites (no GOR threshold is provided). Nearly 84,000 components were monitored.

Leak screening was conducted with a combination of Method 21 (flame ionization detectors) and optical gas imaging cameras. For each leak identified, emissions rates were measured with a high flow sampler.

American Petroleum Institute (1993): "Fugitive Hydrocarbon Emissions from Oil and Gas Production Operations"

The API report "Fugitive Hydrocarbon Emissions from Oil and Gas Production Operations", or API-4589, gives results from the extensive API/GRI studies of 1993 [15]. API 1993 was also used in EPA documents, including serving as the source material for the EPA 1995 emission factor update [17] and the EPA 1996 15-volume analysis of methane emissions from oil and gas industries [63]. API 1993 contains information from 20 sites sampled for fugitive emissions from components.

In API 1993, EPA Method 21 was followed. A total of 184,035 components were screened across 20 sites (**Supplementary Table 42**). During screening, instrument screening values (ISVs) greater than 10 ppmv were considered "emitters". A total of 4796 components (2.6% of total) were considered emitters with ISV > 10 ppmv. A total of 705 of these emitters were sampled to quantify leakage rates and speciate the gases being leaked.

For our purposes, site 1-12 are useful (sites 13-16 were gas plants, and sites 17-20 were offshore fields). Sites 1-4 were light crude fields, sites 5-8 were heavy crude fields, and sites 9-12 were gas production fields.

Appendix C of the Western dataset reports all screening concentrations measured (in ppmv) with the associated component for each measurement. Information on total components screened is contained in Appendix D. All data in these appendices were digitized from scanned PDF and manually double checked for accuracy. The Western dataset classifies equipment in the following categories: connectors, open-ended lines (OELs), valves, pressure-relief valves (PRVs), compressor seals, and other.

It should be noted that a continuation study was published in 1995 by API [9] (API 4615) which adds to the original 20 sites an additional 4 gas plants.

Supplementary Table 42: Components screened and found leaking in the API 4589 dataset [15]

Site type	Site #	Components screened	Components ISV > 10 ppmv	Components ISV > 10,000 ppmv	Samples collected	Fraction emitting @ 10,000 ppmv
Light crude	1	27155	522	247	43	0.91%
Light crude	2	14620	290	106	39	0.73%
Light crude	3	4095	84	27	58	0.66%
Light crude	4	2782	95	35	28	1.26%
Heavy crude	5	6362	19	0	9	0.00%
Heavy crude	6	2799	21	2	9	0.07%
Heavy crude	7	2696	8	0	6	0.00%
Heavy crude	8	1899	14	0	8	0.00%
Gas	9	14066	324	126	23	0.90%
Gas	10	9374	316	116	36	1.24%
Gas	11	9094	628	318	22	3.50%
Gas	12	7644	245	88	36	1.15%

Clearstone (2018): "Update of Equipment, Component, and Fugitive Emission Factors for Alberta Upstream Oil and Gas"

Clearstone [22] documents screening performed in Alberta, Canada in August and September 2017 at 333 locations in order to improve Alberta's methane emissions factors and associated confidence intervals. In addition to emissions quantification, component counts and fraction of components emitting are also documented. This study targeted fugitive emissions specifically and includes a comprehensive and useful glossary of component and equipment definitions.

Texas Environmental Research Consortium 2009 ("HARC" report):

The "HARC study" is a direct measurement campaign (May 2006) of vent gas emissions from 33 tanks, covering both crude and condensate service across a range of API gravities, in the Dallas-Forth-Worth, Houston-Galveston-Brazoria, and Beaumont-Port Arthur counties. Calculated emission factors are intended to reflect "tank working, breathing, and flashing losses" [32], and represent uncontrolled emissions (e.g., tanks without vapor recovery units or flares). Prior to measurement, tanks were inspected for the presence of holes (due to rust) or open thief hatches. If tanks had holes no measurements were made, and all thief hatches were closed.

Tank emissions were measured by sampling the flow rate of vent gas through pipes located at the top of the tanks using a Fox Instruments Thermal Mass Flow Meter. Flow rates were measured over approximately 24 hours.

Measurements range from 0.002 – 5.945 kg CH_4/bbl and average 0.94 kg CH_4/bbl (with three tanks removed, 17, 25, and 26, due to unphysical mole fractions or abnormally high emissions rates).

Gas Research Institute 1996

In 1996, the Gas Research Institute (GRI) released a 15-volume compendium estimating US natural gas industry GHG emissions (see also the published summary by Kirchgessner et al [87] and methodology documented in the EPA Protocol document [17]). For the purposes of fugitive and vent methane emissions, the most useful document is "Volume 8: Equipment Leaks". As of the 2020 GHGI, emission factors for most equipment leaks in the NG systems are based off GRI volume 8 [63] (henceforth referred to as the "GRI report").

Star Environmental 1995

The 1995 Star Environmental report "Fugitive Hydrocarbon Emissions: Eastern Gas Wells" provided screening data for the Eastern US [16]. Star Environmental generated three sets of emission factors using different permutations of two campaign datasets and applying either pegged source factors or the Hi Flow Sampler. It is not clear which set of emissions factors were applied for the GRI report, however we presume the approach chosen was the "Hi Flow approach" emission factors (which use campaign 2 data and the Hi Flow sampler).

For campaign 2, a total of 12,853 components were screened across 8 different gas sites (**Supplementary Table 43**) and a subset of the leaking components were quantified. All instrument readings > 100,000 ppmv (21 readings) and half of instrument readings > 10,000 ppmv (81 readings) were quantified with a Hi-Flow sampler and documented in the report Appendix. Although seven classes of components are noted as getting inventories at the beginning of the report (valves, flanges, open-ended lines, connectors, compressor seals, pump seals, and miscellaneous), emission factors are only developed for four classes (valves, connectors, open-ended lines, and miscellaneous.

Emission factors in Star Environmental were developed in a similar fashion to API 4589 [15] (Default-zero values were applied to screening values < 10 ppmv and EPA protocol correlation equations were applied to screening values >= 10 and < 9,999 ppmv). However, it is important to emphasize a key difference between the approaches of the Western gas and petroleum systems and the approach of Eastern gas systems. Recall that emission factors derived for Western gas systems and petroleum systems applied pegged source factors for screening measurements > 10,000 ppmv. For Eastern gas systems quantified Hi-Flow Sampler data was applied to screening data > 10,000 ppmv.

Supplementary Table 43: Components screened and found leaking in the Eastern dataset

Site number	Well sites	Components screened	Components ISV > 10 ppmv	Components ISV > 10,000 ppmv	Fraction emitting @ 10,000 ppmv
4	15	1,385	52	16	1.16%
5	13	1,134	60	17	1.50%
6	20	2,065	65	19	0.92%
7	11	1,257	58	22	1.75%
8	14	1,368	89	38	2.78%
9	8	1,017	45	13	1.28%
10	15	2,417	76	22	0.91%
11	8	962	50	21	2.18%
12	13	1,248	42	15	1.20%

Supplementary Table 44: Eastern dataset component-level emission factors

Component	Count	Fraction emitting @ 10,000 ppmv	Sampled with HFS	HFS leak rate [kg/day]
Connector	10,325	1.12%	65	0.07
Valve	2,310	2.16%	24	0.54
Open Ended Line	84	15.48%	10	0.12
Other	134	2.99%	3	0.76

8. Supplementary Figures

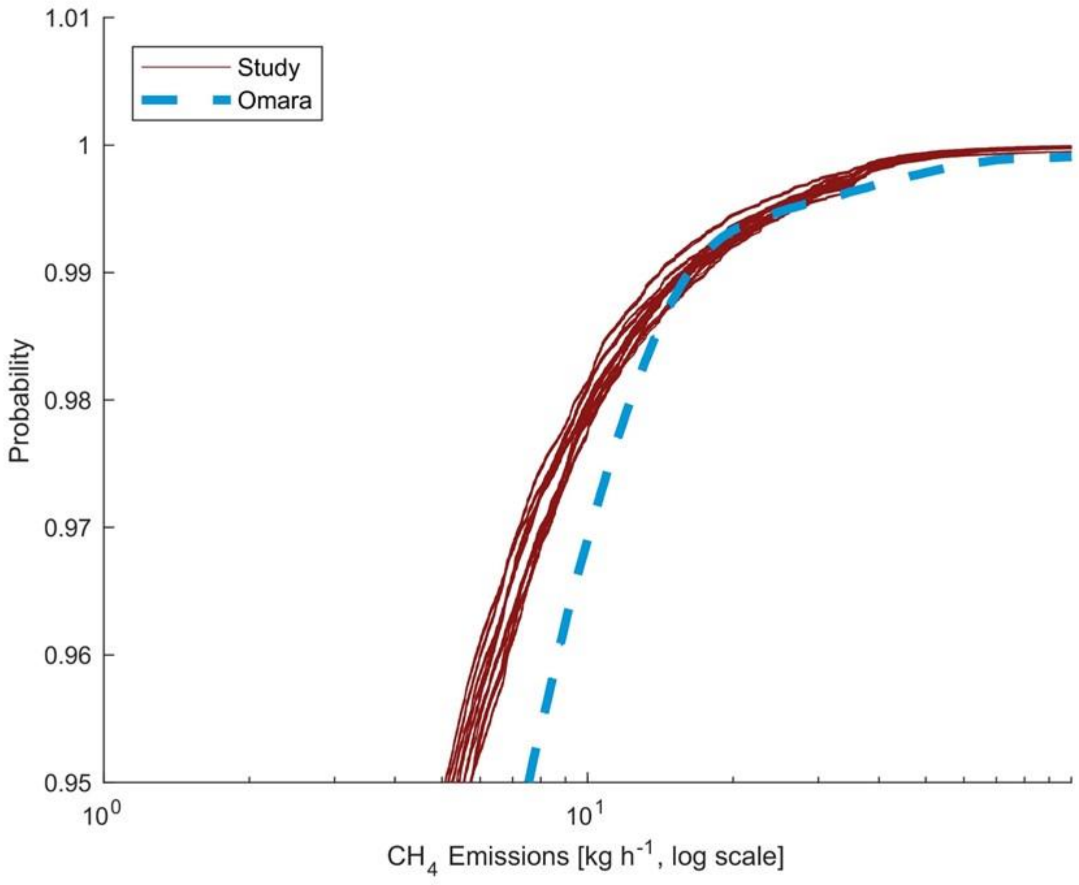

Supplementary Fig. 35: Cumulative distribution plot of CH_4 emissions per well-site zoomed-in near the superemitter range. 100 Monte Carlo simulations of this study (red lines) are compared against results from Omara et al. [6] (blue line).

9. Supplementary References

1. Buendia, E. *et al. Volume 2, Chapter 4: Fugitive Emissions - 2019 Refinement to the 2006 IPCC Guidelines for National Greenhouse Gas Inventories*. (2019).

2. Code of Federal Regulations. *Title 40 Part 60 Subpart OOOOa, Standards of Performance for Crude Oil and Natural Gas Facilities for which Construction, Modification or Reconstruction Commenced After September 18, 2015*.

3. Code of Federal Regulations. *Title 40 Part 98 Subpart W, Petroleum and Natural Gas Systems*. (2010).

4. Alvarez, R. A. *et al.* Assessment of methane emissions from the US oil and gas supply chain. *Science (80-.).* eaar7204 (2018).

5. (EPA) Environmental Protection Agency. *Inventory of U.S. Greenhouse Gas Emissions and Sinks: 1990 - 2018*. (2020).

6. Omara, M. *et al.* Methane Emissions from Natural Gas Production Sites in the United States: Data Synthesis and National Estimate. *Environ. Sci. Technol.* (2018) doi:10.1021/acs.est.8b03535.

7. Brantley, H. L., Thoma, E. D., Squier, W. C., Guven, B. B. & Lyon, D. Assessment of methane emissions from oil and gas production pads using mobile measurements. *Environ. Sci. Technol.* (2014) doi:10.1021/es503070q.

8. Robertson, A. M. *et al.* Variation in Methane Emission Rates from Well Pads in Four Oil and Gas Basins with Contrasting Production Volumes and Compositions. *Environ. Sci. Technol.* (2017) doi:10.1021/acs.est.7b00571.

9. Goetz, J. D. *et al.* Atmospheric emission characterization of marcellus shale natural gas development sites. *Environ. Sci. Technol.* (2015) doi:10.1021/acs.est.5b00452.

10. Omara, M. *et al.* Methane Emissions from Conventional and Unconventional Natural Gas Production Sites in the Marcellus Shale Basin. *Environ. Sci. Technol.* (2016) doi:10.1021/acs.est.5b05503.

11. Yacovitch, T. I. *et al.* Mobile Laboratory Observations of Methane Emissions in the Barnett Shale Region. *Environ. Sci. Technol.* (2015) doi:10.1021/es506352j.

12. (ERG) Eastern Research Group. *City of Fort Worth Natural Gas Air Quality Study*. (2011).

13. Lan, X., Talbot, R., Laine, P. & Torres, A. Characterizing Fugitive Methane Emissions in the Barnett Shale Area Using a Mobile Laboratory. *Environ. Sci. Technol.* (2015) doi:10.1021/es5063055.

14. Rella, C. W., Tsai, T. R., Botkin, C. G., Crosson, E. R. & Steele, D. Measuring emissions from oil and natural gas well pads using the mobile flux plane technique. *Environ. Sci. Technol.* (2015) doi:10.1021/acs.est.5b00099.

15. Star Environmental. *Fugitive hydrocarbon emissions from oil and gas production operations. API Publication 4589*. (1993).

16. Star Environmental. *Fugitive Hydrocarbon Emissions: Eastern Gas Wells*. (1995).

17. (EPA) Environmental Protection Agency. *Protocol for Equipment Leak Emission Estimates. Report No. EPA-453/R-95-017*. (1995).

18. Mitchell, A. L. *et al.* Measurements of methane emissions from natural gas gathering facilities and processing plants: Measurement results. *Environ. Sci. Technol.* **49**, 3219–3227 (2015).

19. Zimmerle, D. J. *et al.* Methane emissions from the natural gas transmission and storage system in the United States. *Environ. Sci. Technol.* **49**, 9374–9383 (2015).

20. Omara, M. *et al.* Methane Emissions from Natural Gas Production Sites in the United States: Data Synthesis and National Estimate. *Environ. Sci. Technol.* **52**, 12915–12925 (2018).

21. Greenpath Energy Ltd. Historical Canadian Fugitive Emissions Management Program Assessment. (2017).

22. Clearstone Engineering Ltd. *Update of Equipment, Component and Fugitive Emission Factors for Alberta Upstream Oil and Gas*. (2018).

23. Pacsi, A. P. *et al.* Equipment leak detection and quantification at 67 oil and gas sites in the Western United States. *Elementa* (2019) doi:10.1525/elementa.368.

24. Allen, D. T. *et al.* Measurements of methane emissions at natural gas production sites in the United States. *Proc. Natl. Acad. Sci. U. S. A.* (2013) doi:10.1073/pnas.1304880110.

25. Bell, C. S. *et al.* Comparison of methane emission estimates from multiple measurement techniques at natural gas production pads. *Elementa* (2017) doi:10.1525/elementa.266.

26. Allen, D. T. *et al.* Methane emissions from process equipment at natural gas production sites in the United States: Pneumatic controllers. *Environ. Sci. Technol.* (2015) doi:10.1021/es5040156.

27. Luck, B. *et al.* Multiday Measurements of Pneumatic Controller Emissions Reveal the Frequency of Abnormal Emissions Behavior at Natural Gas Gathering Stations. *Environ. Sci. Technol. Lett.* (2019) doi:10.1021/acs.estlett.9b00158.

28. Hawkeye Industries Inc. Tank Venting, A Guide to Venting of Upstream Petroleum Storage Tanks and Solutions, Technical Bulletin TB-1217-TV. https://hawk-eye.com/wp-content/uploads/2019/06/TB-1217-TV.pdf (2019).

29. Zimmerle, D. *et al. Characterization of Methane Emissions from Gathering Compressor Stations: Final Report*. (2019).

30. (EPA) Environmental Protection Agency. *Revisions to Natural Gas and Petroleum Systems Production Emissions*. https://www.epa.gov/sites/production/files/2017-

04/documents/2017_ng-petro_production.pdf (2017).

31. Zavala-Araiza, D. *et al.* Super-emitters in natural gas infrastructure are caused by abnormal process conditions. *Nat. Commun.* (2017) doi:10.1038/ncomms14012.

32. Hendler, A., Nunn, J., Lundeen, J. & McKaskle, R. *VOC emissions from oil and condensate storage tanks*. (2009).

33. (ERG) Eastern Research Group. *Condensate Tank Oil and Gas Activities*. (2012).

34. Allen, D. T. *et al.* Methane emissions from process equipment at natural gas production sites in the United States: Liquid unloadings. *Environ. Sci. Technol.* (2015) doi:10.1021/es504016r.

35. (EPA) Environmental Protection Agency. Greenhouse gas customized search. https://www.epa.gov/enviro/greenhouse-gas-customized-search.

36. (EPA) Environmental Protection Agency. Inventory of U.S. Greenhouse Gas Emissions and Sinks 1990-2017: Other Updates Considered for 2019 and Future GHGIs. https://www.epa.gov/sites/production/files/2019-04/documents/2019_ghgi_updates_-_other_updates_2019-04-10.pdf (2019).

37. (EPA) Environmental Protection Agency. Inventory of U.S. Greenhouse Gas Emissions and Sinks 1990-2016: Revisions to Create Year-Specific Emissions and Activity Factors. https://www.epa.gov/sites/production/files/2018-04/documents/ghgemissions_year_specific_2018.pdf (2018).

38. (EPA) Environmental Protection Agency. *Additional Revisions Considered for 2018 and Future GHGIs*. www.epa.gov/sites/production/files/2018-04/documents/ghgemissions_additional_revisions_2018.pdf (2018).

39. Gas Technology Institute. *Gas Resource Database: Unconventional Natural Gas and Gas Composition Databases*. (2001).

40. Enervus. Enervus Exploration and Production. https://www.enverus.com/industry/exploration-and-production/.

41. (EPA) Environmental Protection Agency. Revision to Well Counts Data. https://www.epa.gov/sites/production/files/2015-12/documents/revision-data-source-well-counts-4-10-2015.pdf (2015).

42. Al-Shammasi, A. A. A review of bubblepoint pressure and oil formation volume factor correlations. *SPE Reserv. Eval. Eng.* (2001) doi:10.2118/71302-pa.

43. Fanchi, J. R. *Petroleum Engineering Handbook, Volume I: General Engineering*. *Petroleum Engineering Handbook* (2006).

44. New Source Performance Standard. Stationary Engines: SI Engines (NSPS). C.F.R., Title 40, Part 60, Subpart JJJJ. (2008).

45. Zhizhin, M. Five-year Survey of the U.S. Natural Gas Flaring Observed from Space with

VIIRS. https://www.esrl.noaa.gov/gmd/publications/annual_meetings/2017/slides/5-Zhizhin.pdf (2017).

46. (EIA) Energy Information Administration. *Natural Gas Annual*. (2019).

47. Gvakharia, A. *et al.* Methane, Black Carbon, and Ethane Emissions from Natural Gas Flares in the Bakken Shale, North Dakota. *Environ. Sci. Technol.* (2017) doi:10.1021/acs.est.6b05183.

48. (EDF) Environmental Defence Fund. Methodology: Permian Methane Analysis Project. https://www.edf.org/sites/default/files/documents/PermianMapMethodology_1.pdf (2020).

49. Lyon, D. R. *et al.* Aerial Surveys of Elevated Hydrocarbon Emissions from Oil and Gas Production Sites. *Environ. Sci. Technol.* (2016) doi:10.1021/acs.est.6b00705.

50. Zavala-Araiza, D. *et al.* Methane emissions from oil and gas production sites in Alberta, Canada. *Elementa* (2018) doi:10.1525/elementa.284.

51. Thoma, E. D. *et al.* Assessment of Uinta Basin Oil and Natural Gas Well Pad Pneumatic Controller Emissions. *J. Environ. Prot. (Irvine,. Calif).* (2017) doi:10.4236/jep.2017.84029.

52. (EPA) Environmental Protection Agency. Overview of Greenhouse Gases. https://www.epa.gov/ghgemissions/overview-greenhouse-gases.

53. (EIA) Energy Information Administration. Natural Gas Gross Withdrawals and Production. https://www.eia.gov/dnav/ng/ng_prod_sum_dc_nus_mmcf_a.htm (2020).

54. (EPA) Environmental Protection Agency. *Overview of Update to Methodology for Hydraulically Fractured Gas Well Completions and Workovers*. https://19january2017snapshot.epa.gov/sites/production/files/2015-12/documents/overview-of-updated-inventory-methodology-for-fractured-gas-well-completions.pdf (2014).

55. (EPA) Environmental Protection Agency. *Revisions to Natural Gas and Petroleum Production Emissions*. https://www.epa.gov/sites/production/files/2016-08/documents/final_revision_to_production_segment_emissions_2016-04-14.pdf (2016).

56. (EPA) Environmental Protection Agency. *Inventory of Greenhouse Gas Emissions and Sinks*. (2015).

57. (EPA) Environmental Protection Agency. *Inventory of U.S. Greenhouse Gas Emissions and Sinks: 1990 - 2014*. (2016).

58. (EPA) Environmental Protection Agency. *Inventory of U.S Greenhouse Gas Emissions and Sinks: 1990 - 2015*. (2017).

59. (EPA) Environmental Protection Agency. *GREENHOUSE GAS EMISSIONS REPORTING FROM THE PETROLEUM AND NATURAL GAS INDUSTRY BACKGROUND TECHNICAL SUPPORT DOCUMENT*. (2015).

60. Campbell, L. *et al. Methane Emissions from the Natural Gas Industry. Volume 12: Pneumatic Controllers*. (1996).

61. Campbell, L., Campbell, M., Cowgill, M., Epperson, D., Hall, M., Harrison, M., Hummel, K., Myers, D., Shires, T., Stapper, B., Stapper, C., Wessel, J. *Methane emissions from the natural gas industry. Volume 11: Compressor driver exhaust*. (1996).

62. Star Environmental. *Emission factors for oil and gas production operations. API Publication 4615*. (1995).

63. Hummel, K.E., Campbell, L.M. and Harrison, M. R. *Methane Emissions from the Natural Gas Industry. Volume 8. Equipment Leaks*. (1996).

64. Star Environmental. *Calculation Workbook for Oil and Gas Production Equipment Fugitive Emissions. API Publication 4638*. (1996).

65. (EPA) Environmental Protection Agency. *Inventory of U.S. Greenhouse Gas Emissions and Sinks: 1990-2007*. (2009).

66. (EPA) Environmental Protection Agency. *Inventory of U.S. Greenhouse Gas Emissions and Sinks: 1990 - 2008*. (2010).

67. (EPA) Environmental Protection Agency. *Inventory of U.S. Greenhouse Gas Emissions and Sinks: 1990 - 2009*. (2011).

68. (EPA) Environmental Protection Agency. *Inventory of U.S. Greenhouse Gas Emissions and Sinks: 1990 - 2010*. (2012).

69. (USEPA) US Environmental Protection Agency. *Inventory of U.S. Greenhouse Gas Emissions and Sinks: 1990 - 2011*. (2013).

70. (EPA) Environmental Protection Agency. *Inventory of U.S. Greenhouse Gas Emissions and Sinks: 1990 - 2012*. (2014).

71. (EPA) Environmental Protection Agency. *Inventory of U.S. Greenhouse Gas Emissions and Sinks: 1990 - 2016*. (2018).

72. (EPA) Environmental Protection Agency. *Inventory of Greenhouse Gas Emissions and Sinks*. (2019).

73. Radian Corporation. *Study of Refinery Fugitive Emissions from Equipment Leaks, Volumes I, II, and III*. (1994).

74. Radian Corporation. *Development of Fugitive Emission Factors and Emission Profiles for Petroleum Marketing Terminals, Volumes I and II, API 4588*. (1993).

75. (API) American Petroleum Institute. *PRODUCTION TANK EMISSIONS MODEL - A PROGRAM FOR ESTIMATING EMISSIONS FROM HYDROCARBON PRODUCTION TANKS - E&P TANK VERSION 2.0*. (2000).

76. Gidney, B. & Pena, S. *Upstream Oil and Gas Storage Tank Project Flash Emissions Models Evaluation*. (2009).

77. aspentech. *HYSYS 2004 Simulation basis*. (2004).

78. (EPA) Environmental Protection Agency. Revisions to CO2 Emissions Estimation Methodologies. https://www.epa.gov/sites/production/files/2018-04/documents/ghgemissions_co2_2018.pdf (2018).

79. (EPA) Environmental Protection Agency. *Compliance Alert: EPA Observes Emissions from Controlled Storage Vessels at Onshore Oil and Natural Gas Producton Facilites*. (2015).

80. Lyman, S. N., Tran, T., Mansfield, M. L. & Ravikumar, A. P. Aerial and ground-based optical gas imaging survey of Uinta Basin oil and gas wells. *Elementa* (2019) doi:10.1525/elementa.381.

81. Mansfield, Marc L., Lyman, S., O'Neil, T., Anderson, R., Jones, C., Tran, H., Mathis, J., Barickman, P., Oswald, W., LeBaron, B. *STORAGE TANK EMISSIONS PILOT PROJECT (STEPP): FUGITIVE ORGANIC COMPOUND EMISSIONS FROM LIQUID STORAGE TANKS IN THE UINTA BASIN*. (2017).

82. Englander, J. G., Brandt, A. R., Conley, S., Lyon, D. R. & Jackson, R. B. Aerial Interyear Comparison and Quantification of Methane Emissions Persistence in the Bakken Formation of North Dakota, USA. *Environ. Sci. Technol.* (2018) doi:10.1021/acs.est.8b01665.

83. Cook, C. & Dunham, S. *Greenhouse gas reporting rule: Leak Detection methodology revisions and confidentiality determinations for petroleum and natural gas systems*. *Federal Register* vol. 81 9797 (2016).

84. (EPA) Environmental Protection Agency. Confluence: GHG Reporting Instructions: Subpart W Flares and Flare Stacks. https://ccdsupport.com/confluence/display/help/Subpart+W+Flares+and+Flare+Stacks.

85. Ravikumar, A. P. *et al.* Repeated leak detection and repair surveys reduce methane emissions over scale of years. *Environ. Res. Lett.* (2020) doi:10.1088/1748-9326/ab6ae1.

86. (ERG) Eastern Research Group. *City of Fort Worth Natural Gas Air Quality Study: Revised Final Point Source Test Plan*. (2010).

87. Kirchgessner, D. A., Lott, R. A., Cowgill, R. M., Harrison, M. R. & Shires, T. M. Estimate of methane emissions from the U.S. natural gas industry. *Chemosphere* (1997) doi:10.1016/S0045-6535(97)00236-1.
